# Osteosarcoma Stem Cell Potent Gallium(III)‐Polypyridyl Complexes Bearing Diflunisal

**DOI:** 10.1002/chem.202102207

**Published:** 2021-08-20

**Authors:** Zhiyin Xiao, Ginevra Passeri, Joshua Northcote‐Smith, Kuldip Singh, Kogularamanan Suntharalingam

**Affiliations:** ^1^ School of Chemistry University of Leicester Leicester UK; ^2^ College of Biological, Chemical Sciences and Engineering Jiaxing University Jiaxing China

**Keywords:** antitumour agents, COX-2 inhibition, DNA damage, gallium, osteosarcoma stem cells

## Abstract

We report the anti‐osteosarcoma stem cell (OSC) properties of a series of gallium(III)‐polypyridyl complexes (**5**‐**7**) containing diflunisal, a non‐steroidal anti‐inflammatory drug. The most effective complex within the series, **6** (containing 3,4,7,8‐tetramethyl‐1,10‐phenanthroline), displayed similar potency towards bulk osteosarcoma cells and OSCs, in the nanomolar range. Remarkably, **6** exhibited significantly higher monolayer and sarcosphere OSC potency (up to three orders of magnitude) than clinically approved drugs used in frontline (cisplatin and doxorubicin) and secondary (etoposide, ifosfamide, and carboplatin) osteosarcoma treatments. Mechanistic studies show that **6** downregulates cyclooxygenase‐2 (COX‐2) and kills osteosarcoma cells in a COX‐2 dependent manner. Furthermore, **6** induces genomic DNA damage and caspase‐dependent apoptosis. To the best of our knowledge, **6** is the first metal complex to kill osteosarcoma cells by simultaneously inhibiting COX‐2 and damaging nuclear DNA.

## Graphical abstract

**Breaking ‘hard‐to‐kill’ bone cancer cells with gallium**: Osteosarcoma stem cells (OSCs) are linked to the poor prognosis of metastasised osteosarcoma. Here a gallium(III)‐polypyridyl complex is reported, containing the non‐steroidal anti‐inflammatory drug, diflunisal, capable of killing OSCs more effectively than frontline and secondary anti‐osteosarcoma drugs. The complex evokes cell death by inhibiting cyclooxygenase‐2 and damaging nuclear DNA.

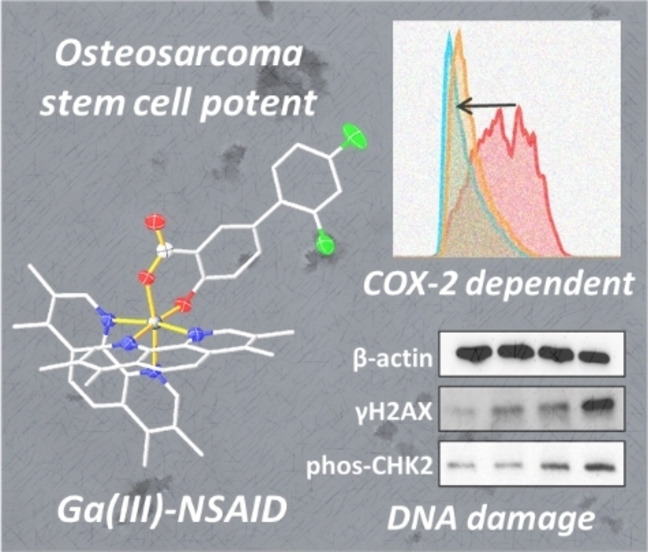

## Introduction

Osteosarcoma is the most common type of bone cancer in children and young people.^[1]^ After diagnosis and staging, current osteosarcoma treatment usually involves a combination of aggressive chemotherapy, specialised surgery, and sometimes radiation (depending the site of the osteosarcoma).^[2]^ The 5‐year survival rate for osteosarcoma patients of all ages dramatically changes depending on the stage of the disease. Patients with localised osteosarcoma can expect 5‐year survival rates as high as 74 %, but if metastasised, survival rates drop to 27 %.^[3]^ The poor prognosis of metastasised osteosarcoma has been heavily linked to the existence of osteosarcoma stem cells (OSCs).^[4]^ OSCs are a small sub‐population of osteosarcomas with the ability to differentiate and self‐renew.^[4]^ OSCs divide slower than bulk osteosarcoma cells and thus can overcome frontline anti‐osteosarcoma chemotherapy (doxorubicin, cisplatin, and methotrexate) and radiation regimens which tend to target fast growing cells.[^4c^, ^5^] The very low proportion of OSCs within a given osteosarcoma site and their tendency to reside in hard to reach niches, means that they can be missed by surgery as well. After surviving treatment, OSCs can reform osteosarcoma in the primary site and promote its spread around the body.[^4b^, ^4c^, ^5a^, ^6^] The clinical implication of OSCs means that osteosarcoma treatments must have the ability to remove heterogeneous osteosarcoma populations in their entirety, including OSCs, otherwise OSC‐mediated relapse could occur over time. Potential OSC therapeutic targets such as cell surface markers, deregulated signalling pathways, and components within the microenvironments in which they reside have been identified but there is still no clinically approved drug that can fully remove OSCs.^[4]^ Therefore the development of new chemotherapeutic agents capable of removing bulk osteosarcoma cells and residual OSCs could improve the survival outcomes of osteosarcoma patients in the long‐term.

**Figure 1:**
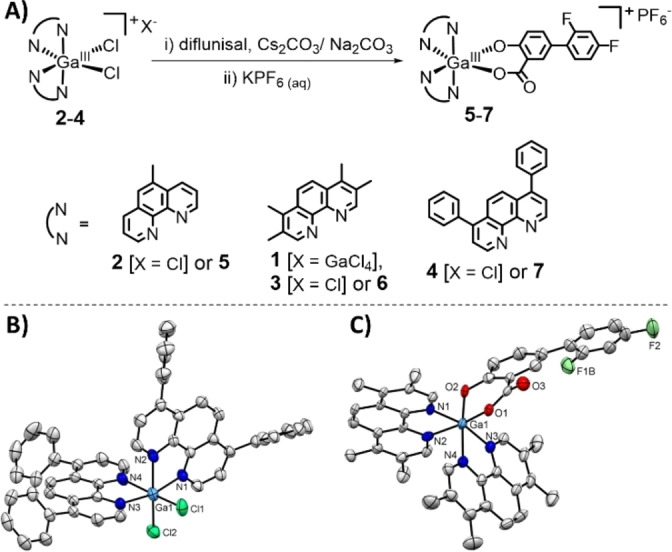
(A) Reaction scheme for the preparation of the gallium(III)‐polypyridyl complexes bearing diflunisal **5**–**7** from the corresponding *cis*‐dichlorobis‐(polypyridyl) gallium(III) chloride complexes **2**–**4**. The chemical structure of the previously reported gallium(III) complex **1** is also shown. The cationic component of **1** contains two 3,4,7,8‐tetramethyl‐1,10‐phenanthroline ligands and two chloride ligands bound to a gallium(III) metal centre. The anionic component of **1** is a gallium(III) tetrachloride ion. (B) X‐ray structure of **4** comprising of two 4,7‐diphenyl‐1,10‐phenanthroline ligands and two chloride ligands. The chloride counter‐anion and the co‐crystallising solvent molecules (dichloromethane and methanol) have been omitted for clarity. (C) X‐ray structure of **6** comprising of two 3,4,7,8‐tetramethyl‐1,10‐phenanthroline ligands and diflunisal. The hexafluorophosphate counter‐anion and the co‐crystallising solvent molecules (acetonitrile and water) have been omitted for clarity. For (B) and (C), ellipsoids are shown at 50 % probability. Cl atoms are shown in dark green, N in dark blue, C in grey, O in red, F in light green, and Ga in light blue.

Reports on the development of new chemotherapeutics and the application of established chemotherapeutics to overcome OSCs are rare, and have largely focused on purely organic small molecules.[^4c^, ^7^] We recently reported the first metal‐based agents to potently kill OSCs in vitro.^[8]^ Specifically, a series of gallium(III) complexes containing polypyridyl ligands were found to kill bulk osteosarcoma cells and OSCs (in monolayer and three‐dimensional cell culture systems) within the micro‐ to nano‐molar range.^[8]^ Our rationale for investigating the anti‐OSC properties of gallium(III) compounds was inspired by: (i) the intrinsic ability of gallium(III) salts to accumulate in bones, and (ii) the favourable safety profiles observed for gallium(III) compounds that have undergone human clinical trials (such as KP46 and gallium maltolate).^[9]^ The most effective gallium(III) complex in the previously reported series **1** (see Figure 1A for chemical structure of **1**) was over 400‐fold more potent than cisplatin towards methotrexate (MTX)‐resistant OSCs, and significantly less toxic towards a panel of non‐cancerous cells of various tissue types (lung, breast, skin, and kidney).^[8]^ Mechanistic studies showed that **1** induced apoptotic osteosarcoma cell death by entering the nucleus and damaging genomic DNA.^[8]^ In the current study we have carried out key chemical modifications to this promising class of gallium(III)‐polypyridyl complexes, in an attempt to improve their biocompatibility and OSC activity. The two (relatively labile) chloride ligands attached to the gallium(III) centre in **1** were substituted with diflunisal, a nonsteroidal anti‐inflammatory drug (NSAIDs). Diflunisal was envisaged to bind to the gallium(III) centre in a bidentate manner (via the salicylate moiety). Diflunisal is a potent inhibitor of cyclooxygenase isoenzymes, COX‐1 and COX‐2.^[10]^ Cyclooxygenases catalyse the biosynthesis of prostaglandins (PGs), which are mediators in inflammatory reactions.^[11]^ The inducible isoform, COX‐2 is overexpressed in certain OSCs, and is thought to play an important role in osteosarcoma initiation, growth, progression, and spread.^[12]^ Therefore, inhibition of COX‐2 using diflunisal could provide an effective method of sensitising OSCs to cytotoxic agents. Furthermore, we used a variety of polypyridyl ligands to form the gallium(III)‐polypyridyl core (namely 5‐methyl‐1,10‐phenanthroline, 3,4,7,8‐tetramethyl‐1,10‐phenanthroline, and 4,7‐diphenyl‐1,10‐phenanthroline) to modulate lipophilicity, solution stability, and OSC activity. Additionally, the gallium(III) tetrachloride counter‐anion present in **1**, which could be biologically reactive, was replaced with relatively innocent anions. Herein we report the synthesis, characterisation, solution stability, and in vitro potency towards bulk osteosarcoma cells and OSCs, of a new series of gallium(III)‐polypyridyl complexes bearing diflunisal.

## Results and Discussion

### Synthesis and characterisation

The gallium(III) complexes **2**–**7** investigated in this study are depicted in Figure 1A. The *cis*‐dichlorobis‐(polypyridyl) gallium(III) chloride complexes **2**–**4** were prepared by reacting gallium(III) chloride with two equivalents of the appropriate polypyridyl ligand (5‐methyl‐1,10‐phenanthroline for **2**, 3,4,7,8‐tetramethyl‐1,10‐phenanthroline for **3**, or 4,7‐diphenyl‐1,10‐phenanthroline for **4**) in dry dichloromethane, under nitrogen. Upon addition of diethyl ether, the gallium(III) complexes **2**–**4** were isolated as white solids in high yields (79–95 %). The gallium(III) complexes **2**–**4** were fully characterised by ^1^H and ^13^C{^1^H} NMR, infrared spectroscopy, high resolution ESI mass spectrometry, and elemental analysis (Figures S1‐S10). The aromatic proton signals in the ^1^H NMR spectra of **2**–**4** were shifted relative to the corresponding polypyridyl ligands indicative of metal coordination (Figures S1, S3, S5, S11–S13). Furthermore, the fact that the polypyridyl ligand proton signals in **2**–**4** were inequivalent suggested that a single *cis* isomer was isolated in each case (Figures S1, S3, S5). Distinctive molecular ion peaks corresponding to **2**–**4** with the appropriate isotopic pattern were observed in the positive mode of the ESI mass spectra (*m*/*z*=529.0303 amu, [**2**‐Cl]^+^; 613.1260 amu, [**3**‐Cl]^+^; 805.1224 amu, [**4**‐Cl]^+^), providing further evidence for the formation of **2**–**4** (Figures S8‐S10). The purity of **2**–**4** was confirmed by elemental analysis. The structure of **4** was further confirmed by X‐ray diffraction studies and is shown in Figure 1B. Crystals of **4** suitable for an X‐ray diffraction analysis were obtained by slow diffusion of diethyl ether into a concentrated methanol‐dichloromethane solution of **4** (CCDC 2086778, Figure 1B, and Table S1). Selected bond distances and bond angles are presented in Table S2. The cationic component of the complex exhibits a distorted octahedral geometry with the gallium(III) centre coordinated to two chloride ligands in a *cis*‐orientation and two 4,7‐diphenyl‐1,10‐phenanthroline ligands, each forming a five membered chelated ring. The average Ga−N (2.10 Å) and Ga−Cl (2.28 Å) bond distances are consistent with bond parameters for related gallium(III) complexes.[^8^, ^13^]

The diflunisal‐containing gallium(III) complexes **5**–**7** were prepared by reacting the appropriate *cis*‐dichlorobis‐(polypyridyl) gallium(III) chloride complex **2**–**4** with equimolar amounts of diflunisal in methanol or dichloromethane, in the presence of CsCO_3_ or NaCO_3_. After conversion to the corresponding hexafluorophosphate salt (using potassium hexafluorophosphate), the crude product was recrystallised in dichloromethane to isolate pure **5**–**7** as white solids in high yields (77–91 %). The gallium(III) complexes **5**–**7** were fully characterised by ^1^H, ^19^F{^1^H}, ^31^P{^1^H} NMR, infrared spectroscopy, high resolution ESI mass spectrometry, and elemental analysis (Figures S14–S26). Attachment of diflunisal to the gallium(III) centre in **5**–**7** via the two hydroxyl groups within the salicylate moiety was confirmed by the disappearance of the broad hydroxyl band (at 3086 cm^−1^ for diflunisal) in the IR spectra of **5**–**7** (Figures S14). Additionally, the IR spectra for **5**–**7** displayed ν_asym_(CO_2_) and ν_sym_(CO_2_) stretching bands at 1628–1633 cm^−1^ and 1427–1430 cm^−1^, respectively (Figures S14). The difference, Δ, between the ν_asym_(CO_2_) and ν_sym_(CO_2_) stretching bands for **5**–**7** varied between 201–203 cm^−1^, suggestive of an unidentate coordination mode for the carboxylate group (within the salicylate moiety) on diflunisal to the gallium(III) centre.^[14]^ This assignment is consistent with diflunisal binding to the gallium(III) centre in a bidentate fashion via the two hydroxyl groups (as depicted in Figure 1A). Furthermore, the aromatic proton signals in the ^1^H NMR spectra of **5**–**7** shifted relative to diflunisal, indicative of metal coordination (Figures S15, S18, S21, S27). The fact that only one set of ^1^H and ^19^F{^1^H} NMR signals were observed for diflunisal in **5**–**7** suggests that a single diastereoisomer was isolated in each case (Figures S15–S16, S18–S19, S21–S22). Distinctive molecular ion peaks corresponding to **5**–**7** with the appropriate isotopic pattern were observed in the positive mode of the ESI mass spectra (*m*/*z*=705.1223 amu, [**5**‐PF_6_]^+^; 789.2155 amu, [**6**‐PF_6_]^+^; 981.2153 amu, [**7**‐PF_6_]^+^), providing further evidence for product formation (Figures S24‐S26). The purity of **5**–**7** was established by elemental analysis. Crystals of **6** suitable for an X‐ray diffraction analysis were obtained by slow diffusion of diethyl ether into an acetonitrile solution of **6** (CCDC 2086777, Figure 1C, Table S3). Selected bond distances and bond angles are presented in Table S4. The cationic component of the complex exhibits a distorted octahedral geometry with the gallium(III) centre coordinated to two 3,4,7,8‐tetramethyl‐1,10‐phenanthroline ligands and diflunisal via the two hydroxyl groups. The gallium(III)‐coordinated diflunisal is slightly disordered due to the rotational flexibility of the 2,4‐difluorophenyl unit. The gallium(III) coordination sphere is consistent with the aforementioned spectroscopic and analytical data for **6**. The average Ga−N (2.09 Å) and Ga−O (1.90 Å) bond distances are consistent with bond parameters for related gallium(III) complexes.[^8^, ^13^, ^15^]

### Lipophilicity and solution stability

The lipophilicity of the diflunisal‐containing gallium(III) complexes **5**–**7** was experimentally determined by measuring the extent to which they partitioned between octanol and water, using UV‐Vis spectroscopy. The LogP values of **5**–**7** varied between 0.04±0.02 and 0.14±0.03 (Table S5). The amphiphilic nature of **5**–**7** indicates that the gallium(III) complexes should be able to readily enter cells and have reasonable aqueous solubility.

UV‐Vis spectroscopy and ESI mass spectrometry studies were performed to determine the stability of the gallium(III) complexes **5**–**7** in various solutions relevant for subsequent cell‐based studies. In DMSO or H_2_O:DMSO (200 : 1) the absorbance and wavelengths of the bands associated to **5**–**7** (25 μM) remained largely unaltered over the course of 24 h at 37 °C, indicative of stability (Figure S29–S34). In PBS:DMSO (200 : 1) the absorbance and wavelengths of the bands associated to **5** and **6** (25 μM) remained largely unchanged over the course of 24 h at 37 °C, suggestive of stability under these conditions (Figures S35–S36). In contrast, the absorbance of the bands associated to the 4,7‐diphenyl‐1,10‐phenanthroline‐bearing complex **7** dramatically decreased over the course of 24 h at 37 °C in PBS:DMSO (200 : 1), suggestive of instability (Figure S37). Given the instability of **7** in PBS:DMSO (200 : 1) further stability (and cell‐based) studies were not performed with this complex. The UV‐Vis spectra of diflunisal, 5‐methyl‐1,10‐phenanthroline, 3,4,7,8‐tetramethyl‐1,10‐phenanthroline, and 4,7‐diphenyl‐1,10‐phenanthroline (25 μM) at 37 °C in PBS:DMSO (200 : 1) were measured as controls (Figure S38). To probe the solution stability of **5** and **6** further, the gallium complexes (25 μM) were incubated with 10 equivalents of ascorbic acid or glutathione (cellular reductants) in PBS:DMSO (200 : 1) and monitored by UV‐Vis spectroscopy for 24 h at 37 °C. The UV‐Vis traces for **5** and **6** were only moderately modified under these conditions, implicative of reasonable stability (Figures S39‐S42). Upon evaluation of a H_2_O:DMSO (10 : 1) solution of **5** or **6** (500 μM) in the presence of ascorbic acid or glutathione (10 equivalents) by ESI mass spectrometry, distinct peaks corresponding to the molecular ion of **5** and **6** with the expected isotopic distribution (*m*/*z*=705 amu, [**5**‐PF_6_]^+^; 789 amu, [**6**‐PF_6_]^+^) were observed before and after incubation for 24 h at 37 °C (Figures S43–S46). Collectively, this suggests that **5** and **6** are both stable and remain intact under biologically reducing conditions. In sodium acetate buffer solution (pH 5.2):DMSO (200 : 1) the absorbance and wavelengths of the bands associated to **5** and **6** (25 μM) remained generally unmodified over the course of 24 h at 37 °C, indicative of stability under acidic conditions (Figures S47‐S48). Before carrying out cellular studies, the stability of **5** and **6** in Dulbecco's Modified Eagle Medium (DMEM) was investigated (Figures S49–S50). The UV‐Vis traces of **5** and **6** (25 μM) in DMEM:DMSO (200 : 1) displayed only marginal changes over the course of 24 h at 37 °C. Therefore **5** and **6** were deemed suitably stable to progress to cell‐based studies.

### Bulk osteosarcoma cell and osteosarcoma stem cell potency in monolayer cultures

U2OS cells were used to determine the potency of the solution stable, diflunisal‐containing gallium(III) complexes **5** and **6** against bulk osteosarcoma cells and OSCs. U2OS cells are partially differentiated sarcoma‐derived cells that are amenable to standard cell culture methods. Previous work has shown that when grown under standard monolayer conditions, U2OS cell cultures typically comprise of <1% OSCs.^[5b]^ OSC sub‐populations in osteosarcoma cell cultures can be identified by their overexpression of the tyrosine kinase protein, CD117 on their cell surface.^[16]^ Using an established method, we isolated OSC‐enriched, CD117‐positive cells by treating U2OS cells with MTX (300 nM) for 4 days.[^5b^, ^8^] This method allowed us to generate OSC‐rich populations of U2OS cells, which we hereafter will refer to as U2OS‐MTX cells.

The cytotoxicity of **5** and **6** towards U2OS and U2OS‐MTX cells was assessed using the MTT [3‐(4,5‐dimethylthiazol‐2‐yl)‐2,5‐diphenyltetrazolium bromide] assay. IC_50_ values (concentration required to reduce cell viability by 50 %) were derived from dose‐response curves (Figures S51‐S52) and are summarised in Table 1. The gallium(III) complexes **5** and **6** displayed submicro‐ to nano‐molar potency towards U2OS and U2OS‐MTX cells. Both **5** and **6** killed OSC‐enriched U2OS‐MTX cells and OSC‐depleted U2OS cells with similar potency (Table 1). Therefore, **5** and **6** have the potential to eliminate whole osteosarcoma populations with a single submicro‐ or nano‐molar dose. Strikingly, the 3,4,7,8‐tetramethyl‐1,10‐phenanthroline‐containing complex **6** was 565‐ and 3.6‐fold more potent (*p*<0.05, n=18) towards OSC‐enriched U2OS‐MTX cells than frontline anti‐osteosarcoma drugs, cisplatin and doxorubicin respectively (Figure S53, Table 1).^[8]^ Furthermore **6** was 106‐ to >1666‐fold more potent (*p*<0.05, n=18) towards U2OS‐MTX cells than etoposide, ifosfamide, and carboplatin (Figures S54–S55, Table 1).^[8]^ Etoposide, ifosfamide, and carboplatin are clinically approved drugs used to treat relapsed osteosarcoma.^[17]^ Additionally, **6** was 25‐fold more potent (*p* <0.05, n=18) towards U2OS‐MTX cells than salinomycin, an established anti‐cancer stem cell (CSCs) agent (Table 1).^[8]^ It is also worth noting that that the potency of **6** towards U2OS‐MTX cells is greater than any metal complex tested under similar conditions.^[8]^

**Table 1:** IC_50_ values of the gallium complexes, **2**–**3** and **5**–**6**, diflunisal, 5‐methyl‐1,10‐phenanthroline, 3,4,7,8‐tetramethyl‐1,10‐phenanthroline, cisplatin, doxorubicin, etoposide, ifosfamide, carboplatin, and salinomycin against U2OS and U2OS‐MTX cells.

Compound	U2OS IC_50_ [μM] ^[a]^	U2OS‐MTX IC_50_ [μM] ^[a]^
**2**	0.87±0.09	1.09±0.10
**3**	0.06±0.01	0.17±0.02
**5**	0.36±0.09	0.39±0.04
**6**	0.08±0.01	0.06±0.02
diflunisal	>100	>100
5‐methyl‐1,10‐phenanthroline ^[b]^	0.62±0.01	0.98±0.02
3,4,7,8‐tetramethyl‐1,10‐phenanthroline ^[b]^	0.28±0.01	0.28±0.03
cisplatin ^[b]^	16.30±0.50	33.87±3.71
doxorubicin	0.17±0.01	0.22±0.07
etoposide	2.34±0.18	6.37±0.59
ifosfamide	>100	>100
carboplatin ^[b]^	>100	>100
salinomycin ^[b]^	6.09±1.06	1.49±0.26

[a] Determined after 72 h incubation (mean of three independent experiments±SD). [b] Reported in Ref. [8].

Control cytotoxicity studies showed that the potency of diflunisal and the corresponding *cis*‐dichlorobis‐(polypyridyl) gallium(III) chloride complexes **2** and **3** towards U2OS‐MTX cells was significantly lower than **5** and **6** (Table 1, Figures S56‐S58). More specifically, diflunisal was non‐toxic (IC_50_ value >100 μM), whereas **2** and **3** were 2.8‐fold less potent than their corresponding diflunisal‐containing analogues **5** and **6**. Further, free 5‐methyl‐1,10‐phenanthroline and 3,4,7,8‐tetramethyl‐1,10‐phenanthroline were 2.5‐ and 4.7‐fold less potent towards U2OS‐MTX cells than **5** and **6**, respectively (Table 1).^[8]^ Overall, this suggests that the cytotoxicity of **5** and **6** towards OSC‐enriched U2OS‐MTX cells is likely to result from the intact cellular entry of the gallium(III) complexes which allows for the synergistic co‐delivery of all the complex components.

### Effect on sarcosphere size and viability

**Figure 2:**
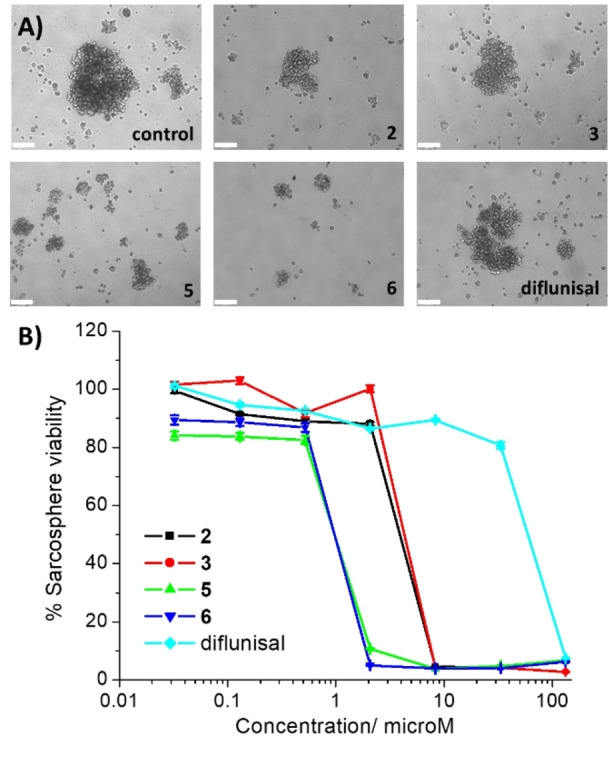
(A) Representative bright‐field images (×10) of U2OS‐MTX sarcospheres in the absence and presence of **2**–**3**, **5**–**6**, or diflunisal at its IC_20_ value (10 days incubation). Scale bar=180 μm. (B) Representative dose‐response curves for the treatment of U2OS‐MTX sarcospheres with **2**–**3**, **5**–**6**, or diflunisal after 10 days incubation.

The ability of the diflunisal‐containing gallium(III) complexes **5** and **6** to inhibit the formation of spheroids comprising of OSCs was investigated using the sarcosphere assay. Sarcospheres (sometimes referred to as osteospheres) are three‐dimensional tumour‐like structures that are formed by OSCs when grown under serum‐free, low attachment conditions.^[5b]^ Sarcospheres tend to exist as irregularly shaped free‐floating collections of OSCs (rather than uniform spherical structures) and their multicellular architecture means that they provide a good model for assessing OSC potency and in vivo potential. The addition of **5** and **6** (at the IC_20_ values) to single cell suspensions of U2OS‐MTX cells markedly reduced the size of sarcospheres formed after 10 days incubation (Figure 2A). Under the same conditions, the corresponding *cis*‐dichlorobis‐(polypyridyl) gallium(III) chloride complexes **2** and **3** (at the IC_20_ values for 10 days) also decreased the size of sarcospheres formed but to a lesser extent than **5** and **6** (Figure 2A). This implies that the presence of the diflunisal moiety in **5** and **6** enhances their ability to inhibit sarcosphere formation. Salinomycin (upon treatment at the IC_20_ value for 10 days) markedly reduced sarcosphere size but to a lesser extent than **5** and **6** (Figure S59). Under identical conditions, cisplatin and carboplatin (at the IC_20_ values for 10 days) did not noticeably affect sarcosphere size (Figure S59). Treatment with diflunisal (at the IC_20_ values for 10 days) moderately decreased sarcosphere size (Figures 2A).

Having shown that **5** and **6** are able to reduce the size of sarcospheres formed, their ability to affect viability (metabolic activity) was evaluated. This is an important consideration as the reduction in the size of sarcospheres formed does not necessarily mean that the sarcospheres are no longer viable. To assess the ability of **5** and **6** to reduce sarcosphere viability, the colorimetric resazurin‐based reagent, TOX8 was used. TOX8 has been previously proven to be a suitable reagent for determining tumour spheroid viability.^[18]^ The IC_50_ values, the concentration required to reduce sarcosphere viability by 50 %, were determined from dose‐response curves (Figure 2B) and are summarised in Table S6. The IC_50_ value of the gallium(III) complexes **5** and **6** towards sarcospheres was the same, 0.97±0.01 μM (Table S6). Notably, **5** and **6** displayed 4.8‐ to 23.5‐fold greater potency than cisplatin, carboplatin, and salinomycin (Table S6).^[8]^ The sarcosphere potency of the *cis*‐dichlorobis‐(polypyridyl) gallium(III) chloride complexes **2** and **3** was significantly (*p*<0.05) lower than **5** and **6** (Figure 2B, Table S6). This highlights the importance of the diflunisal moiety in **5** and **6** to their sarcosphere toxicity, and is consistent with the monolayer cytotoxicity results. Free diflunisal displayed micromolar potency towards sarcospheres, 61.6‐fold lower than **5** and **6** (Figures 2B, Table S6). This implies that the gallium(III)‐polypyridyl component in **5** and **6** also plays a vital role in the sarcosphere toxicity imparted by **5** and **6**. Collectively the sarcosphere studies indicate that **5** and **6** can inhibit the formation and reduce the viability of OSC‐enriched sarcospheres in three‐dimensional cell culture systems, within the submicro‐molar range.

### Cytotoxic mechanism of action in osteosarcoma cells

To provide greater insight into the cytotoxic mechanism of action of the gallium(III) complexes **5** and **6**, and to better understand the discrepancy in the overall osteosarcoma cell potency of **5** and **6** and the corresponding *cis*‐dichlorobis‐(polypyridyl) gallium(III) chloride complexes **2** and **3**, cellular uptake studies were performed. U2OS cells were dosed with **2**–**3** or **5**–**6** (0.25 μM for 24 h) and the internalised gallium content was determined by inductively coupled plasma mass spectrometry (ICP‐MS). The diflunisal‐containing gallium(III) complexes **5** (0.90±0.09 ng of Ga/ 10^6^ cells) and **6** (0.68±0.04 ng of Ga/10^6^ cells) were internalised by U2OS cells to a significantly (*p*<0.05) greater extent than the corresponding *cis*‐dichlorobis‐(polypyridyl) gallium(III) chloride complexes **2** (0.23±0.04 ng of Ga/10^6^ cells) and **3** (0.26±0.04 ng of Ga/10^6^ cells). The higher osteosarcoma cell uptake of **5** relative to **2**, and **6** relative to **3** could explain the variance in overall osteosarcoma cell potency of the complexes (with and without diflunisal, Table 1). It is worth noting that although the amount of gallium found inside treated U2OS cells was relatively low, this is reasonable considering the low administration dose (0.25 μM) used. The administration dose was set at a submicro‐molar concentration due to the high toxicity of the gallium(III) complexes (**2**–**3** and **5**–**6**) towards osteosarcoma cells. Further, nuclei isolation studies showed that a reasonable amount of internalised **5** (19 %, 0.17±0.01 ng of Ga/ 10^6^ cells) and **6** (5 %, 0.03±0.003 ng of Ga/10^6^ cells) could enter osteosarcoma cell nuclei, and thus gain access to genomic DNA. Therefore **5**‐ and **6**‐mediated osteosarcoma cell toxicity could be related to genomic DNA damage.

As our previous work on the structurally related gallium(III)‐polypyridyl complex **1** (Figure 1A) showed that it induced genomic DNA damage, and **5** and **6** can access osteosarcoma cell nuclei, the ability of **5** and **6** to activate biomarkers related to the DNA damage pathway was probed using immunoblotting methods. U2OS cells treated with **5** (0.18−0.70 μM for 24 h or 72 h) and **6** (22−66 nM for 24 h or 72 h) displayed a marked increase in the expression of the phosphorylated forms of H2AX and CHK2, implicative of DNA damage caused as a primary effect of **5** and **6** (Figures S60‐S63). When left unrepaired DNA damage can lead to apoptosis. U2OS cells treated with **5** (0.18−0.70 μM for 72 h) and **6** (22−66 nM for 72 h) displayed markedly higher levels of cleaved caspase 3, 7, and poly ADP ribose polymerase (PARP) compared to untreated cells (Figures S60–S61), characteristic of caspase‐dependent apoptosis. Monolayer cytotoxicity studies showed that the potency of **5** and **6** towards U2OS and U2OS‐MTX cells significantly decreased in the presence of z‐VAD‐FMK (5 μM), a peptide‐based caspase‐dependent apoptosis inhibitor (*p*<0.05, n=18, IC_50_ value of **5**=1.09±0.02 μM and IC_50_ value of **6**=0.23±0.01 μM for U2OS cells; IC_50_ value of **5**=1.09±0.10 μM and IC_50_ value of **6**=0.35±0.05 μM for U2OS‐MTX cells, Figures S64–S65). This further suggests that **5** and **6** induce caspase‐dependent osteosarcoma cell death. Independent studies showed that the inhibitory effect of **5** and **6** (at the IC_20_ values for 10 days) on U2OS‐MTX sarcosphere formation was attenuated, in terms of the size and spherical nature of sarcospheres formed, in the presence of z‐VAD‐FMK (5 μM) (Figure S66). The potency of **5** and **6** towards U2OS‐MTX sarcospheres also significantly decreased in the presence of z‐VAD‐FMK (5 μM) (IC_50_ value for **5**=4.14±0.08 μM, 4.3‐fold, *p* <0.05; IC_50_ value for **6**=5.10±0.11 μM, 5.3‐fold, *p* <0.05) (Figure S67). This is consistent with the monolayer cytotoxicity data in the presence of z‐VAD‐FMK and further supports the notion that **5** and **6** induce caspase‐dependent apoptosis in osteosarcoma cells. Overall the cellular uptake, immunoblotting, and (monolayer and sarcosphere) cytotoxicity studies indicate that the mechanism of action of **5** and **6** is likely to involve osteosarcoma cell and nucleus entry, genomic DNA damage, and caspase‐dependent apoptosis.

### Cyclooxygenase‐2 inhibition in osteosarcoma stem cells

**Figure 3:**
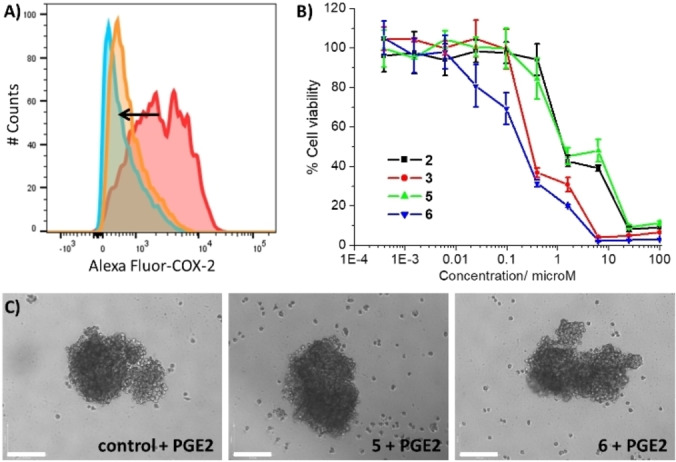
(A) Representative histograms displaying the green fluorescence emitted by anti‐COX‐2 Alexa Fluor 488 nm antibody‐stained U2OS‐MTX cells treated with LPS (2.5 μM) for 24 h, followed by 48 h in fresh media (red) or media containing **5** (IC_50_ value, blue) or **6** (IC_50_ value, orange). (B) Representative dose‐response curves for the treatment of U2OS‐MTX cells with **2**–**3** or **5**–**6** after 72 h incubation in the presence of PGE2 (20 μM). (C) Representative bright‐field images (x10) of U2OS‐MTX sarcospheres treated with PGE2 (20 μM) in the absence and presence of **5** or **6** at their respective IC_20_ values for 10 days. Scale bar=150 μm.

COX‐2 is an established marker in osteosarcoma and its inhibition is widely thought to be a viable method of improving therapeutic outcomes.[^12^, ^19^] Histological studies have shown that COX‐2 expression in osteosarcoma patients correlates to tumour grade, metastasis potential, and lower survival rates.^[12b]^ Notably, COX‐2 expression is elevated in OSCs (compared to bulk osteosarcoma cells) and plays a vital role in OSC maintenance.^[12a]^ Therefore COX‐2 inhibitors could serve as useful tools for targeting OSCs. Global transcriptional analysis of the U2OS cell line revealed that COX‐2 expression in the OSC proportion was 42‐fold higher than in the bulk osteosarcoma cell proportion.^[12a]^ Given the presence of diflunisal, a COX‐2 inhibitor, in **5** and **6**, we investigated whether the mechanism of action of **5** and **6** involved COX‐2 inhibition. U2OS‐MTX cells pre‐treated with lipopolysaccharide (LPS) (2.5 μM for 24 h), to increase basal COX‐2 levels (Figure S68), were treated with **5**, **6**, or diflunisal (various concentrations for 48 h) and the COX‐2 expression was determined by flow cytometry. A dramatic decrease in COX‐2 expression compared to untreated cells was observed for U2OS‐MTX cells treated with **5** (IC_50_ value for 48 h) and **6** (IC_50_ value for 48 h) (Figure 3A). As expected, a decrease in COX‐2 expression was also observed in U2OS‐MTX cells treated with diflunisal (10‐40 μM for 48 h) (Figure S69). Overall, the flow cytometric data suggests that the cytotoxic mechanism of action of **5** and **6** may involve COX‐2 downregulation.

To further prove that **5** and **6** evoke COX‐2‐dependent OSC death, monolayer cytotoxicity studies were performed with U2OS‐MTX cells in the presence and absence of prostaglandin E2 (PGE2) (20 μM), the functional product of COX‐2‐mediated arachidonic acid metabolism. The potency of **5** and **6** towards U2OS‐MTX cells decreased significantly in the presence of PGE2 (IC_50_ value for **5**=1.32±0.04 μM, 3.3‐fold, *p*<0.05; IC_50_ value for **6**=0.20±0.01 μM, 3.3‐fold, *p*<0.05) (Figure 3B), suggesting that **5** and **6** induce OSC death through a COX‐2‐dependent pathway. The potency of the corresponding *cis*‐dichlorobis‐(polypyridyl) gallium(III) chloride complexes **2** and **3** towards U2OS‐MTX cells also decreased in the presence of PGE2 however the change was statistically insignificant (IC_50_ value for **2**=1.29±0.05 μM, *p*=0.052; IC_50_ value for **3**=0.29±0.01 μM, *p*=0.052) (Figure 3B). Taken together, this suggests that the diflunisal moiety within **5** and **6** is likely to be responsible for the COX‐2‐dependent cytotoxicity pathway evoked by **5** and **6**. Additional cytotoxicity studies in three‐dimensional cultures showed that the potency of **5** and **6** towards U2OS‐MTX sarcospheres significantly decreased in the presence PGE2 (20 μM) (IC_50_ value for **5**=3.61±0.04 μM, 3.7‐fold, *p* <0.05; IC_50_ value for **6**=3.70±0.35 μM, 3.8‐fold, *p* <0.05) (Figure S70). Under these conditions, the ability of **5** and **6** (at the IC_20_ values for 10 days) to inhibit sarcosphere formation was also markedly attenuated (Figure 3C). This is consistent with the monolayer cytotoxicity data in the presence of PGE2 and further supports the notion that **5** and **6** induce OSC death via a COX‐2‐dependent pathway.

## Conclusion

In summary we report the synthesis, spectroscopic and analytical characterisation, and OSC activity of a new series of gallium(III)‐polypyridyl complexes containing the non‐steroidal anti‐inflammatory drug, diflunisal (**5**‐**7**). The X‐ray crystal structure of the 3,4,7,8‐tetramethyl‐1,10‐phenanthroline‐containing complex **6** indicated that the gallium(III) complex adopts a six‐coordinate distorted octahedral geometry. The stability of the gallium(III) complexes in biologically relevant solutions is dependent on the polypyridyl ligand present. The 4,7‐diphenyl‐1,10‐phenanthroline‐bearing complex **7** was unstable whereas the 5‐methyl‐1,10‐phenanthroline‐ and 3,4,7,8‐tetramethyl‐1,10‐phenanthroline‐containing complexes **5** and **6** respectively, were deemed to be suitably stable in biological solutions (including under reducing conditions) to progress to cell‐based studies.

Cytotoxicity studies revealed that **5** and **6** equipotently kill bulk osteosarcoma cells and OSCs in the submicro‐ to nano‐molar range. Therefore **5** and **6** have the potential to remove heterogenous osteosarcoma populations (containing bulk osteosarcoma cells and OSCs) with a single submicro‐ or nano‐molar dose. The diflunisal moiety is a contributing factor in the OSC potency of **5** and **6**, and this was shown by the fact that the corresponding chloride‐containing complexes without diflunisal **2** and **3** were significantly less toxic towards OSCs than **5** and **6**. Strikingly, **6** displayed up to three orders of magnitude higher potency towards OSCs in monolayer cultures than clinically approved osteosarcoma drugs (cisplatin, doxorubicin, etoposide, ifosfamide, and carboplatin) and the well‐established anti‐CSC drug candidate, salinomycin. Furthermore, **5** and **6** reduced sarcosphere size and viability (in the submicro‐molar range) to a better extent than cisplatin, carboplatin, and salinomycin.

Mechanistic studies indicated that **5** and **6** reduced the overall expression of COX‐2 in OSCs and induced OSC death through a COX‐2‐dependent pathway. Additionally, the complexes entered osteosarcoma cell nuclei, activated the DNA damage response pathway, and evoked caspase‐dependent apoptosis. As far as we are aware, this is the first report of a metal complex to induce osteosarcoma cell death via COX‐2‐inhibition and DNA damage. Our findings reinforce the therapeutic potential of gallium(III) coordination complexes and could pave the way for the development of other DNA‐damaging, COX‐inhibiting metal complexes for OSC‐directed chemotherapy.

## Experimental Section

### Materials and methods

All synthetic procedures were performed under normal atmospheric conditions or under nitrogen. Fourier transform infrared (FTIR) spectra were recorded with an IRAffinity‐1S Shimadzu spectrophotometer. Electron spray ionisation mass spectra were recorded on a Micromass Quattro spectrometer. UV‐vis absorption spectra were recorded on a Cary 3500 UV‐Vis spectrophotometer. ^1^H, ^13^C, ^19^F, and ^31^P NMR spectra were recorded on a BrukerAvance 400 MHz Ultrashield NMR spectrometer. ^1^H NMR spectra were referenced internally to residual solvent peaks, and chemical shifts are expressed relative to tetramethylsilane, SiMe_4_ (δ=0 ppm). Elemental analysis of the compounds prepared was performed commercially by the University of Cambridge. Diflunisal, 5‐methyl‐1,10‐phenanthroline, 3,4,7,8‐tetramethyl‐1,10‐phenanthroline, 4,7‐diphenyl‐1,10‐phenanthroline were purchased from Sigma Aldrich or Alfa Aesar and used as received.

**Synthesis of [Ga^III^(5‐methyl‐1,10‐phenanthroline)_2_Cl_2_]Cl, 2**: A solution of 5‐methyl‐1,10‐phenanthroline (554.0 mg, 2.85 mmol) in dry DCM (8 mL) was added to a solution of GaCl_3_ (251.0 mg, 1.43 mmol) in dry DCM (10 mL). The reaction mixture was stirred for 2 h at ambient temperature under N_2_ and then diethyl ether (20 mL) was added. The resulting precipitate was collected by centrifugation and dried under vacuum to yield **2** as a white solid (635 mg, 79 %); ^1^H NMR (400 MHz, MeOH‐d_4_): δ_H_ 10.22 (t, 1H), 10.11 (t, 1H), 9.37 (d, 1H), 9.14 (d, 1H), 8.89 (dd, 1H), 8.66 (dd, 1H), 8.57‐8.53 (m, 1H), 8.49–8.45 (m, 1H), 8.26 (s, 1H), 8.13 (s, 1H), 7.75‐7.70 (m, 2H), 7.66–7.61 (m, 2H), 3.00 (s, 3H), 2.87 (s, 3H); ^13^C NMR (101 MHz, MeOH‐d_4_): δ_C_ 149.81, 149.77, 149.35, 149.31, 145.58, 145.08, 143.37, 143.35, 141.50, 141.35, 141.33, 139.43, 138.37, 137.78, 137.53, 137.40, 136.55, 131.76, 131.53, 131.47, 131.25, 127.86, 127.63, 127.39, 19.21, 19.06; IR (solid, ATR, cm^−1^): 3365, 3049, 1626, 1611, 1586, 1526, 1491, 1431, 1395, 888, 833, 813, 728, 652, 432; ESI‐MS Calcd. for C_26_H_20_N_4_Cl_2_Ga [M−Cl]^+^: 529.0305 a.m.u. Found [M−Cl]^+^: 529.0303 a.m.u.; Anal. Calcd. for C_26_H_20_N_4_Cl_3_Ga ⋅ 1.5H_2_O (%): C, 52.79; H, 3.92; N, 9.47. Found: C, 52.49; H, 3.59; N, 9.28.

**Synthesis of [Ga^III^(3,4,7,8‐tetramethyl‐1,10‐phenanthroline)_2_Cl_2_]Cl, 3**: A solution of 3,4,7,8‐tetramethyl‐1,10‐phenanthroline (474.0 mg, 2.01 mmol) in dry DCM (15 mL) was added to a solution of GaCl_3_ (176.0 mg, 1.00 mmol) in dry DCM (8 mL). The reaction mixture was stirred for 2 h at ambient temperature under N_2_ and then diethyl ether (30 mL) was added. The resulting precipitate was collected by centrifugation and dried under vacuum to yield **3** as a white solid (616 mg, 95 %); ^1^H NMR (400 MHz, MeOH‐d_4_): δ_H_ 9.92 (s, 2H), 8.61 (d, 2H), 8.49 (d, 2H), 7.45 (s, 2H), 3.09 (s, 6H), 2.89 (s, 6H), 2.74 (s, 6H), 2.20 (s, 6H); ^13^C NMR (101 MHz, MeOH‐d_4_): δ_C_ 151.42, 149.42, 148.79, 144.41, 135.65, 135.48, 135.04, 134.90, 128.17, 127.94, 123.77, 123.72, 16.97, 16.06, 14.35, 13.78; IR (solid, ATR, cm^−1^): 2913, 1617, 1603, 1530, 1433, 1389, 1312, 1248, 1176, 1015, 894, 875, 841, 816, 739, 724, 641, 622, 573, 554, 525, 471; ESI‐MS Calcd. for C_32_H_32_N_4_Cl_2_Ga [M−Cl]^+^: 613.1245 a.m.u. Found [M−Cl]^+^: 613.1260 a.m.u; Anal. Calcd. for C_32_H_32_N_4_Cl_3_Ga ⋅ 2.5H_2_O (%): C, 55.40; H, 5.38; N, 8.08. Found: C, 55.80; H, 5.65; N, 8.21.

**Synthesis of [Ga^III^(4,7‐diphenyl‐1,10‐phenanthroline)_2_Cl_2_]Cl, 4**: A solution of 4,7‐diphenyl‐1,10‐phenanthroline (496.6 mg, 1.49 mmol) in dry DCM (15 mL) was added to a solution of GaCl_3_ (131.0 mg, 0.74 mmol) in dry DCM (8 mL). The reaction mixture was stirred for 2 h at ambient temperature under N_2_, concentrated to ∼2 mL, and then diethyl ether (20 mL) was added. The resulting precipitate was collected by centrifugation and dried under vacuum to yield **4** as a white solid (550 mg, 88 %); ^1^H NMR (400 MHz, MeOH‐d_4_): δ_H_ 10.32 (d, 2H), 8.52 (d, 2H), 8.42 (d, 2H), 8.32‐8.27 (m, 2H), 8.05 (d, 2H), 7.91 (d, 4H), 7.78‐7.70 (m, 8H), 7.61‐7.55 (m, 10H); ^13^C NMR (101 MHz, MeOH‐d_4_): δ_C_ 155.48, 153.71, 148.56, 144.52, 137.84, 136.94, 135.53, 135.26, 130.12, 129.80, 129.74, 129.37, 129.08, 128.89, 127.88, 127.60, 126.70, 126.40, 125.67, 125.56; IR (solid, ATR, cm^‐1^): 3054, 1624, 1607, 1559, 1525, 1495, 1426, 1400, 1233, 1095, 850, 812, 765, 739, 700, 670, 631, 597, 576, 550, 485; ESI‐MS Calcd. for C_48_H_32_N_4_Cl_2_Ga [M−Cl]^+^: 805.1249 a.m.u. Found [M−Cl]^+^: 805.1224 a.m.u.; Anal. Calcd. for C_48_H_32_N_4_Cl_3_Ga ⋅ 1.5CH_2_Cl_2_ (%): C, 61.40; H, 3.64; N, 5.79. Found: C, 61.53; H, 3.78; N, 5.88 (presence of DCM is evidenced in the X‐ray crystal structure of **4**).

**Synthesis of [Ga^III^(5‐methyl‐1,10‐phenanthroline)_2_(diflunisal)](PF_6_), 5**: A solution of diflunisal (55.2 mg, 0.22 mmol) and Cs_2_CO_3_ (72.2 mg, 0.22 mmol) in MeOH (8 mL) was added to a solution of **2** (123.6 mg, 0.22 mmol) in MeOH (15 mL). The reaction mixture was stirred at ambient temperature under an aerobic atmosphere for 20 h and then refluxed at 60 °C for 2 h. The solvent was then removed and the resultant solid was dissolved in MeOH (10 mL) and stirred with KPF_6_ (340.0 mg, 1.85 mmol) in acetone (5 mL) for 10 min. Upon addition of distilled water (40 mL) a precipitate formed. The precipitate was collected by centrifugation and washed with H_2_O (30 mL ×2) and Et_2_O (20 mL). The resultant solid was dissolved in DCM (20 mL) and dried by Na_2_SO_4_ (5 g) for 20 min. Upon removal of Na_2_SO_4_ by filtration and DCM under vacuum, **5** was isolated as a white solid (170 mg, 91 %); ^1^H NMR (400 MHz, MeOH‐d_4_): δ_H_ 9.49 (dt, 1H), 9.40 (dt, 1H), 9.36‐9.32 (m, 1H), 9.13‐9.09 (m, 1H), 9.05 (ddd, 1H), 8.82 (ddd, 1H), 8.50‐8.41 (m, 1H), 8.41–8.32 (m, 1H), 8.28 (d, 1H), 8.20 (d, 1H), 8.10‐8.09 (m, 1H), 8.02‐8.00 (m, 1H), 7.94‐7.77 (m, 3H), 7.46–7.41 (m, 2H), 7.00 (t, 2H), 6.74 (dd, 1H), 3.03 (d, 3H), 2.95 (d, 3H); ^19^F NMR (376 MHz, MeOH‐d_4_): δ_F_ −74.80 (d, 6F), −114.65 (d, 1F), −115.62 (d, 1F); ^31^P NMR (162 MHz, MeOH‐d_4_): δ_P_ −144.67 (quint, 1P); IR (solid, ATR, cm^‐1^): 1633, 1610, 1589, 1528, 1475, 1430, 1405, 1315, 1278, 1229, 1143, 967, 906, 836, 730, 664, 558, 427; ESI‐MS Calcd. for C_39_H_26_N_4_O_3_F_2_Ga [M‐PF_6_]^+^: 705.1229 a.m.u. Found [M‐PF_6_]^+^: 705.1223 a.m.u.; Anal. Calcd. for C_39_H_26_N_4_O_3_F_8_PGa ⋅ 2H_2_O (%): C, 52.79; H, 3.41; N, 6.31. Found: C, 52.79; H, 3.05; N, 6.18.

**Synthesis of [Ga^III^(3,4,7,8‐tetramethyl‐1,10‐phenanthroline)_2_(diflunisal)](PF_6_), 6**: A solution of diflunisal (48.4 mg, 0.19 mmol) and Cs_2_CO_3_ (63.5 mg, 0.19 mmol) in MeOH (8 mL) was added to a solution of **3** (125.6 mg, 0.19 mmol) in MeOH (20 mL). The reaction mixture was stirred at ambient temperature under an aerobic atmosphere for 20 h and then refluxed at 60 °C for 2 h. The solvent was then removed and the resultant solid was dissolved in MeOH (10 mL) and stirred with KPF_6_ (320.0 mg, 1.74 mmol) in acetone (5 mL) for 10 min. Upon addition of distilled water (40 mL) a precipitate formed. The precipitate was collected by centrifugation and washed with H_2_O (30 mL×2) and Et_2_O (20 mL). The resultant solid was dissolved in DCM (20 mL) and dried by Na_2_SO_4_ (5 g) for 20 min. Upon removal of Na_2_SO_4_ by filtration and DCM under vacuum, **6** was isolated as a white solid (140 mg, 77 %); ^1^H NMR (400 MHz, MeOH‐d_4_): δ_H_ 9.20 (d, 1H), 9.11 (d, 1H), 8.62‐8.49 (m, 4H), 8.07 (d, 1H), 7.79 (s, 1H), 7.68 (s, 1H), 7.47‐7.41 (m, 2H), 7.00 (t, 2H), 6.72 (d, 1H), 3.02 (s, 3H), 3.00 (s, 3H), 2.83 (s, 3H), 2.80 (s, 3H), 2.77 (s, 3H), 2.66 (s, 3H), 2.33 (s, 3H), 2.30 (s, 3H); ^19^F NMR (376 MHz, MeOH‐d_4_): δ_F_ −74.92 (d, 6F), −114.67 (d, 1F), −115.62 (d, 1F); ^31^P NMR (162 MHz, MeOH‐d_4_): δ_P_ −144.70 (quint, 1P); IR (solid, ATR, cm^‐1^): 1631, 1600, 1538, 1472, 1430, 1406, 1391, 1335, 1306, 1263, 1254, 1140, 1112, 1022, 965, 898, 875, 837, 747, 723, 643, 624, 590, 557, 472, 439; ESI‐MS Calcd. for C_45_H_38_N_4_O_3_F_2_Ga [M‐PF_6_]^+^: 789.2168 a.m.u. Found [M‐PF_6_]^+^: 789.2155 a.m.u.; Anal. Calcd. for C_45_H_38_N_4_O_3_F_8_PGa ⋅ 2H_2_O (%): C, 55.63; H, 4.36; N, 5.77. Found: C, 55.84; H, 3.98; N, 5.76.

**Synthesis of [Ga^III^(4,7‐diphenyl‐1,10‐phenanthroline)_2_(diflunisal)](PF_6_), 7**: A solution of diflunisal (28.0 mg, 0.11 mmol) in dry DCM (8 mL) was added to a solution of **4** (94.5 mg, 0.11 mmol) in dry DCM (5 mL). Then Na_2_CO_3_ (10.0 mg, 0.09 mmol) was added. The reaction mixture was stirred at ambient temperature under an aerobic atmosphere for 4 days. The solvent was then removed and the resultant solid was dissolved in MeOH (10 mL) and stirred with KPF_6_ (196.0 mg, 1.06 mmol) in acetone (5 mL) for 10 min. Upon addition of distilled water (40 mL) a precipitate formed. The precipitate was collected by centrifugation and washed with H_2_O (30 mL×2) and Et_2_O (20 mL). The resultant solid was dissolved in DCM (20 mL) and dried by Na_2_SO_4_ (5 g) for 20 min. Upon removal of Na_2_SO_4_ by filtration and DCM under vacuum, **7** was isolated as a white solid (110 mg, 87 %); ^1^H NMR (400 MHz, MeOH‐d_4_): δ_H_ 9.62 (d, 1H), 9.56 (d, 1H), 8.45–8.42 (m, 2H), 8.41–8.37 (m, 2H), 8.34‐8.32 (m, 2H), 8.25 (d, 1H), 8.17‐8.16 (m, 1H), 7.90 (d, 1H), 7.87–7.84 (m, 4H), 7.75–7.70 (m, 6H), 7.65–7.62 (m, 10H), 7.51‐7.43 (m, 3H), 7.01 (t, 3H), 6.84 (d, 1H); ^19^F NMR (376 MHz, MeOH‐d_4_): δ_F_ −74.89 (d, 6F), −114.63 (d, 1F), −115.59 (d, 1F); ^31^P NMR (162 MHz, MeOH‐d_4_) δ_P_ −144.68 (quint, 1P); IR (solid, ATR, cm^‐1^): 1628, 1609, 1562, 1526, 1474, 1427, 1403, 1320, 1280, 1232, 1141, 967, 836, 768, 741, 701, 634, 554; ESI‐MS Calcd. for C_61_H_38_N_4_O_3_F_2_Ga [M‐PF_6_]^+^: 981.2168 a.m.u. Found [M‐PF_6_]^+^: 981.2153 a.m.u.; Anal. Calcd. for C_61_H_38_N_4_O_3_F_8_PGa ⋅ 2H_2_O (%): C, 62.96; H, 3.64; N, 4.81. Found: C, 62.69; H, 3.40; N, 4.71.

### X‐ray single crystal diffraction analysis

Single crystals of complex **4** were obtained by slow diffusion of diethyl ether into a concentrated methanol‐dichloromethane solution of **4**. Single crystals of complex **6** were obtained by slow diffusion of diethyl ether into an acetonitrile solution of **6**. Crystals suitable for X‐ray diffraction analysis were selected and mounted on a Bruker Apex 2000 CCD area detector diffractometer using standard procedures. Data was collected using graphite‐monochromated Mo−Kα radiation (λ=0.71073) at 150(2) K. Crystal structures were solved and refined using the Bruker SHELXTL software.^[20]^ All hydrogen atoms were located by geometrical calculations, and all non‐hydrogen atoms were refined anisotropically. Cambridge Crystallographic Data Centre Deposition Number(s) 2086778 (for **4**), 2086777 (for **6**) contain(s) the supplementary crystallographic data for this paper. These data are provided free of charge by the joint Cambridge Crystallographic Data Centre and Fachinformationszentrum Karlsruhe http://www.ccdc.cam.ac.uk/structures, Access Structures service.

### Measurement of water‐octanol partition coefficient (LogP)

The LogP values for **5**–**7** were determined using the shake‐flask method and UV‐vis spectroscopy. The 1‐octanol used in this experiment was pre‐saturated with water. An aqueous solution of **5**–**7** (500 μL, 100 μM) was incubated with 1‐octanol (500 μL) in a 1.5 mL tube. The tube was shaken at room temperature for 2 h. The two phases were separated by centrifugation and the **5**–**7** content in each phase was determined by UV‐vis spectroscopy.

### Cell lines and cell culture conditions

The U2OS bone osteosarcoma cell line was acquired from American Type Culture Collection (ATCC, Manassas, VA, USA) and cultured in Dulbecco's Modified Eagle's Medium (DMEM) supplemented with 10 % fetal bovine serum and 1 % penicillin. The cells were grown at 310 K in a humidified atmosphere containing 5 % CO_2_. To gain access to OSC‐enriched cells, a full T75 flask of U2OS cells was treated with MTX (300 nM) for 4 days. The cells (labelled U2OS‐MTX cells) were then used immediately.

### Cytotoxicity MTT assay

The colourimetric MTT assay was used to determine the toxicity of **2**–**3**, **5**–**6**, diflunisal, doxorubicin, etoposide, and ifosfamide. U2OS and U2OS‐MTX cells (5×10^3^) were seeded in each well of a 96‐well plate. After incubating the cells overnight, various concentrations of the compounds (0.0004–100 μM) were added and incubated for 72 h (total volume 200 μL). Stock solutions of the compounds were prepared as 5 or 10 mM solutions in DMSO or PBS and diluted using media. For compounds made up in DMSO, the final concentration of DMSO in each well was 0.5 % and this amount was present in the untreated control as well. After 72 h, 20 μL of a 4 mg/mL solution of MTT in PBS was added to each well, and the plate was incubated for an additional 4 h. The DMEM/MTT mixture was aspirated and 200 μL of DMSO was added to dissolve the resulting purple formazan crystals. The absorbance of the solutions in each well was read at 550 nm. Absorbance values were normalized to (DMSO‐containing) control wells and plotted as concentration of test compound versus % cell viability. IC_50_ values were interpolated from the resulting dose dependent curves. The reported IC_50_ values are the average of three independent experiments, each consisting of six replicates per concentration level (overall n=18).

### Sarcosphere formation and viability assay

U2OS‐MTX cells (5×10^3^) were plated in ultralow‐attachment 96‐well plates (Corning) and incubated in DMEM supplemented with N2 (Invitrogen), human EGF (10 ng/mL), and human bFGF (10 ng/mL) for 10 days. Studies were also conducted in the presence of **2**–**3**, **5**–**6**, diflunisal, cisplatin, carboplatin, and salinomycin (0–133 μM). Sarcospheres treated with **2**–**3**, **5**–**6**, diflunisal, cisplatin, carboplatin, and salinomycin (at their respective IC_20_ values, 10 days) were imaged using an inverted microscope. The viability of the sarcospheres was determined by addition of a resazurin‐based reagent, TOX8 (Sigma). After incubation for 16 h, the fluorescence of the solutions was read at 590 nm (λ_ex_=560 nm). Viable sarcospheres reduce the amount of the oxidized TOX8 form (blue) and concurrently increases the amount of the fluorescent TOX8 intermediate (red), indicating the degree of sarcosphere cytotoxicity caused by the test compound. Fluorescence values were normalized to DMSO‐containing controls and plotted as concentration of test compound versus % sarcosphere viability. IC_50_ values were interpolated from the resulting dose dependent curves. The reported IC_50_ values are the average of three independent experiments, each consisting of three replicates per concentration level (overall n=9).

### Cellular uptake

To measure the cellular uptake of **2**–**3** and **5**–**6**, ca. 1 million U2OS cells were treated with **2**–**3** and **5**–**6** (0.25 μM) at 37 °C for 24 h. After incubation, the media was removed and the cells were washed with PBS (2 mL×3), and harvested. The number of cells was counted at this stage, using a haemocytometer. This mitigates any cell death induced by **2**–**3** and **5**–**6** at the administered concentration and experimental cell loss. The cells were centrifuged to form pellets. The cellular pellets were dissolved in 65 % HNO_3_ (250 μL) overnight. The cellular pellets were also used to determine the gallium content in the nuclear fraction. The Thermo Scientific NE‐PER Nuclear and Cytoplasmic Extraction Kit was used to extract and separate the nuclear fraction. The fractions were dissolved in 65 % HNO_3_ overnight (250 μL final volume). All samples were diluted 5‐fold with water and analysed using inductively coupled plasma mass spectrometry (ICP‐MS, Thermo Scientific iCAP‐Qc quadrupole). Gallium levels are expressed as Ga (ng) per million cells. Results are presented as the mean of four determinations for each data point.

### Immunoblotting analysis

U2OS cells (5×10^5^ cells) were incubated with **5** (0.18−0.70 μM for 24 h or 72 h) and **6** (22−66 nM for 24 h or 72 h) at 37 °C. Cells were washed with PBS, scraped into SDS‐PAGE loading buffer (64 mM Tris‐HCl (pH 6.8)/ 9.6 % glycerol/ 2 %SDS/ 5 % β‐mercaptoethanol/ 0.01 % Bromophenol Blue), and incubated at 95 °C for 10 min. Whole cell lysates were resolved by 4–20 % sodium dodecylsulphate polyacylamide gel electrophoresis (SDS‐PAGE; 200 V for 25 min) followed by electro transfer to polyvinylidene difluoride membrane, PVDF (350 mA for 1 h). Membranes were blocked in 5 % (w/v) non‐fat milk in PBST (PBS/0.1 % Tween 20) and incubated with the appropriate primary antibodies (Cell Signalling Technology). After incubation with horseradish peroxidase‐conjugated secondary antibodies (Cell Signalling Technology), immune complexes were detected with the ECL detection reagent (Bio‐Rad) and analysed using a chemiluminescence imager (Bio‐Rad ChemiDoc Imaging System).

### Flow cytometry

U2OS‐MTX cells were seeded in 6‐well plates (at a density of 5×10^5^ cells/mL) and the cells were allowed to attach overnight. The cells were treated with lipopolysaccharide (LPS) (2.5 μg/L for 24 h), and then treated with **5** (IC_50_ value), **6** (IC_50_ value) or diflunisal (10–40 μM) and incubated for a further 48 h. The cells were then harvested by trypsinization, fixed with 4 % paraformaldehyde (at 37 °C for 10 min), permeabilised with ice‐cold methanol (for 30 min), and suspended in PBS (200 μL). The Alexa Fluor® 488 nm labelled anti‐COX‐2 antibody (5 μL) was then added to the cell suspension and incubated in the dark for 1 h. The cells were then washed with PBS (1 mL) and analysed using a FACSCanto II flow cytometer (BD Biosciences) (10,000 events per sample were acquired) at the University of Leicester FACS Facility. The FL1 channel was used to assess COX‐2 expression. Cell populations were analysed using the FlowJo software (Tree Star).

## Conflict of interest

The authors declare no conflict of interest.

## Supporting information

As a service to our authors and readers, this journal provides supporting information supplied by the authors. Such materials are peer reviewed and may be re‐organized for online delivery, but are not copy‐edited or typeset. Technical support issues arising from supporting information (other than missing files) should be addressed to the authors.

Supporting Information
