# Supplementary material for: Osteosarcoma Stem Cell Potent Gallium(III)‐Polypyridyl Complexes Bearing Diflunisal
Source: Chemistry. 2021 Aug 20;27(55):13846–54. doi: 10.1002/chem.202102207 (PMC8518360; doi:10.1002/chem.202102207)
Supplement: Supplementary file 1 — Supporting Information [file CHEM-27-13846-s001.pdf]

Chemistry–A European Journal

Supporting Information

Osteosarcoma Stem Cell Potent Gallium(III)-Polypyridyl Complexes Bearing Diflunisal

Zhiyin Xiao[+], Ginevra Passeri[+], Joshua Northcote-Smith[+], Kuldip Singh, and Kogularamanan Suntharalingam*

<u>**Table of Content**</u>

**Figure S1.**	^{1}H NMR spectrum of **2** in MeOH-d_4.
**Figure S2.**	^{13}C NMR spectrum of **2** in MeOH-d_4.
**Figure S3.**	^{1}H NMR spectrum of **3** in MeOH-d_4.
**Figure S4.**	^{13}C NMR spectrum of **3** in MeOH-d_4.
**Figure S5.**	^{1}H NMR spectrum of **4** in MeOH-d_4.
**Figure S6.**	^{13}C NMR spectrum of **4** in MeOH-d_4.
**Figure S7.**	^{1}H NMR spectrum of 5-methyl-1,10-phenanthroline in MeOH-d_4.
**Figure S8.**	ESI mass spectrum (positive mode) of **2**.
**Figure S9.**	ESI mass spectrum (positive mode) of **3**.
**Figure S10.**	ESI mass spectrum (positive mode) of **4**.
**Figure S11.**	^{1}H NMR spectrum of 3,4,7,8-tetramethyl-1,10-phenanthroline in MeOH-d_4.
**Figure S12.**	^{1}H NMR spectrum of 4,7-diphenyl-1,10-phenanthroline in MeOH-d_4.
**Figure S13.**	IR spectrum of (A) **2**, (B) **3**, and (C) **4** in the solid form.
**Table S1.**	Crystallographic data for complex **4**.
**Table S2.**	Selected bond lengths (Å) and angles (°) for complex **4**.
**Figure S14.**	IR spectrum of (A) **5**, (B) **6**, (C) **7**, and (D) diflunisal in the solid form.
**Figure S15.**	^{1}H NMR spectrum of **5** in MeOH-d_4.
**Figure S16.**	$^{19}F\{^{1}H\}$ NMR spectrum of **5** in MeOH-d_4.
**Figure S17.**	$^{31}P\{^{1}H\}$ NMR spectrum of **5** in MeOH-d_4.
**Figure S18.**	^{1}H NMR spectrum of **6** in MeOH-d_4.
**Figure S19.**	$^{19}F\{^{1}H\}$ NMR spectrum of **6** in MeOH-d_4.
**Figure S20.**	$^{31}P\{^{1}H\}$ NMR spectrum of **6** in MeOH-d_4.
**Figure S21.**	^{1}H NMR spectrum of **7** in MeOH-d_4.
**Figure S22.**	$^{19}F\{^{1}H\}$ NMR spectrum of **7** in MeOH-d_4.
**Figure S23.**	$^{31}P\{^{1}H\}$ NMR spectrum of **7** in MeOH-d_4.
**Figure S24.**	ESI mass spectrum (positive mode) of **5**.
**Figure S25.**	ESI mass spectrum (positive mode) of **6**.
**Figure S26.**	ESI mass spectrum (positive mode) of **7**.
**Figure S27.**	^{1}H NMR spectrum of diflunisal in MeOH-d_4.
**Figure S28.**	$^{19}F\{^{1}H\}$ NMR spectrum of diflunisal in MeOH-d_4.
**Table S3.**	Crystallographic data for complex **6**.
**Table S4.**	Selected bond lengths (Å) and angles (°) for complex **6**.
**Table S5.**	Experimentally determined LogP values for **5**-**7**.
**Figure S29.**	UV-Vis spectrum of **5** (25 μM) in DMSO over the course of 24 h at 37 °C.
**Figure S30.**	UV-Vis spectrum of **6** (25 μM) in DMSO over the course of 24 h at 37 °C.
**Figure S31.**	UV-Vis spectrum of **7** (25 μM) in DMSO over the course of 24 h at 37 °C.
**Figure S32.**	UV-Vis spectrum of **5** (25 μM) in H_2O:DMSO (200:1) over the course of 24 h at 37 °C.
**Figure S33.**	UV-Vis spectrum of **6** (25 μM) in H_2O:DMSO (200:1) over the course of 24 h at 37 °C.
**Figure S34.**	UV-Vis spectrum of **7** (25 μM) in H_2O:DMSO (200:1) over the course of 24 h at 37 °C.
**Figure S35.**	UV-Vis spectrum of **5** (25 μM) in PBS:DMSO (200:1) over the course of 24 h at 37 °C.

Figure S36. UV-Vis spectrum of **6** (25 μM) in PBS:DMSO (200:1) over the course of 24 h at 37 ^oC.
Figure S37. UV-Vis spectrum of **7** (25 μM) in PBS:DMSO (200:1) over the course of 24 h at 37 ^oC.
Figure S38. UV-Vis spectra of diflunisal, 5-methyl-1,10-phenanthroline, 3,4,7,8-tetramethyl-1,10-phenanthroline, and 4,7-diphenyl-1,10-phenanthroline (25 μM) in PBS:DMSO (200:1) at 37 ^oC.
Figure S39. UV-Vis spectrum of **5** (25 μM) in the presence of ascorbic acid (250 μM) in PBS:DMSO (200:1) before and after incubation for 24 h at 37 ^oC.
Figure S40. UV-Vis spectrum of **6** (25 μM) in the presence of ascorbic acid (250 μM) in PBS:DMSO (200:1) before and after incubation for 24 h at 37 ^oC.
Figure S41. UV-Vis spectrum of **5** (25 μM) in the presence of glutathione (250 μM) in PBS:DMSO (200:1) before and after incubation for 24 h at 37 ^oC.
Figure S42. UV-Vis spectrum of **6** (25 μM) in the presence of glutathione (250 μM) in PBS:DMSO (200:1) before and after incubation for 24 h at 37 ^oC.
Figure S43. ESI mass spectra (positive mode) of **5** (500 μM) in H_2O:DMSO (10:1) in the presence of ascorbic acid (5 mM) (A) before and (B) after incubation for 24 h at 37 ^oC.
Figure S44. ESI mass spectra (positive mode) of **6** (500 μM) in H_2O:DMSO (10:1) in the presence of ascorbic acid (5 mM) (A) before and (B) after incubation for 24 h at 37 ^oC.
Figure S45. ESI mass spectra (positive mode) of **5** (500 μM) in H_2O:DMSO (10:1) in the presence of glutathione (5 mM) (A) before and (B) after incubation for 24 h at 37 ^oC.
Figure S46. ESI mass spectra (positive mode) of **6** (500 μM) in H_2O:DMSO (10:1) in the presence of glutathione (5 mM) (A) before and (B) after incubation for 24 h at 37 ^oC.
Figure S47. UV-Vis spectrum of **5** (25 μM) in sodium acetate buffer solution (pH 5.2):DMSO (200:1) over the course of 24 h at 37 ^oC.
Figure S48. UV-Vis spectrum of **6** (25 μM) in sodium acetate buffer solution (pH 5.2):DMSO (200:1) over the course of 24 h at 37 ^oC.
Figure S49. UV-Vis spectrum of **5** (25 μM) in DMEM:DMSO (200:1) over the course of 24 h at 37 ^oC.
Figure S50. UV-Vis spectrum of **6** (25 μM) in DMEM:DMSO (200:1) over the course of 24 h at 37 ^oC.
Figure S51. Representative dose-response curves for the treatment of U2OS or U2OS-MTX cells with **5** after 72 h incubation.
Figure S52. Representative dose-response curves for the treatment of U2OS or U2OS-MTX cells with **6** after 72 h incubation.
Figure S53. Representative dose-response curves for the treatment of U2OS or U2OS-MTX cells with doxorubicin after 72 h incubation.
Figure S54. Representative dose-response curves for the treatment of U2OS or U2OS-MTX cells with etoposide after 72 h incubation.
Figure S55. Representative dose-response curves for the treatment of U2OS or U2OS-MTX cells with ifosfamide after 72 h incubation.

**Figure S56.**	Representative dose-response curves for the treatment of U2OS or U2OS-MTX cells with **2** after 72 h incubation.
**Figure S57.**	Representative dose-response curves for the treatment of U2OS or U2OS-MTX cells with **3** after 72 h incubation.
**Figure S58.**	Representative dose-response curves for the treatment of U2OS or U2OS-MTX cells with diflunisal after 72 h incubation.
**Figure S59.**	Representative bright-field images (× 10) of U2OS-MTX sarcospheres in the absence and presence of salinomycin, cisplatin, carboplatin at its IC_{20} value (10 days incubation).
**Table S6.**	IC_{50} values of the gallium complexes, **2-3** and **5-6**, diflunisal, cisplatin, carboplatin, and salinomycin against U2OS-MTX sarcospheres.
**Figure S60.**	Immunoblotting analysis of proteins related to the DNA damage and apoptosis pathways. Protein expression in U2OS cells following treatment with **5** (0.18, 0.35, and 0.7 µM for 72 h).
**Figure S61.**	Immunoblotting analysis of proteins related to the DNA damage and apoptosis pathways. Protein expression in U2OS cells following treatment with **6** (22, 44, and 66 nM for 72 h).
**Figure S62.**	Immunoblotting analysis of proteins related to the DNA damage pathway. Protein expression in U2OS cells following treatment with **5** (0.18, 0.35, and 0.7 µM for 24 h).
**Figure S63.**	Immunoblotting analysis of proteins related to the DNA damage pathway. Protein expression in U2OS cells following treatment with **6** (22, 44, and 66 nM for 24 h).
**Figure S64.**	Representative dose-response curves for the treatment of U2OS cells with **5** or **6** after 72 h incubation in the presence of z-VAD-FMK (5 µM).
**Figure S65.**	Representative dose-response curves for the treatment of U2OS-MTX cells with **5** or **6** after 72 h incubation in the presence of z-VAD-FMK (5 µM).
**Figure S66.**	Representative bright-field images (x 10) of U2OS-MTX sarcospheres treated with z-VAD-FMK (5 µM) in the absence and presence of **5** or **6** at their respective IC_{20} values for 10 days.
**Figure S67.**	Representative dose-response curves for the treatment of U2OS-MTX sarcospheres with **5** or **6** in the presence of z-VAD-FMK (5 µM) after 10 days incubation.
**Figure S68.**	Representative histograms displaying the green fluorescence emitted by anti-COX-2 Alexa Fluor 488 nm antibody-stained U2OS-MTX cells untreated (red) and treated (blue) with LPS (2.5 µM) for 24 h, followed by 48 h in fresh media.
**Figure S69.**	Representative histograms displaying the green fluorescence emitted by anti-COX-2 Alexa Fluor 488 nm antibody-stained U2OS-MTX cells treated with LPS (2.5 µM) for 24 h, followed by 48 h in fresh media (red) or media containing diflunisal (10µM, blue; 20 µM, orange; 40 µM, green).
**Figure S70.**	Representative dose-response curves for the treatment of U2OS-MTX sarcospheres with **5** or **6** in the presence of PGE2 (20 µM) after 10 days incubation.

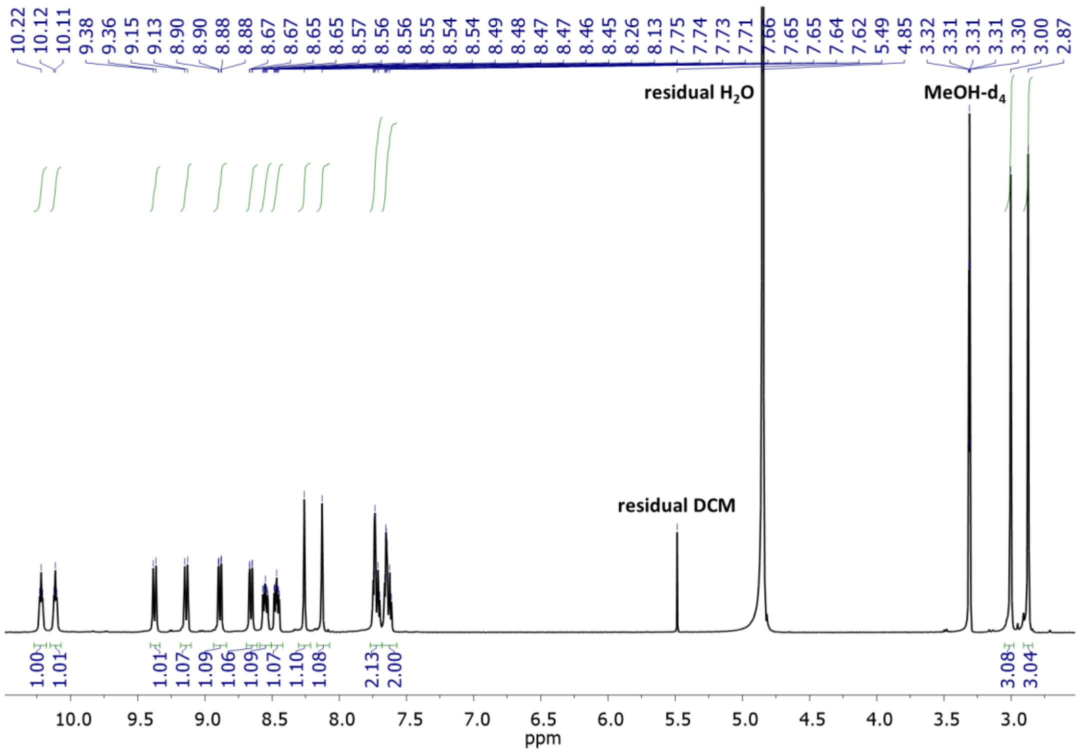

Figure S1. ^{1}H NMR spectrum of **2** in MeOH-d$_4$.

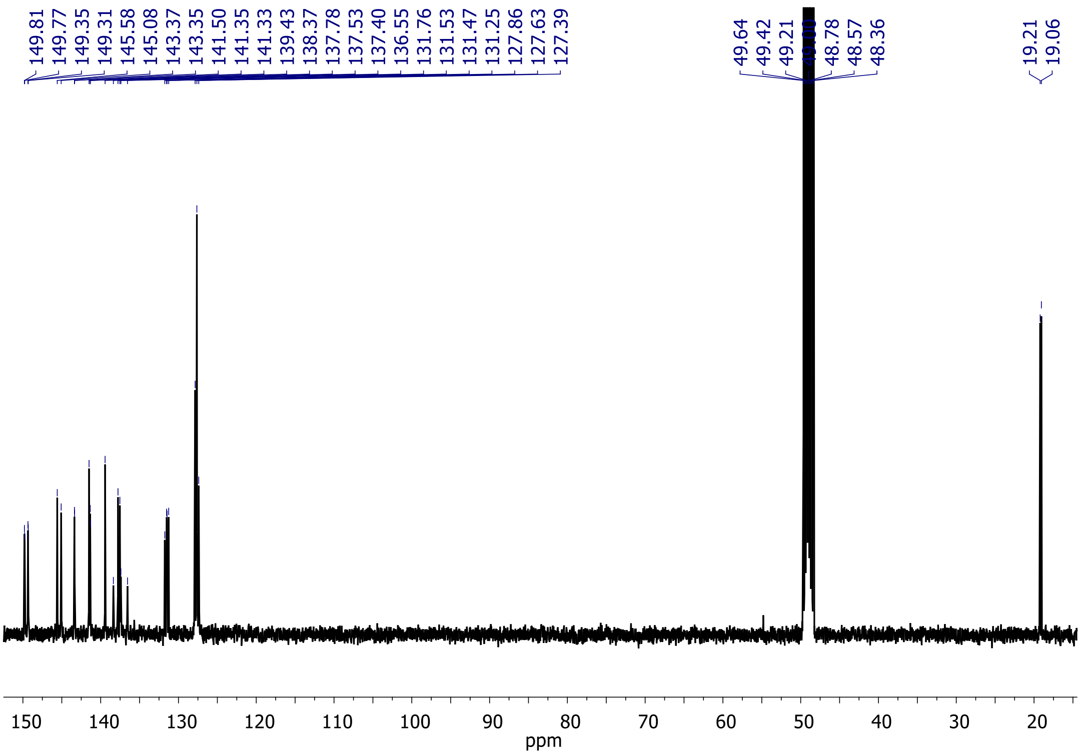

Figure S2. ^{13}C NMR spectrum of **2** in MeOH-d$_4$.

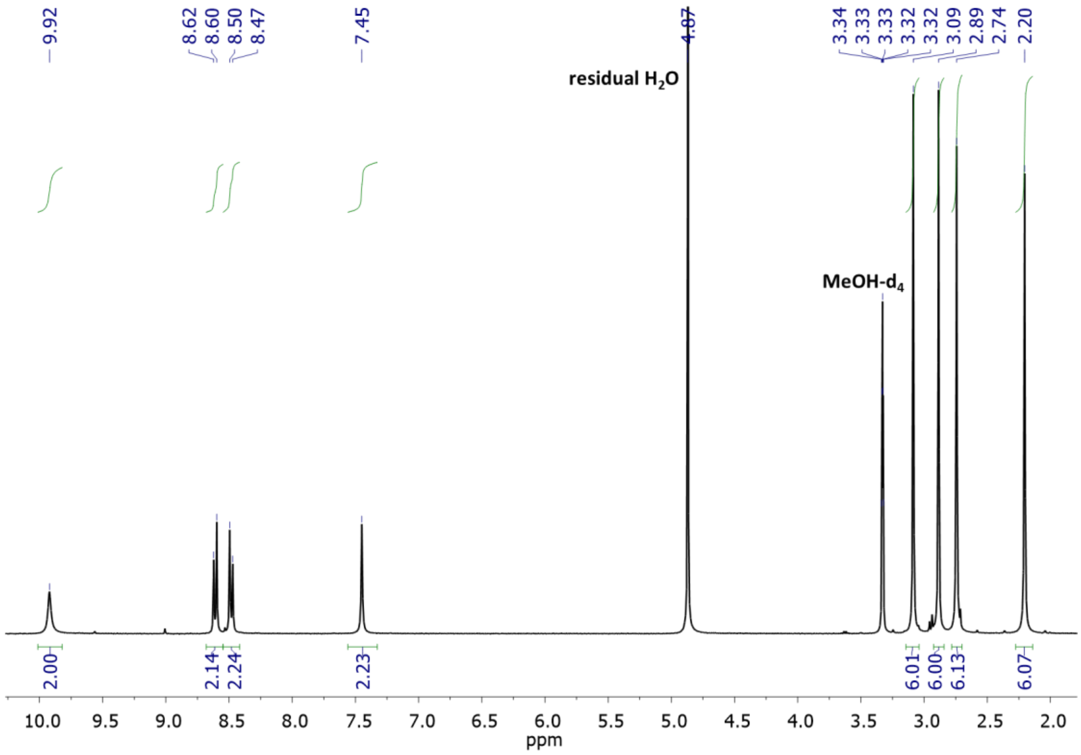

Figure S3. ^{1}H NMR spectrum of **3** in MeOH-d4.

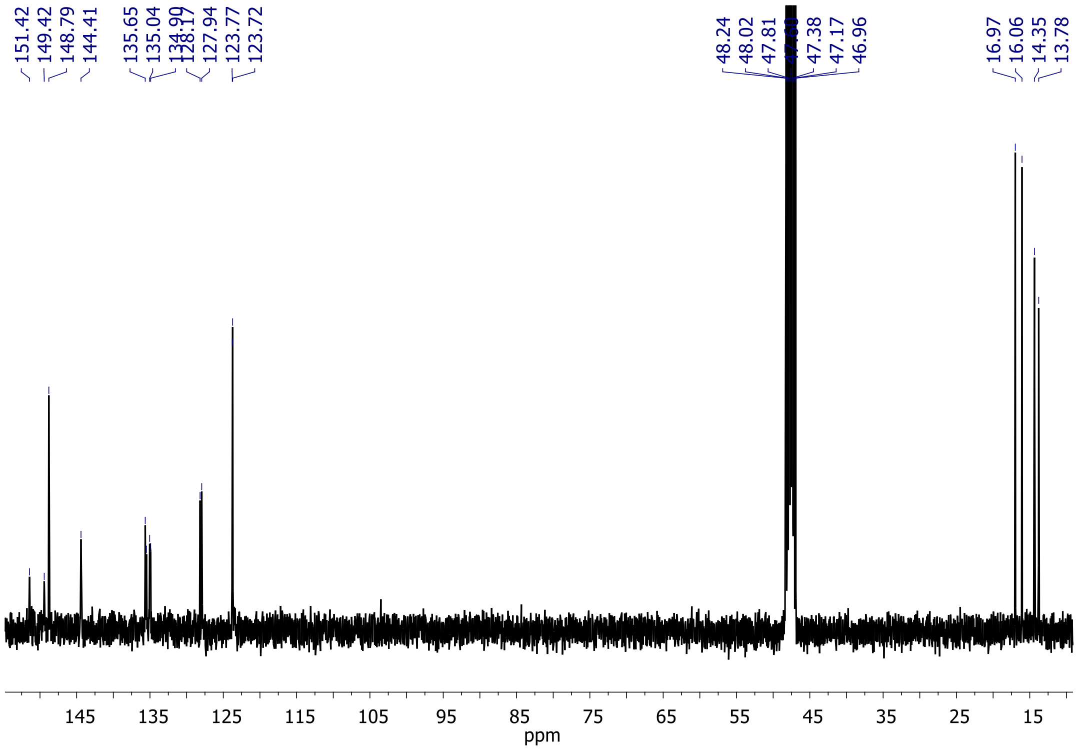

Figure S4. ^{13}C NMR spectrum of **3** in MeOH-d4.

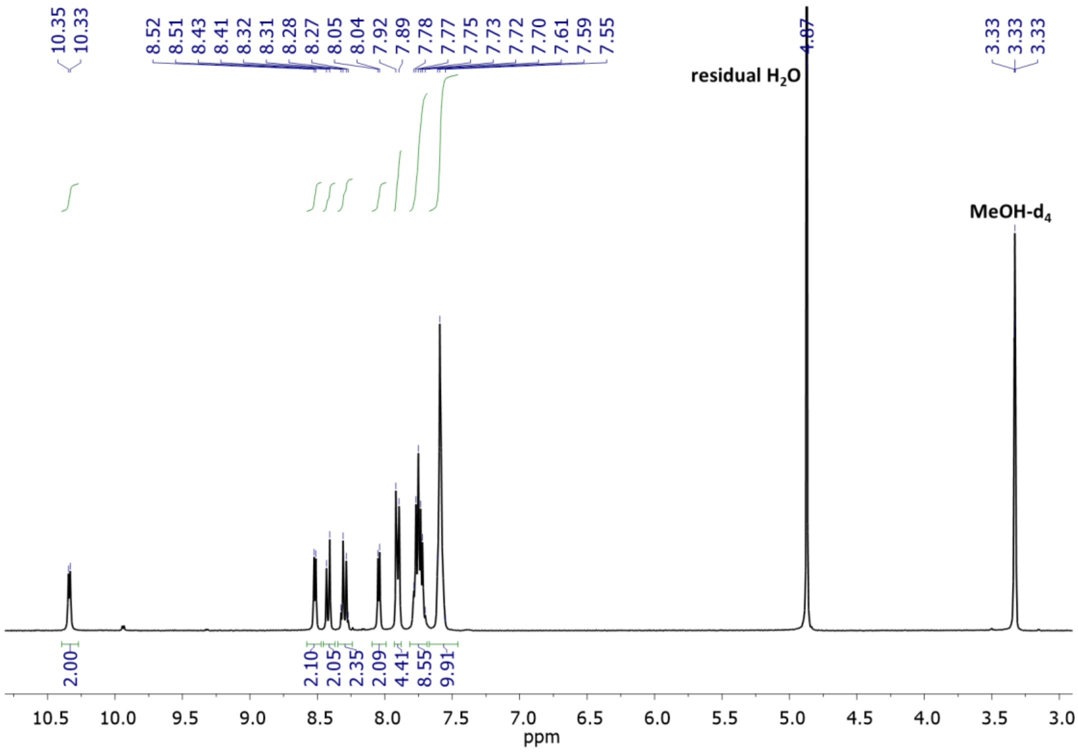

Figure S5. ^{1}H NMR spectrum of **4** in MeOH-d4.

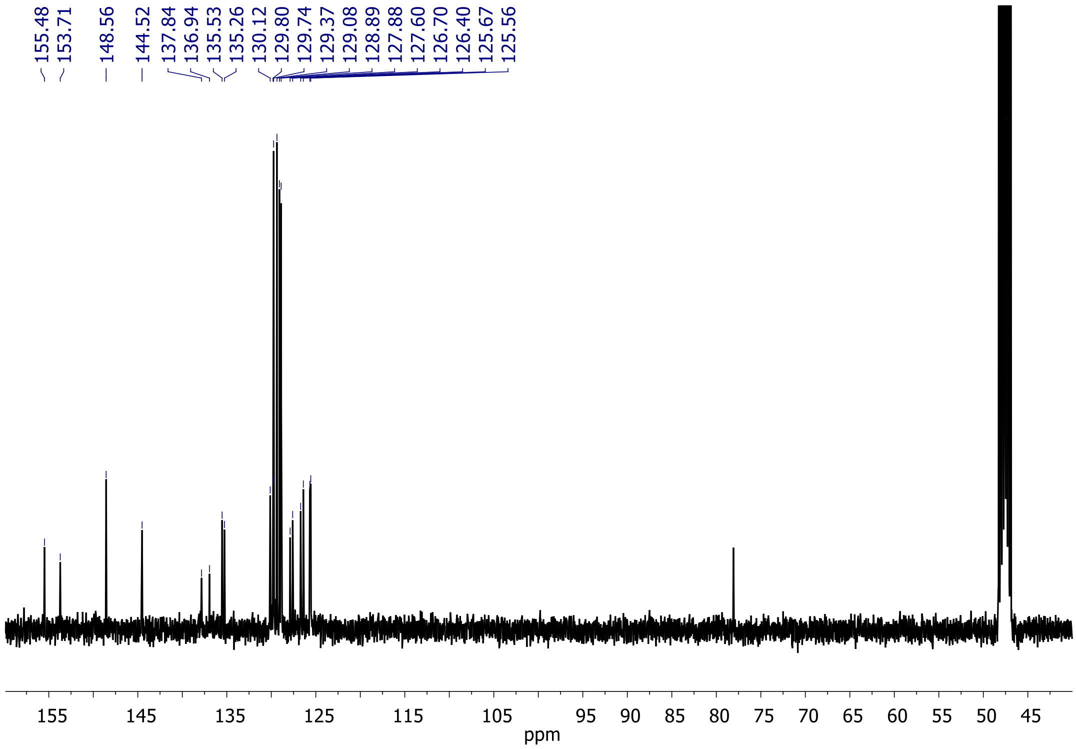

Figure S6. ^{13}C NMR spectrum of **4** in MeOH-d4.

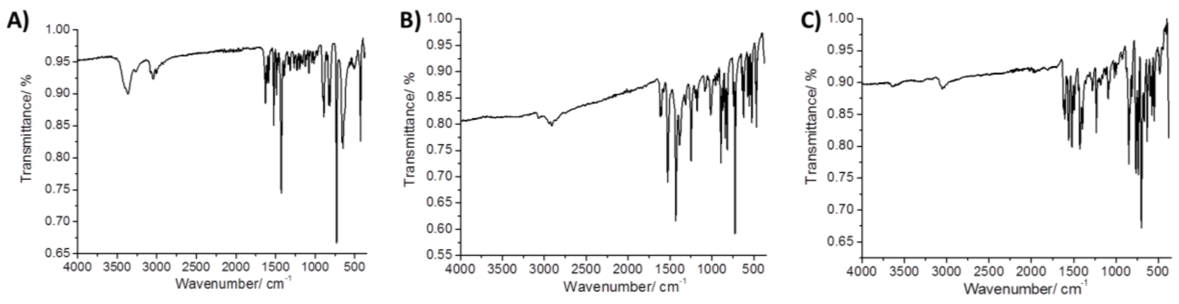

Figure S7. IR spectrum of (A) **2**, (B) **3**, and (C) **4** in the solid form.

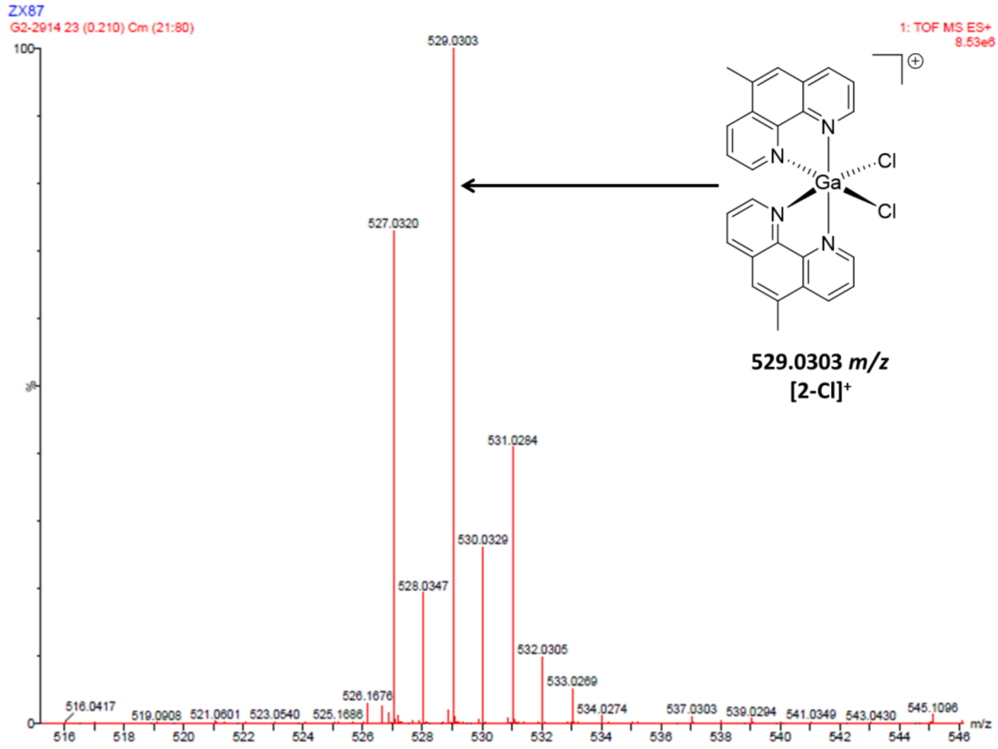

Figure S8. ESI mass spectrum (positive mode) of **2**.

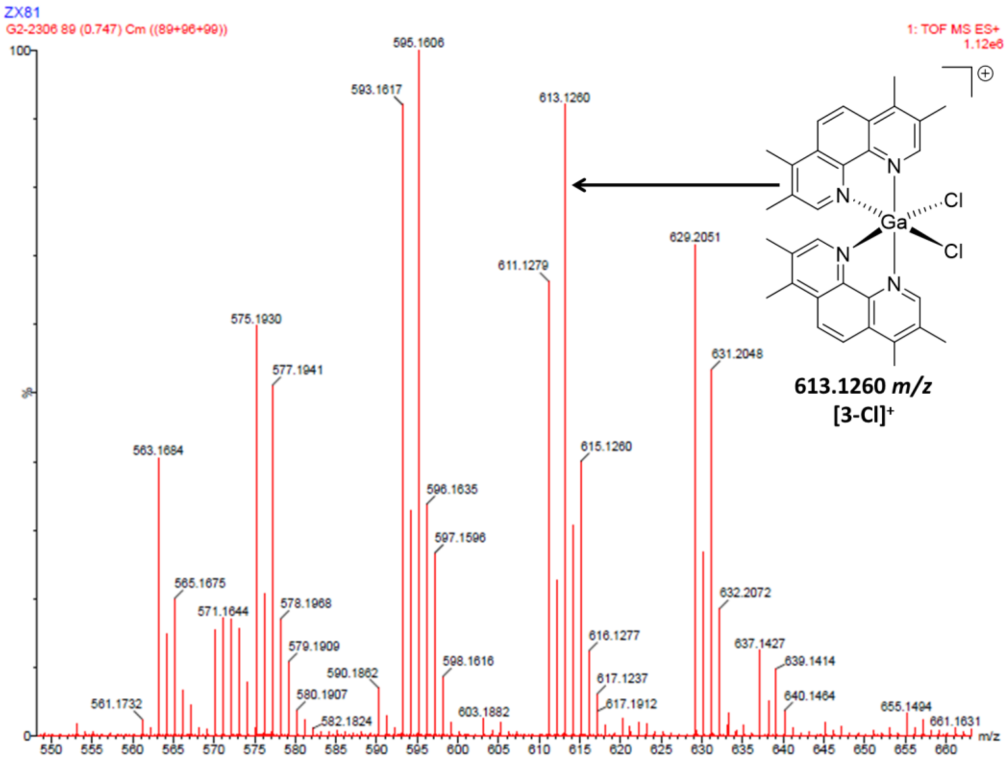

Figure S9. ESI mass spectrum (positive mode) of **3**.

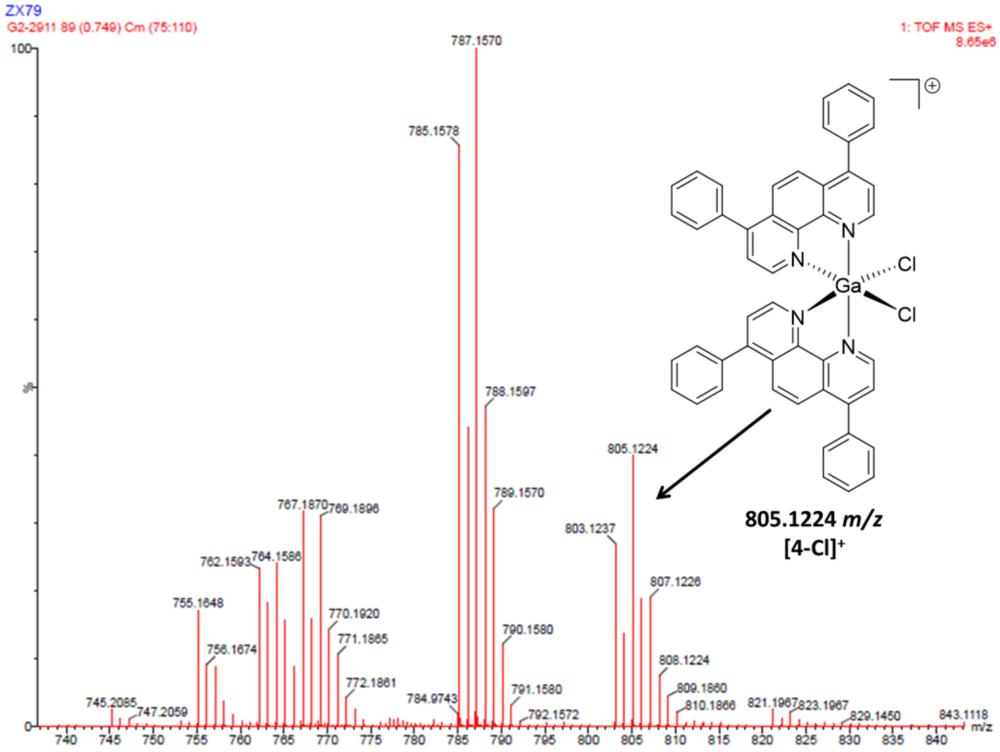

Figure S10. ESI mass spectrum (positive mode) of **4**.

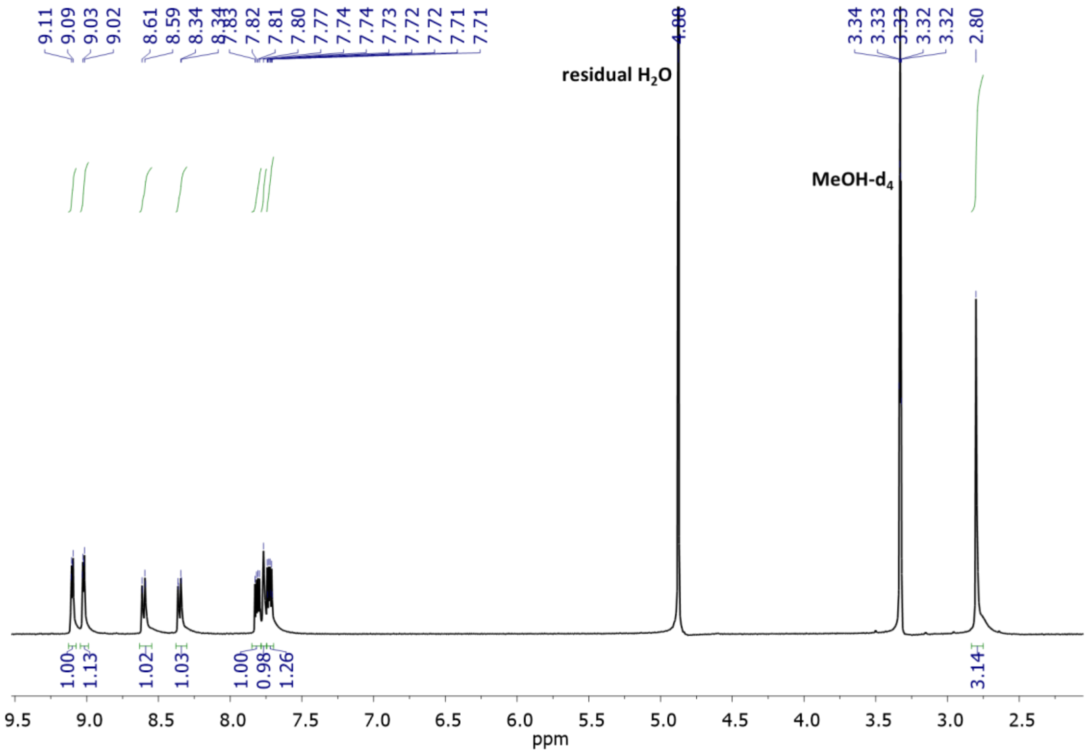

Figure S11. ^{1}H NMR spectrum of 5-methyl-1,10-phenanthroline in MeOH-d_4.

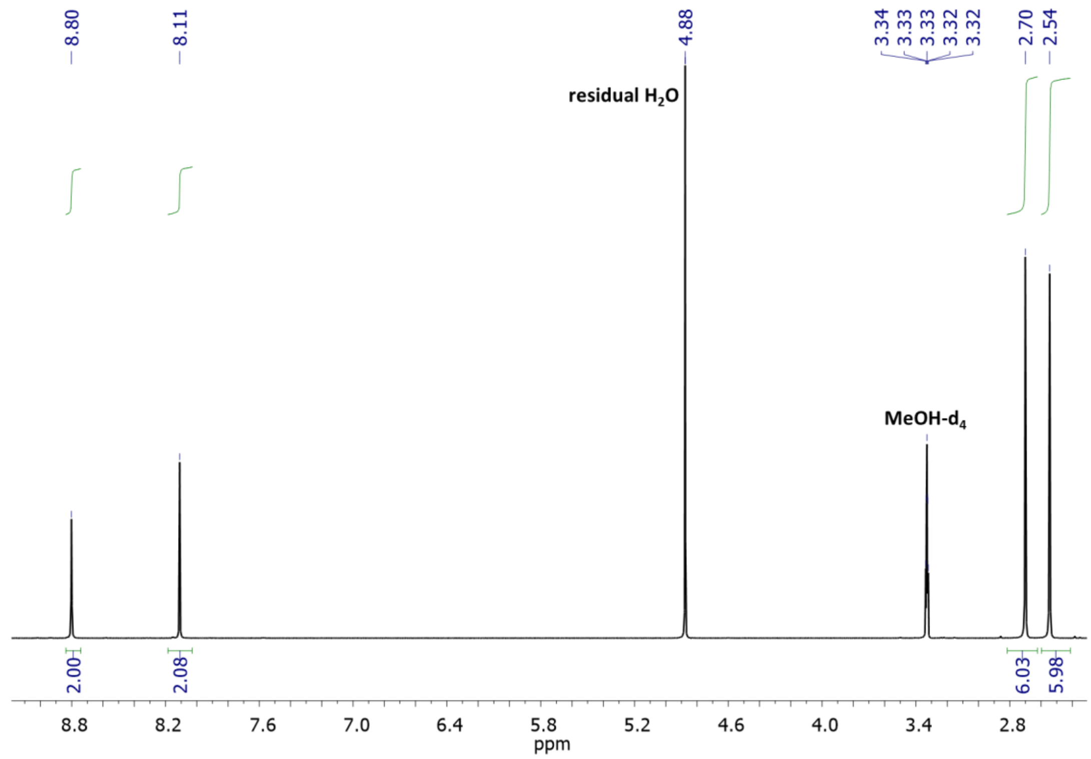

Figure S12. ^{1}H NMR spectrum of 3,4,7,8-tetramethyl-1,10-phenanthroline in MeOH-d_4.

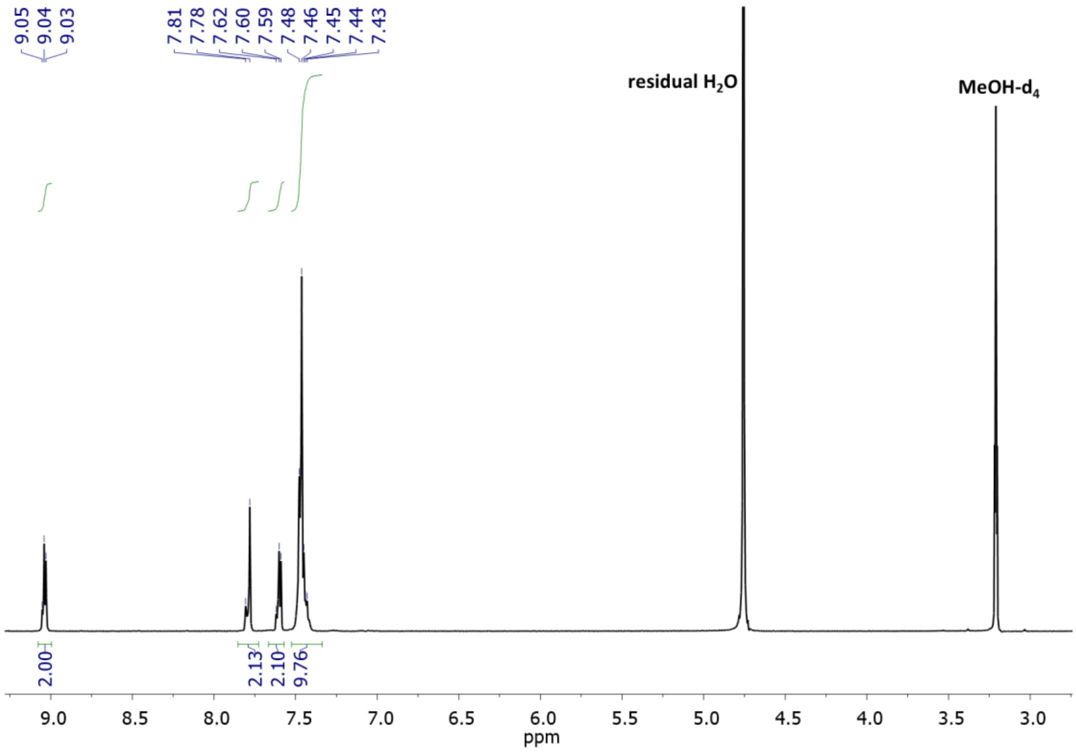

Figure S13. ^{1}H NMR spectrum of 4,7-diphenyl-1,10-phenanthroline in MeOH-d_4.

Table S1. Crystallographic data for complex **4**.

**Metal complex**	**4**
CCDC No.	2086778
formula	$4C_{48}H_{32}N_4Cl_3Ga•2CH_2Cl_2•5CH_3OH$
Fw	3693.44
Crystal system	monoclinic
Space group	P21/c
a, Å	13.478(6)
b, Å	14.092(6)
c, Å	25.222(11)
α, deg.	90
β, deg.	104.744(10)
γ, deg.	90
V, Å^3	4633(4)
Z	1
D_{calcd}, Mg/m^3	1.324
2θ / deg.	3.34 to 51.996
Reflections collected	35881
Independent reflections	9096
Goodness-of-fit on F^2	0.845
R_1, wR_2 [$I \geq 2\sigma(I)$]	0.0660, 0.1515
R_1, wR_2 [all data]	0.1479, 0.1780

Table S2. Selected bond lengths (Å) and angles (°) for complex **4**.

Ga(1)-N(3)	2.073(4)	Ga(1)-N(2)	2.142(4)
Ga(1)-N(1)	2.076(4)	Ga(1)-Cl(2)	2.2736(17)
Ga(1)-N(4)	2.119(4)	Ga(1)-Cl(1)	2.2821(17)
N(3)-Ga(1)-N(1)	166.44(17)	N(4)-Ga(1)-Cl(2)	89.66(13)
N(3)-Ga(1)-N(4)	78.37(17)	N(2)-Ga(1)-Cl(2)	170.71(12)
N(1)-Ga(1)-N(4)	94.58(16)	N(3)-Ga(1)-Cl(1)	94.48(13)
N(3)-Ga(1)-N(2)	89.71(16)	N(1)-Ga(1)-Cl(1)	91.04(12)
N(1)-Ga(1)-N(2)	78.00(17)	N(4)-Ga(1)-Cl(1)	170.30(13)
N(4)-Ga(1)-N(2)	84.48(16)	N(2)-Ga(1)-Cl(1)	88.98(13)
N(3)-Ga(1)-Cl(2)	96.18(12)	Cl(2)-Ga(1)-Cl(1)	97.68(7)
N(1)-Ga(1)-Cl(2)	95.34(13)		

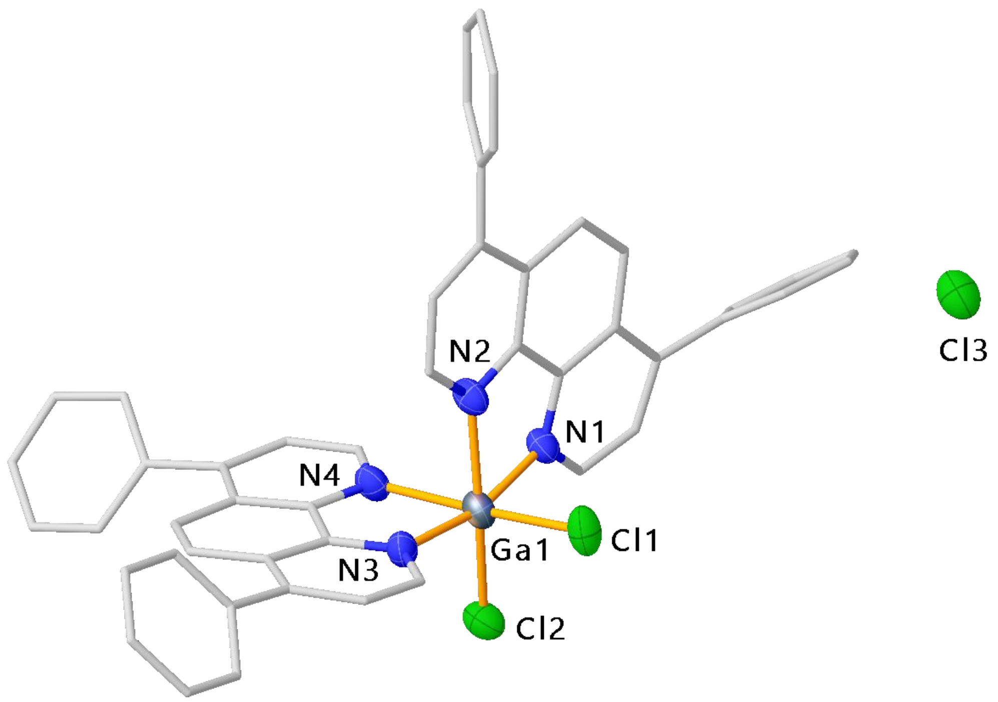

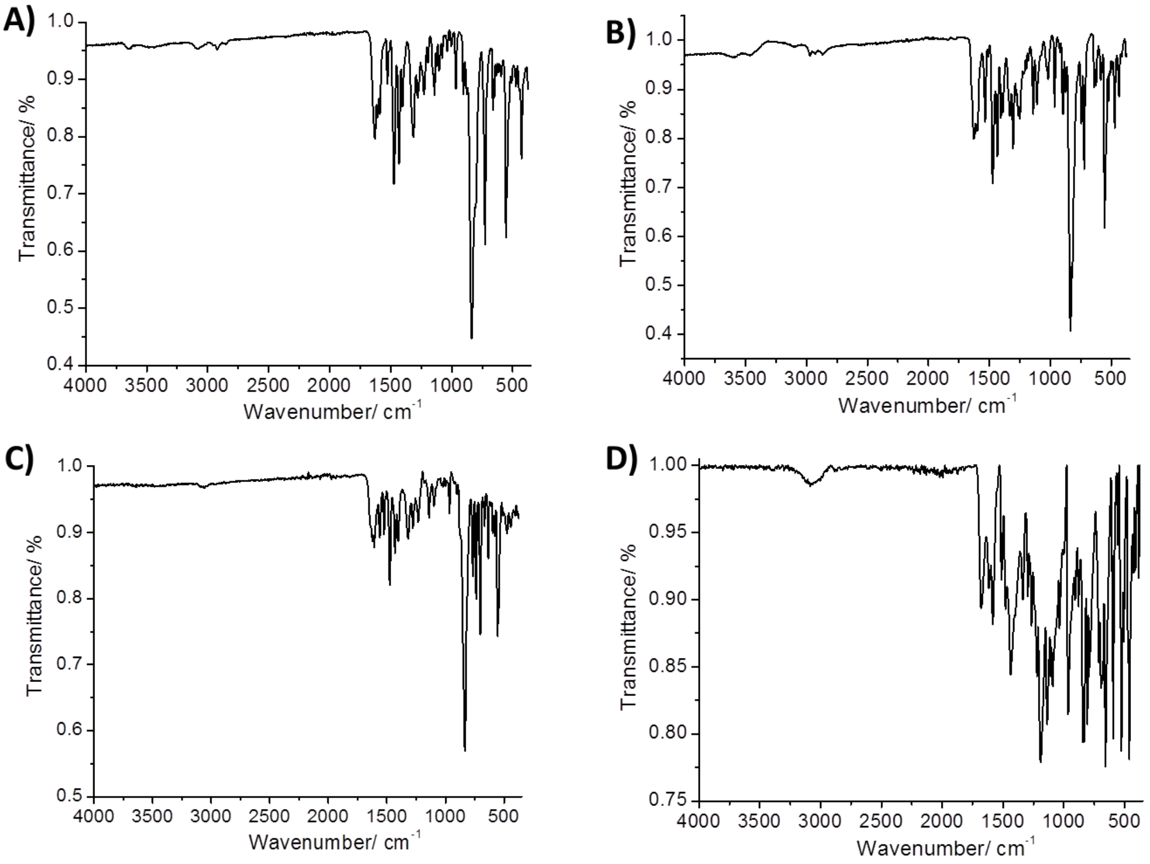

Figure S14. IR spectrum of (A) **5**, (B) **6**, (C) **7**, and (D) diflunisal in the solid form.

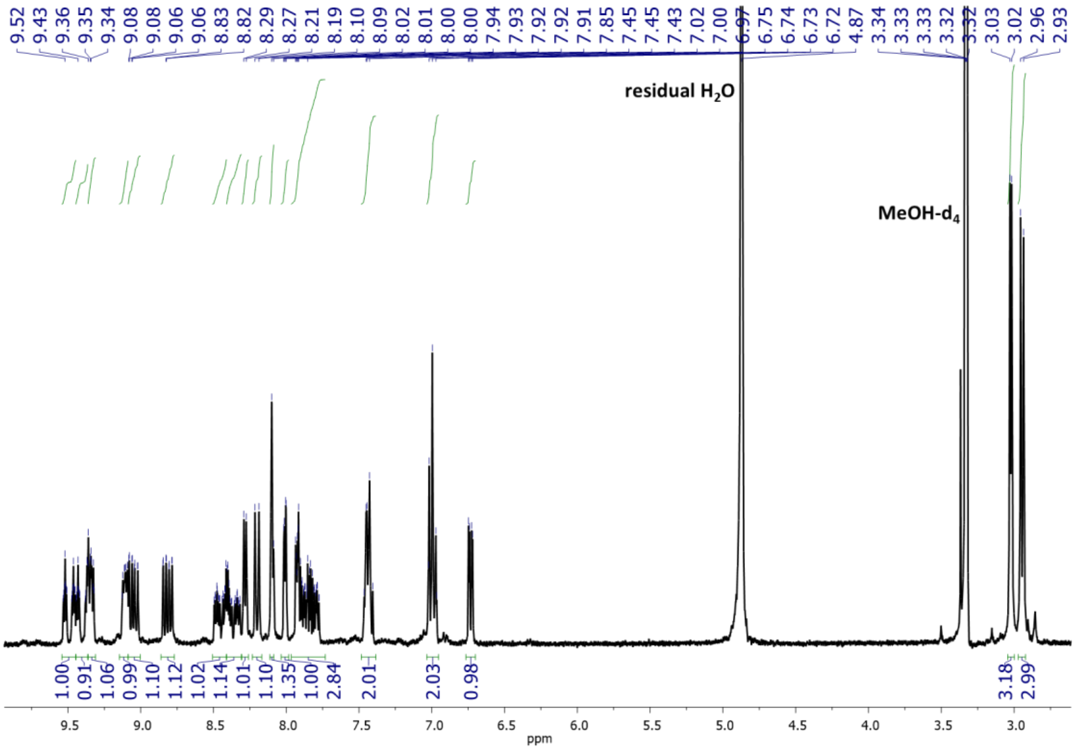

Figure S15. ^{1}H NMR spectrum of **5** in MeOH-d_4.

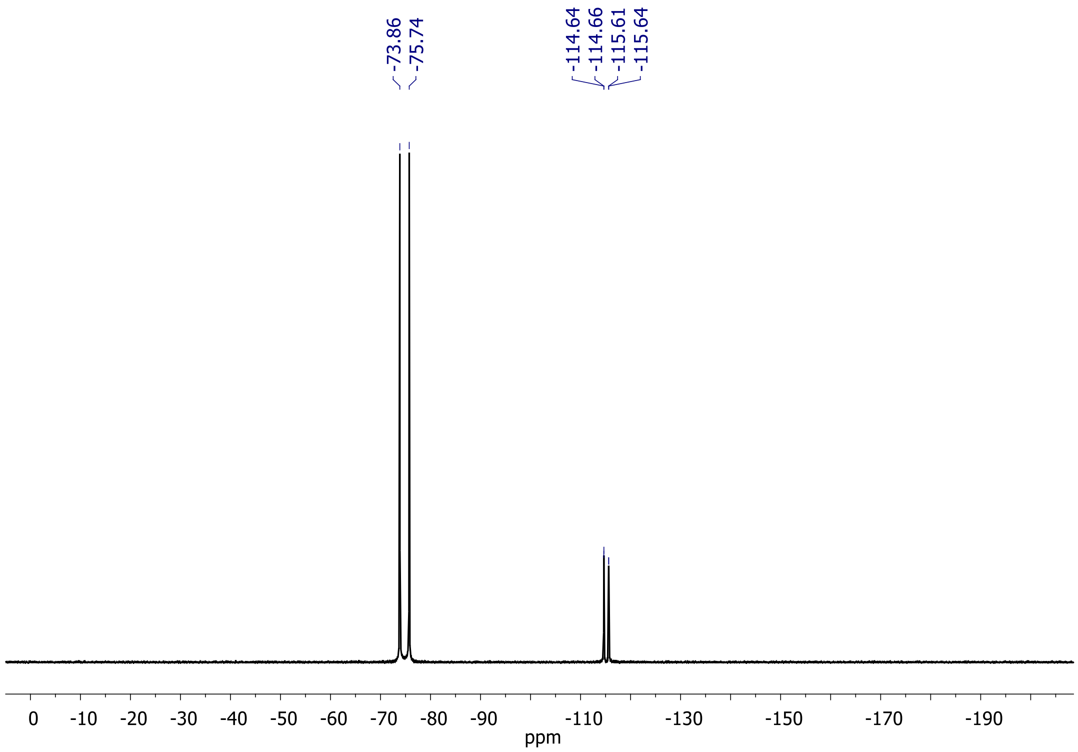

Figure S16. $^{19}F\{^{1}H\}$ NMR spectrum of **5** in MeOH-d4.

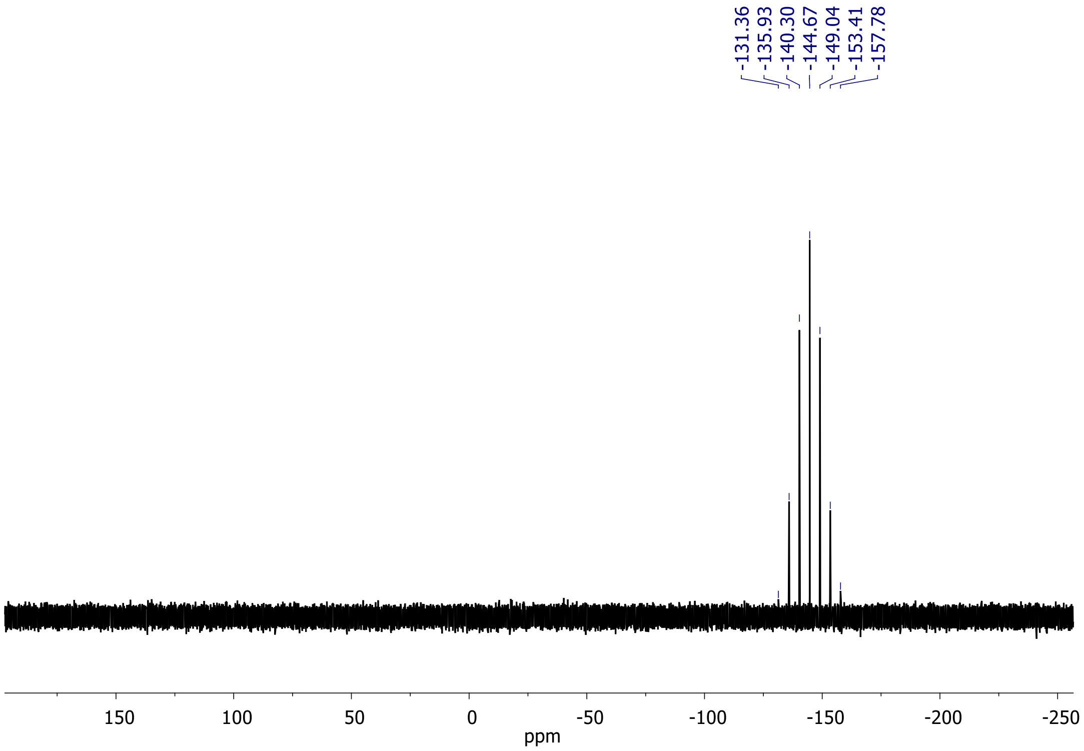

Figure S17. $^{31}P\{^{1}H\}$ NMR spectrum of **5** in MeOH-d4.

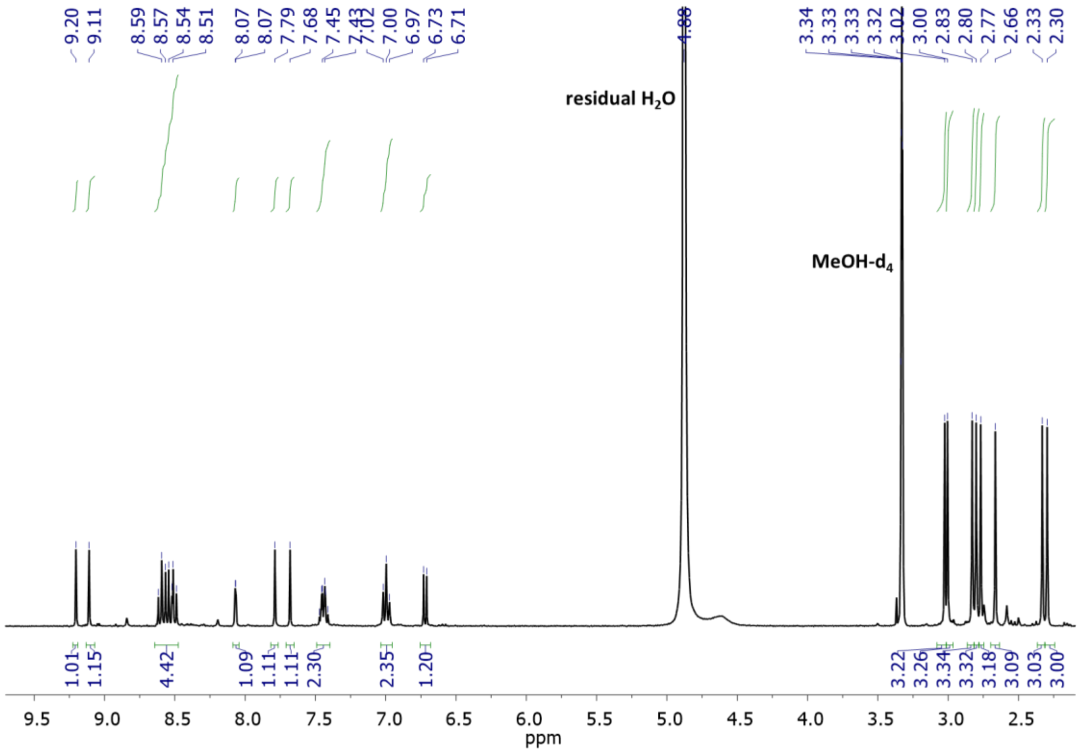

Figure S18. ^{1}H NMR spectrum of **6** in MeOH-d$_4$.

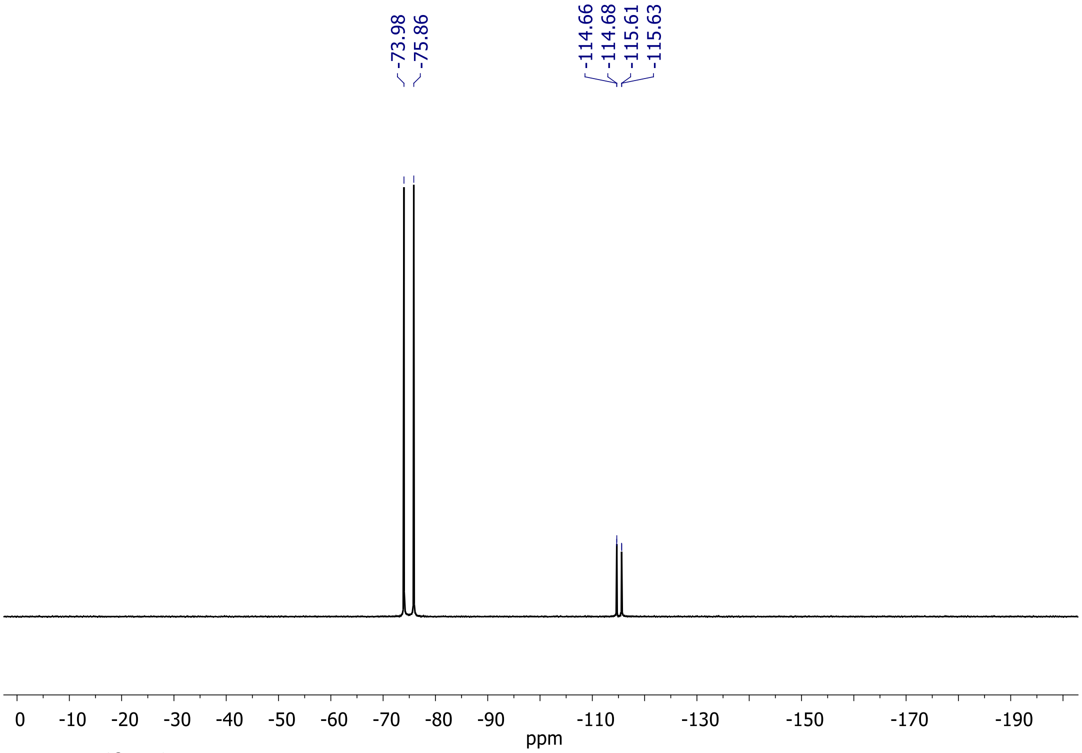

Figure S19. ^{19}F{^{1}H} NMR spectrum of **6** in MeOH-d$_4$.

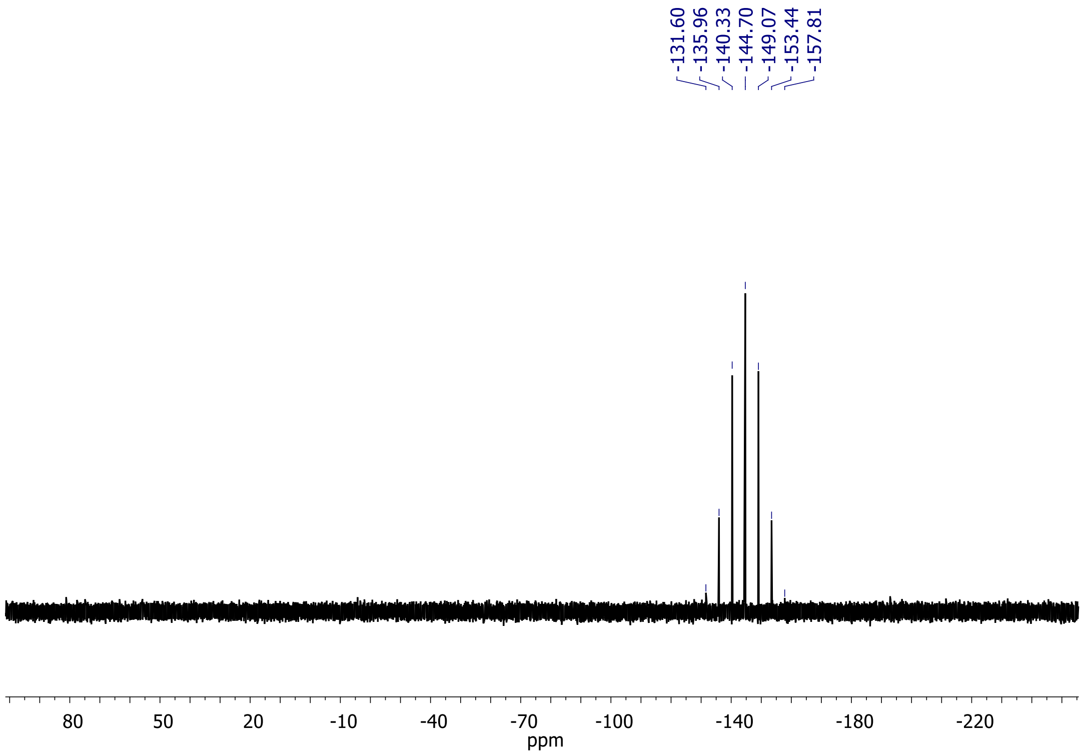

Figure S20. $^{31}P\{^{1}H\}$ NMR spectrum of **6** in MeOH-d4.

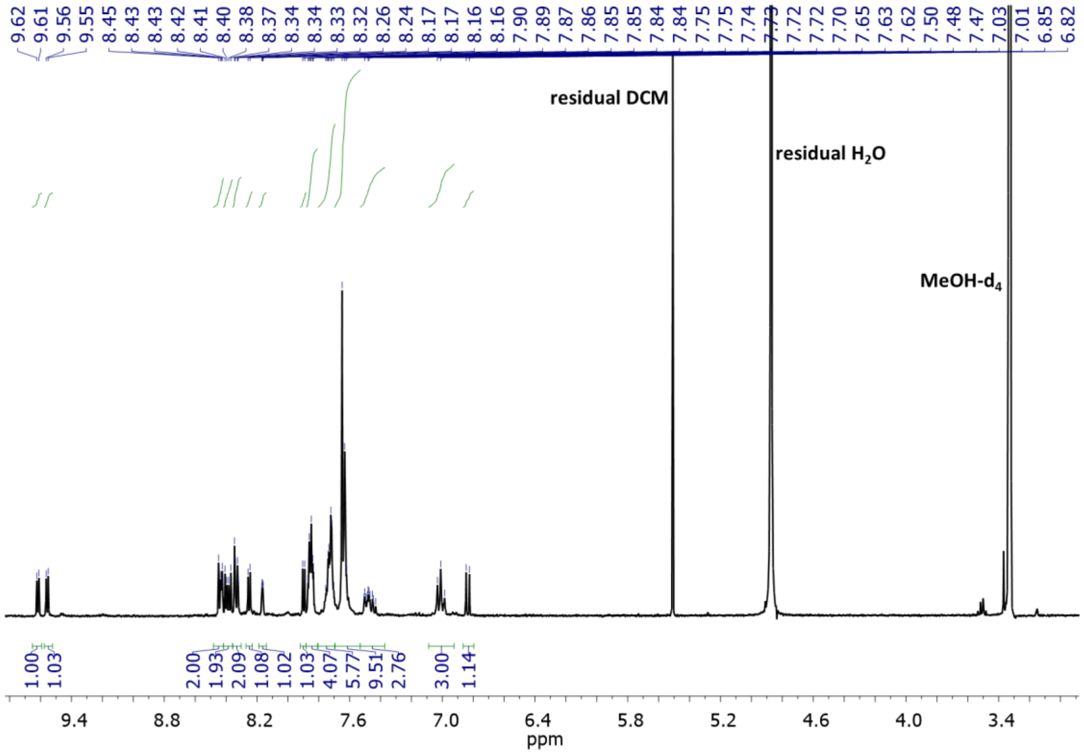

Figure S21. ^{1}H NMR spectrum of **7** in MeOH-d4.

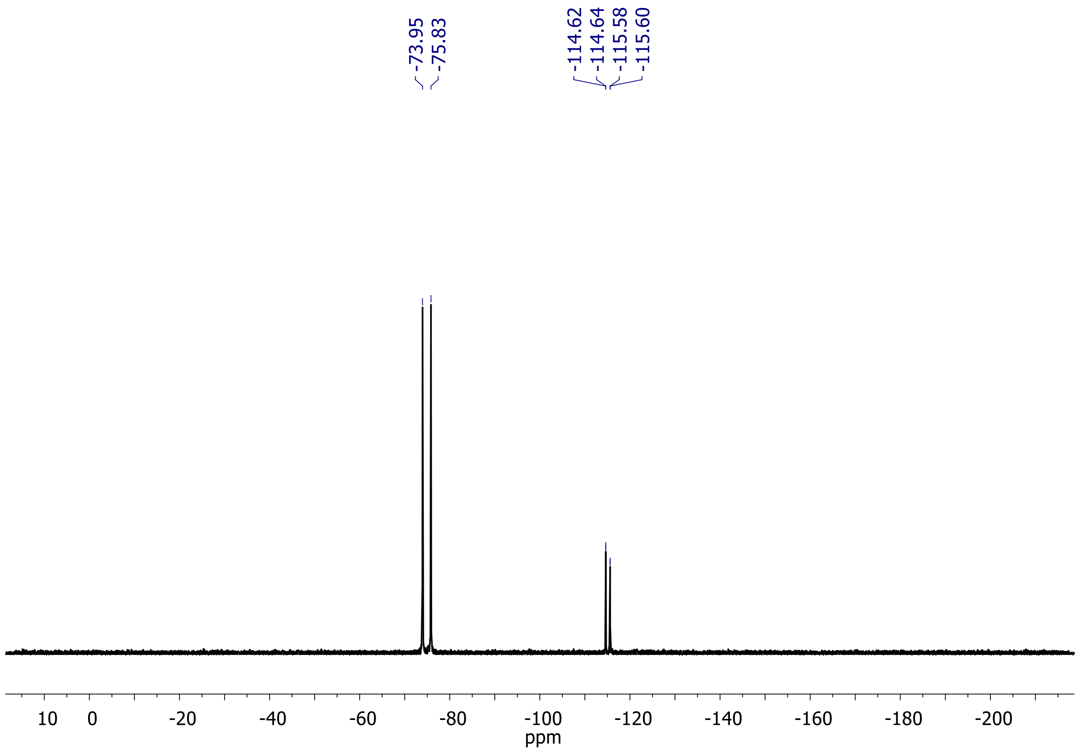

Figure S22. $^{19}F\{^1H\}$ NMR spectrum of **7** in MeOH-d_4.

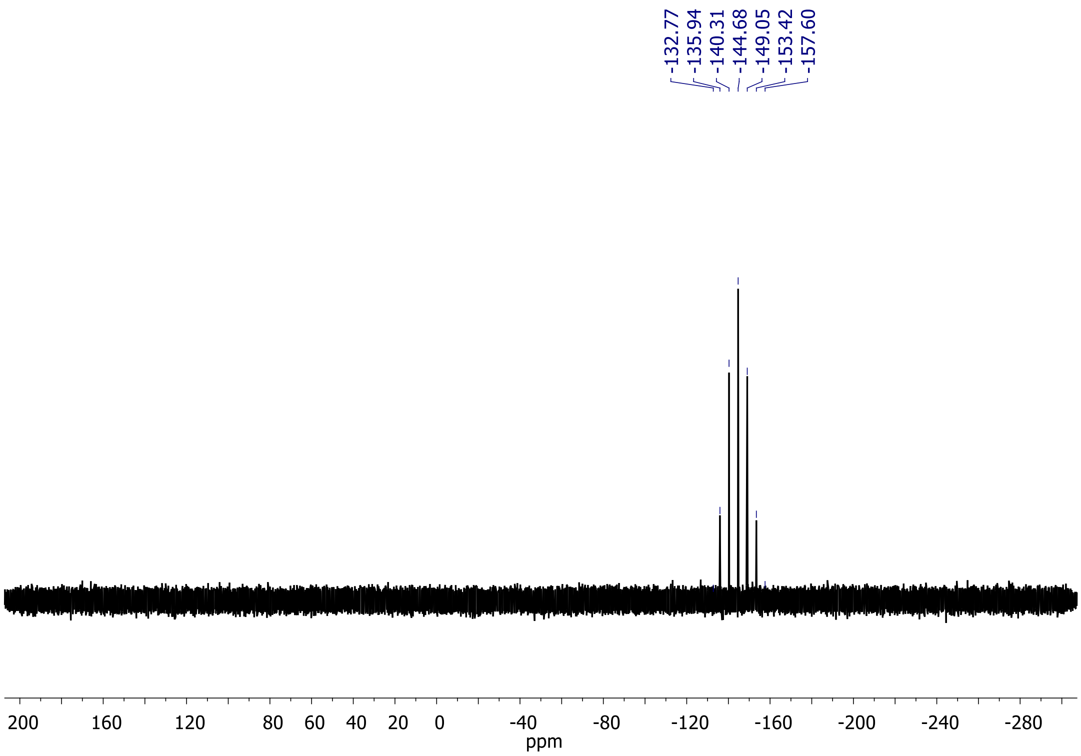

Figure S23. $^{31}P\{^1H\}$ NMR spectrum of **7** in MeOH-d_4.

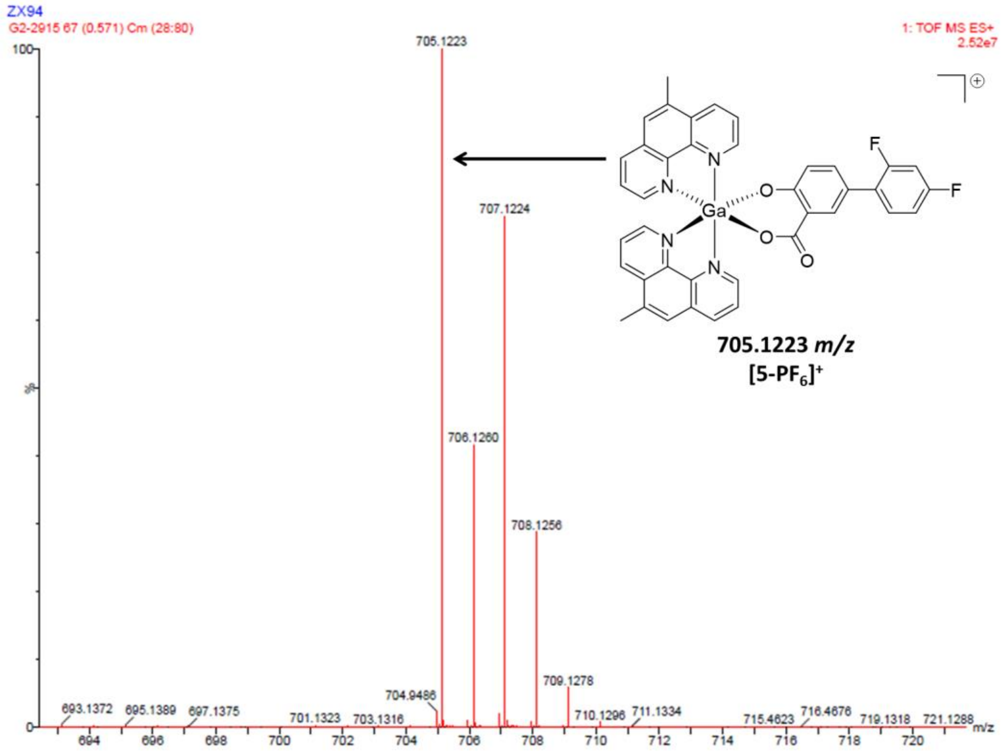

Figure S24. ESI mass spectrum (positive mode) of **5**.

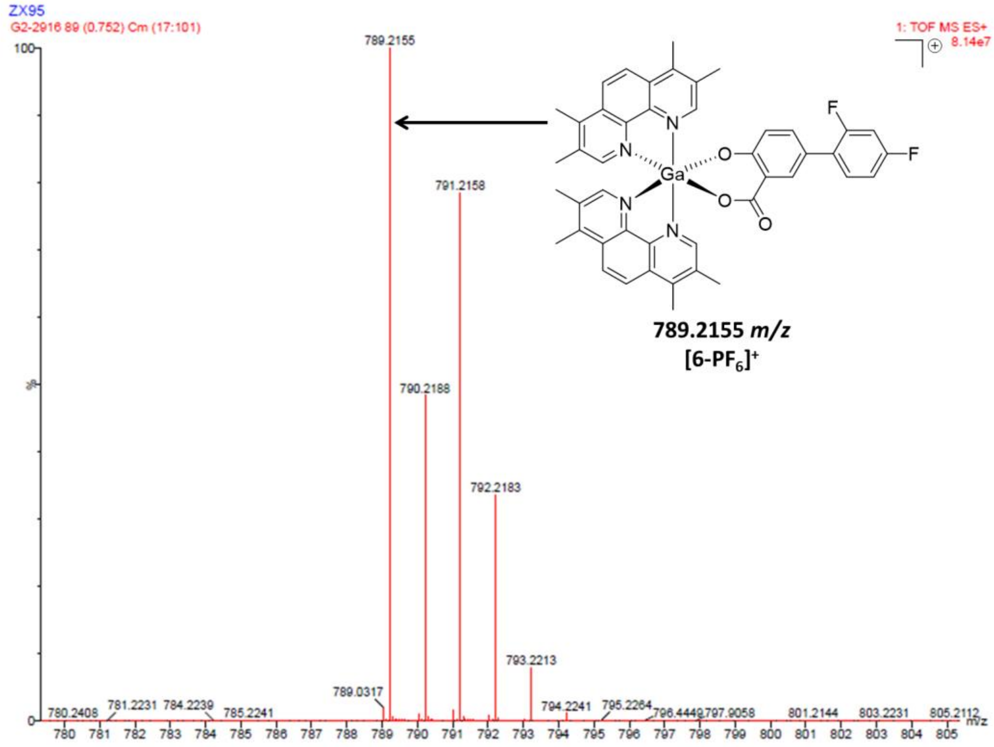

Figure S25. ESI mass spectrum (positive mode) of **6**.

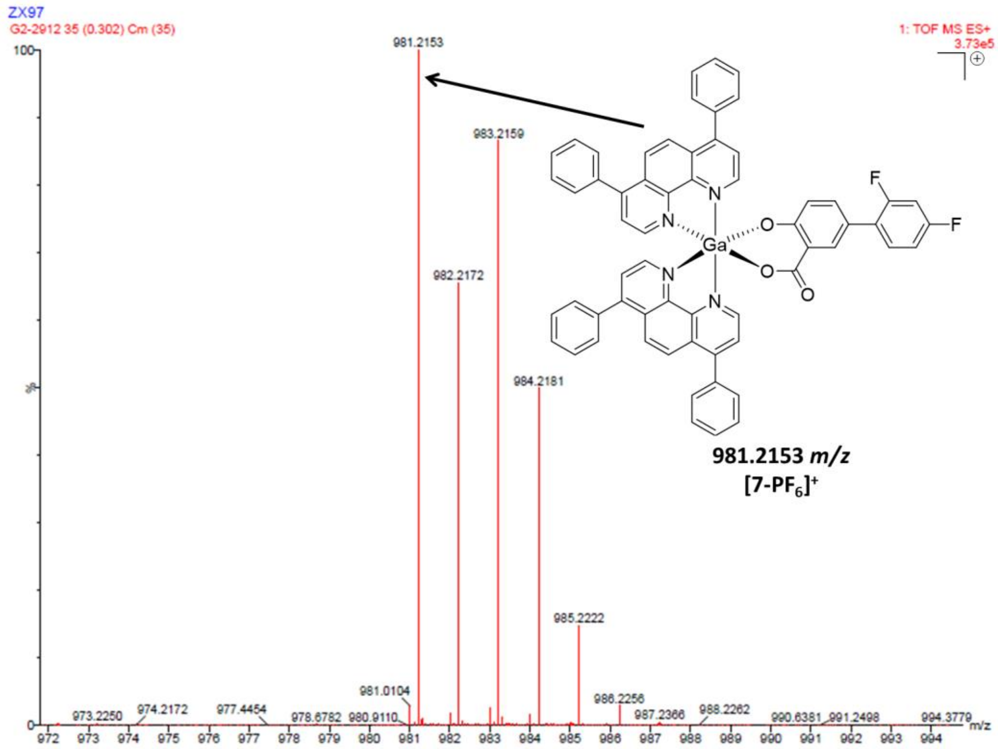

Figure S26. ESI mass spectrum (positive mode) of **7**.

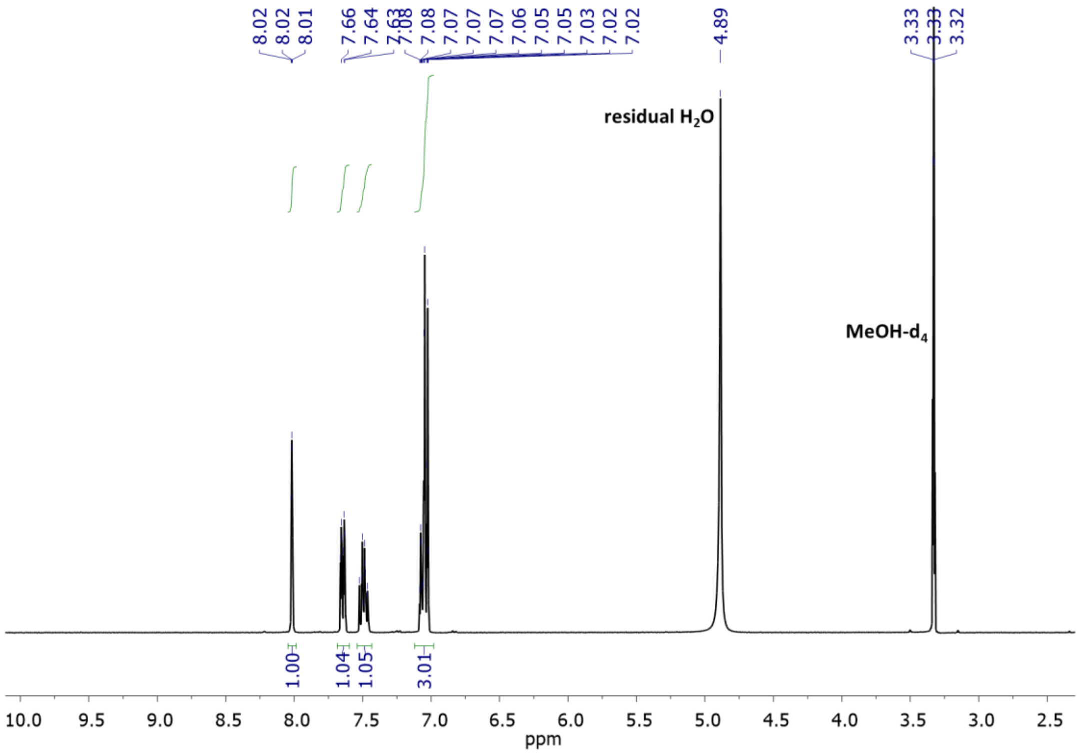

Figure S27. ^{1}H NMR spectrum of diflunisal in MeOH-d$_4$.

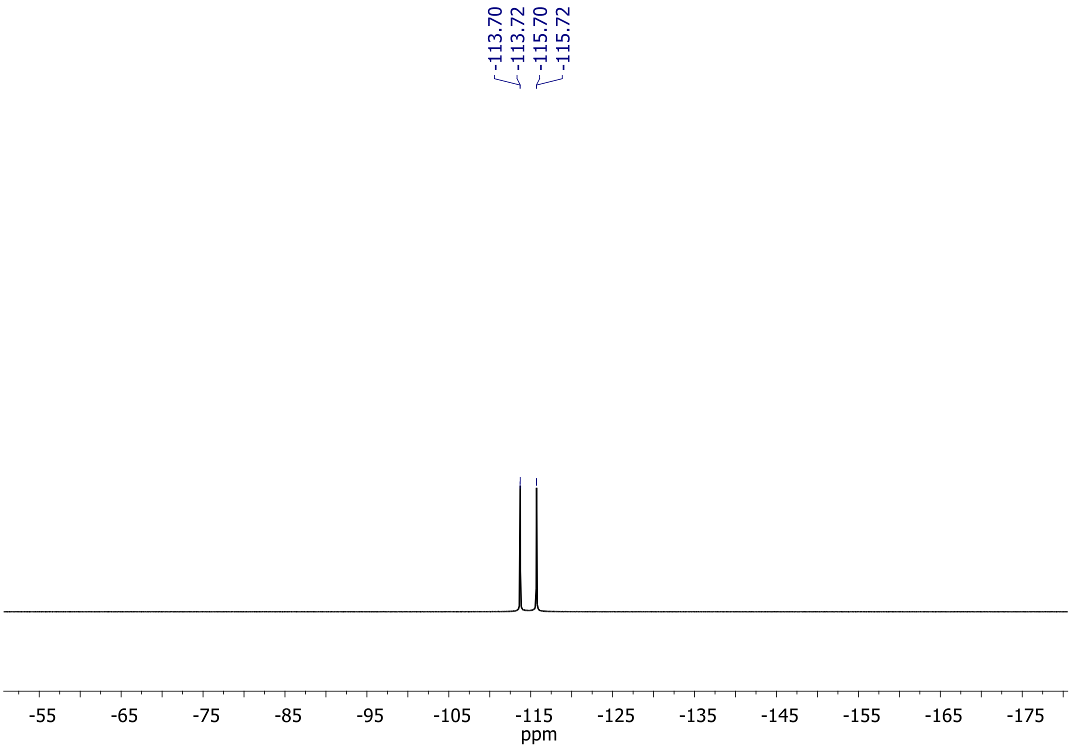

Figure S28. $^{19}F\{^1H\}$ NMR spectrum of diflunisal in MeOH-d_4.

Table S3. Crystallographic data for complex **6**.

**Metal complex**	**6**
CCDC No.	2086777
formula	$C_{45}H_{38}F_8GaN_4O_3P$•$3CH_3CN$•H_2O
Fw	1076.66
Crystal system	triclinic
Space group	*P-1*
a, Å	12.161(4)
b, Å	15.097(5)
c, Å	16.255(6)
α, deg.	100.880(8)
β, deg.	105.545(8)
γ, deg.	112.886(6)
V, $Å^3$	2500.4(15)
Z	2
D_{calcd}, Mg/m^3	1.430
2θ / deg.	2.754 to 52.0
Reflections collected	19663
Independent reflections	9690
Goodness-of-fit on F^2	0.922
R_1, wR_2 [$I \geq 2\sigma(I)$]	0.0868, 0.2264
R_1, wR_2 [all data]	0.1486, 0.2264

Table S4. Selected bond lengths (Å) and angles (°) for complex **6**.

Ga(1)-O(1)	1.904(5)	Ga(1)-N(2)	2.101(6)
Ga(1)-O(2)	1.901(5)	Ga(1)-N(3)	2.084(5)
Ga(1)-N(1)	2.081(5)	Ga(1)-N(4)	2.105(5)
O(1)-Ga(1)-O(2)	92.9(2)	O(2)-Ga(1)-N(4)	173.5(2)
O(1)-Ga(1)-N(1)	90.2(2)	N(1)-Ga(1)-N(2)	78.6(2)
O(1)-Ga(1)-N(2)	168.8(2)	N(1)-Ga(1)-N(3)	166.8(2)
O(1)-Ga(1)-N(3)	96.4(2)	N(1)-Ga(1)-N(4)	90.62(19)
O(1)-Ga(1)-N(4)	88.3(2)	N(2)-Ga(1)-N(3)	94.4(2)
O(2)-Ga(1)-N(1)	95.80(18)	N(2)-Ga(1)-N(4)	90.8(2)
O(2)-Ga(1)-N(2)	89.3(2)	N(3)-Ga(1)-N(4)	78.2(2)
O(2)-Ga(1)-N(3)	95.2(2)		

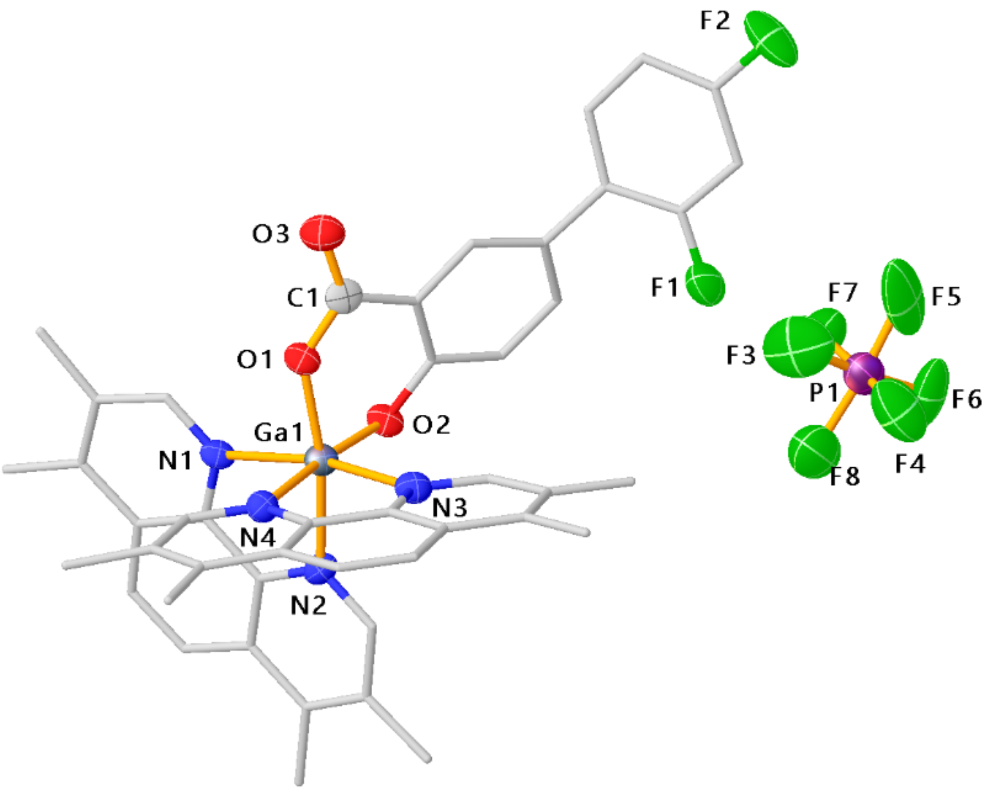

Table S5. Experimentally determined LogP values for **5**-**7**.

Metal complex	LogP
**5**	0.14 ± 0.03
**6**	0.04 ± 0.02
**7**	0.09 ±0.03

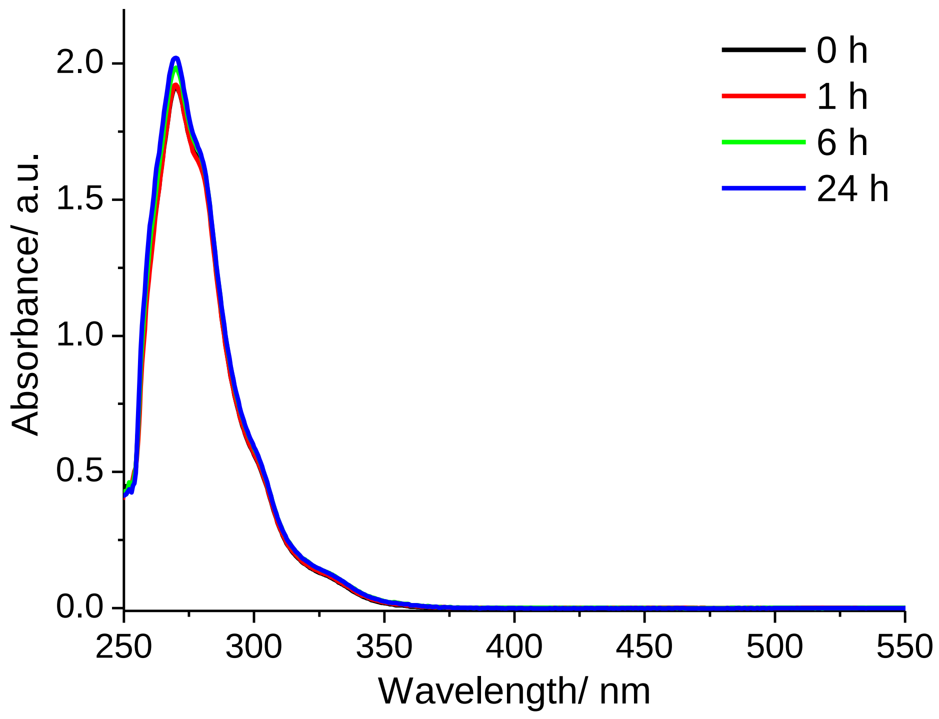

Figure S29. UV-Vis spectrum of **5** (25 μM) in DMSO over the course of 24 h at 37 °C.

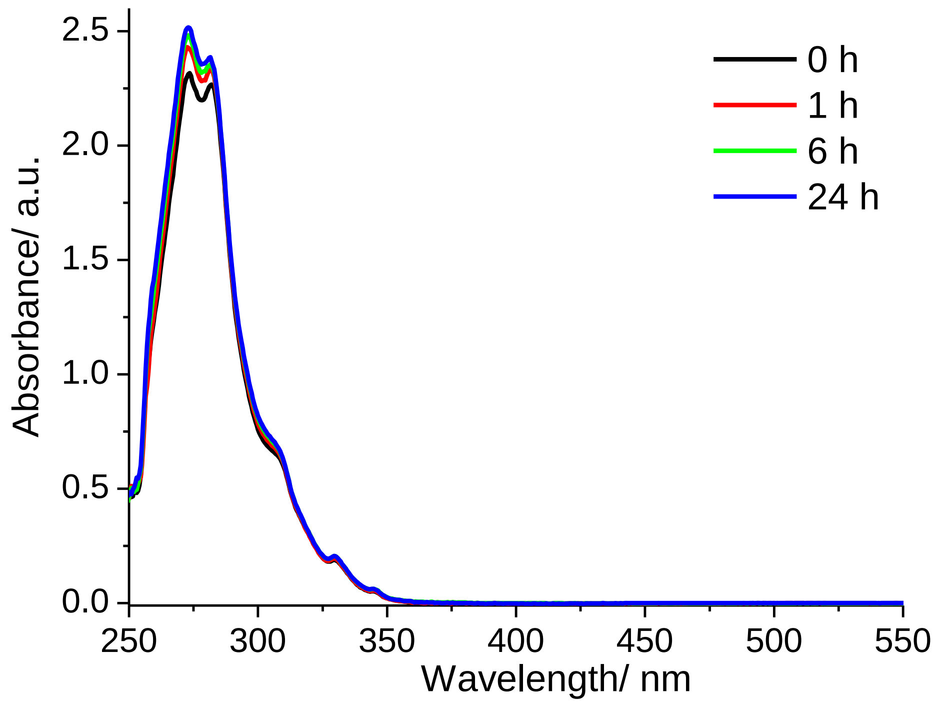

Figure S30. UV-Vis spectrum of **6** (25 μM) in DMSO over the course of 24 h at 37 °C.

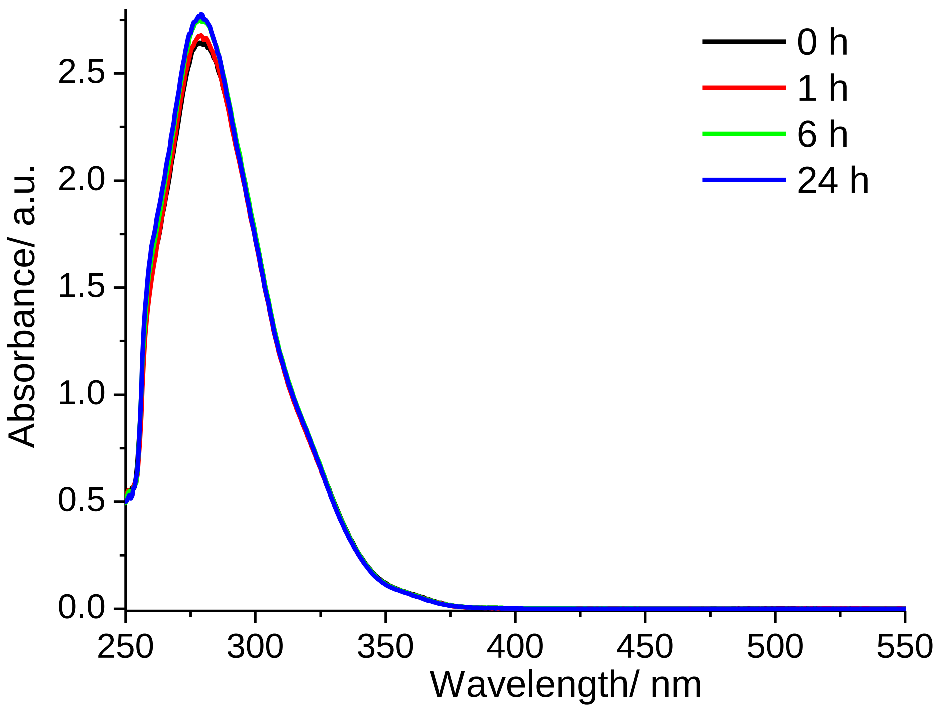

Figure S31. UV-Vis spectrum of **7** (25 μM) in DMSO over the course of 24 h at 37 °C.

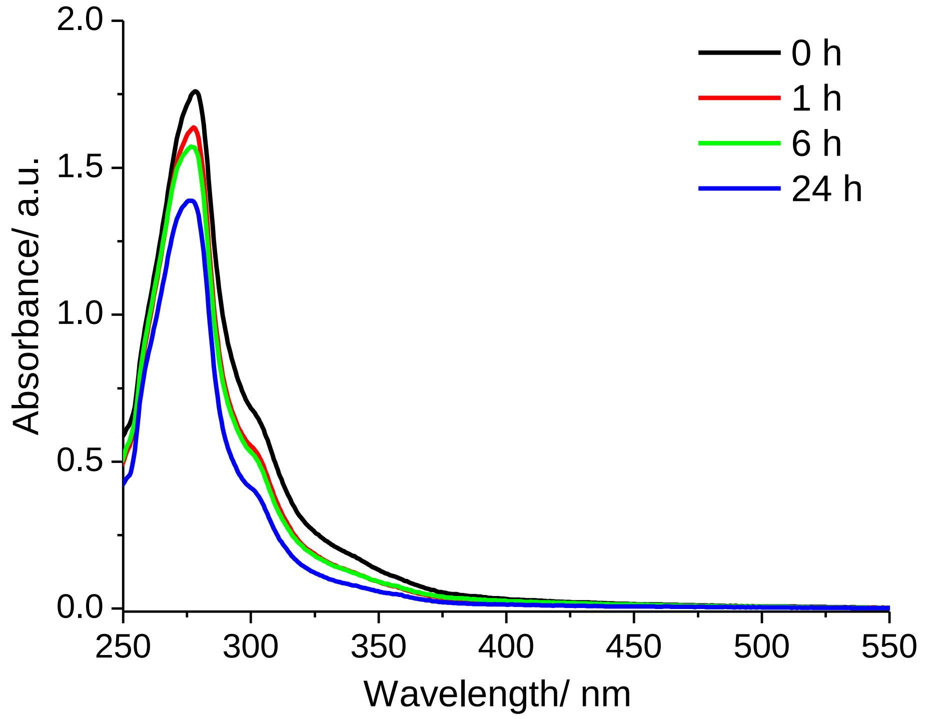

Figure S32. UV-Vis spectrum of **5** (25 μM) in H_2O:DMSO (200:1) over the course of 24 h at 37 ^oC.

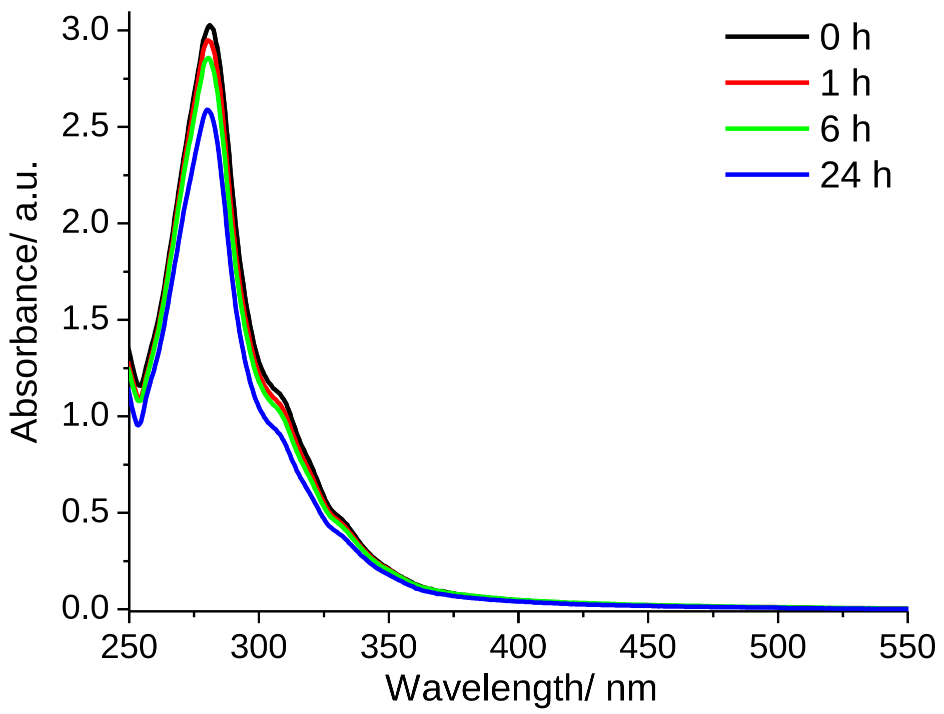

Figure S33. UV-Vis spectrum of **6** (25 μM) in H_2O:DMSO (200:1) over the course of 24 h at 37 ^oC.

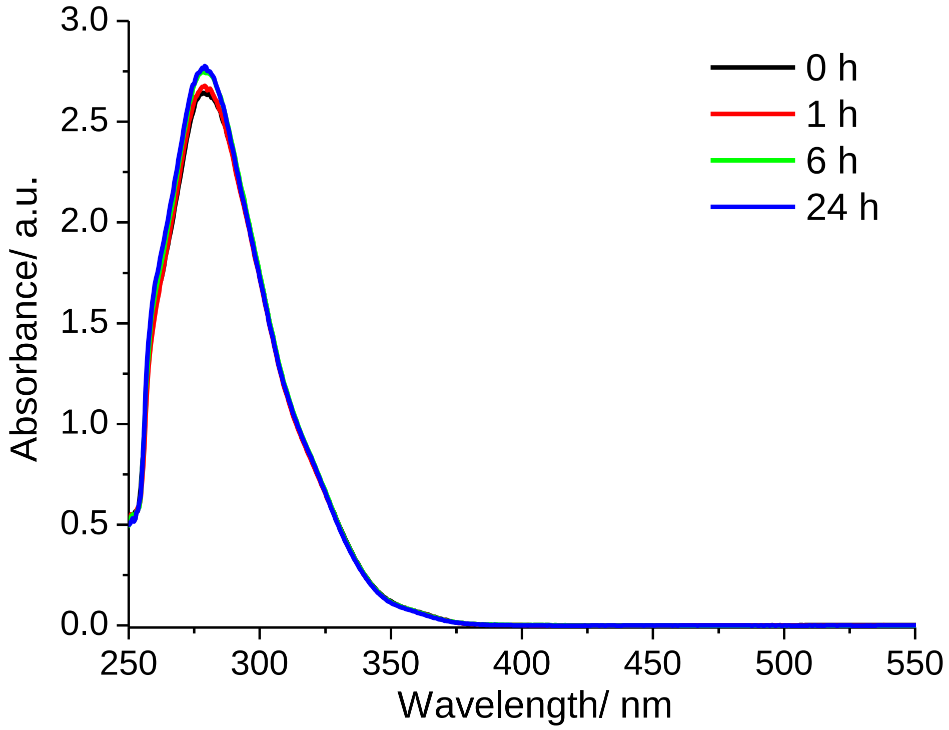

Figure S34. UV-Vis spectrum of **7** (25 μM) in H_2O:DMSO (200:1) over the course of 24 h at 37 ^oC.

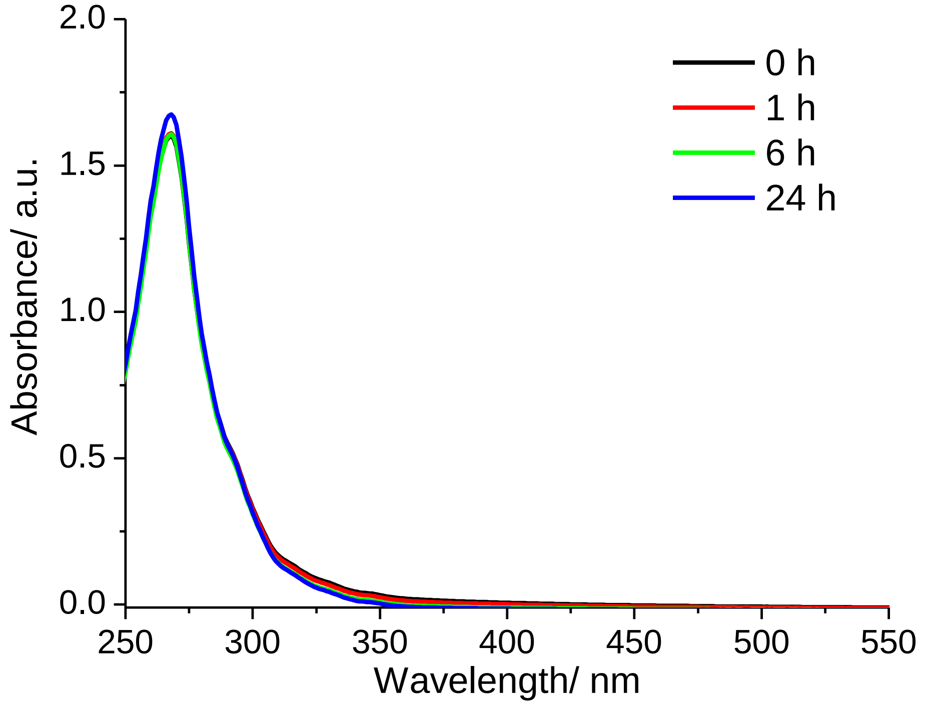

Figure S35. UV-Vis spectrum of **5** (25 μM) in PBS:DMSO (200:1) over the course of 24 h at 37 ^oC.

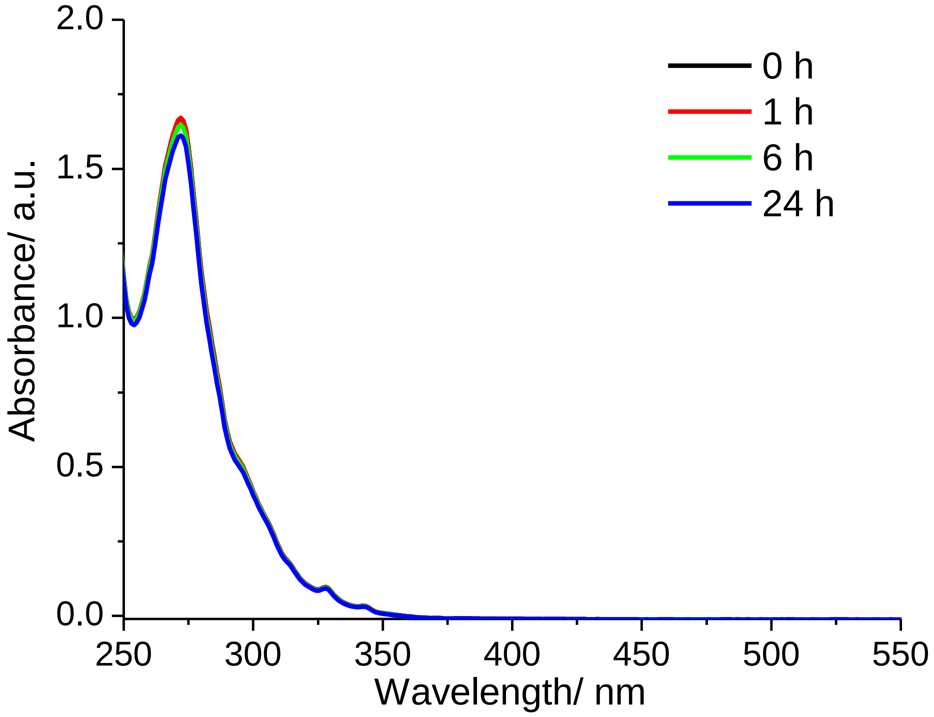

Figure S36. UV-Vis spectrum of **6** (25 μM) in PBS:DMSO (200:1) over the course of 24 h at 37 °C.

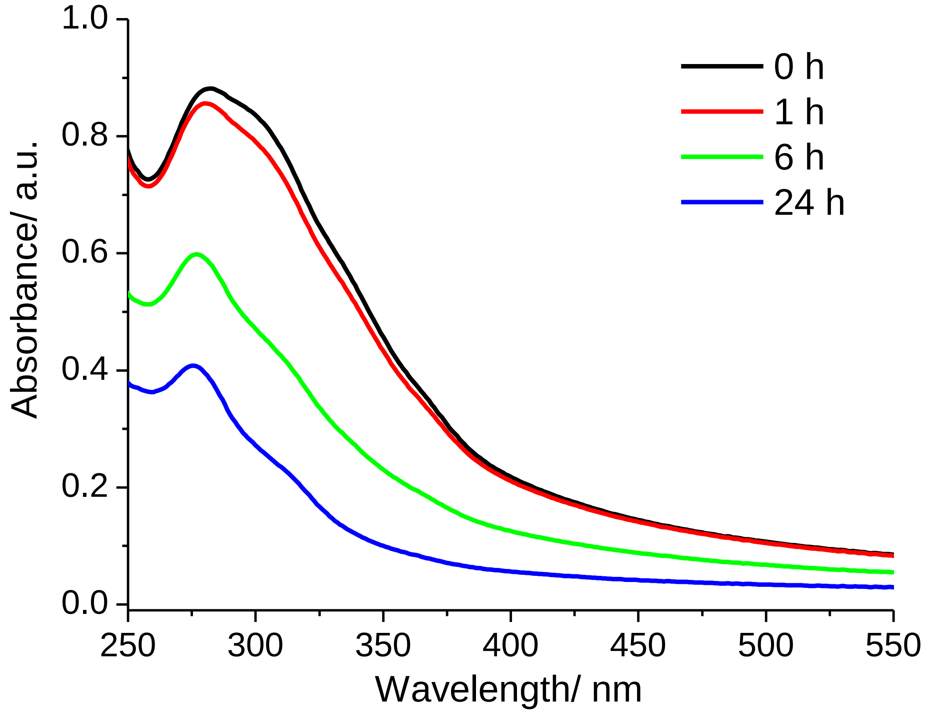

Figure S37. UV-Vis spectrum of **7** (25 μM) in PBS:DMSO (200:1) over the course of 24 h at 37 °C.

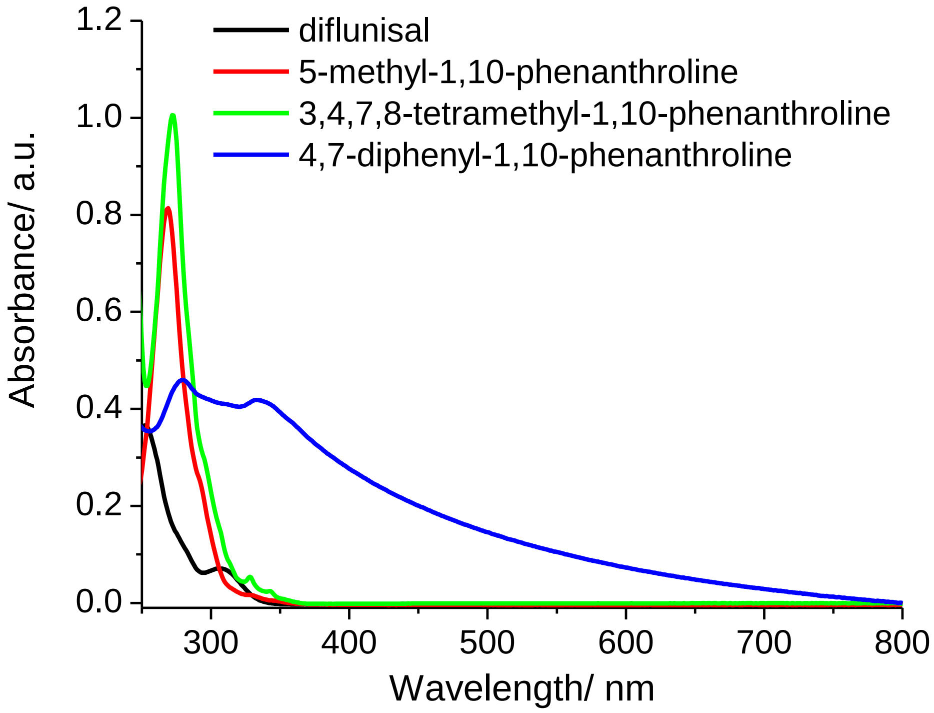

Figure S38. UV-Vis spectra of diflunisal, 5-methyl-1,10-phenanthroline, 3,4,7,8-tetramethyl-1,10-phenanthroline, and 4,7-diphenyl-1,10-phenanthroline (25 μM) in PBS:DMSO (200:1) at 37 ^{o}C.

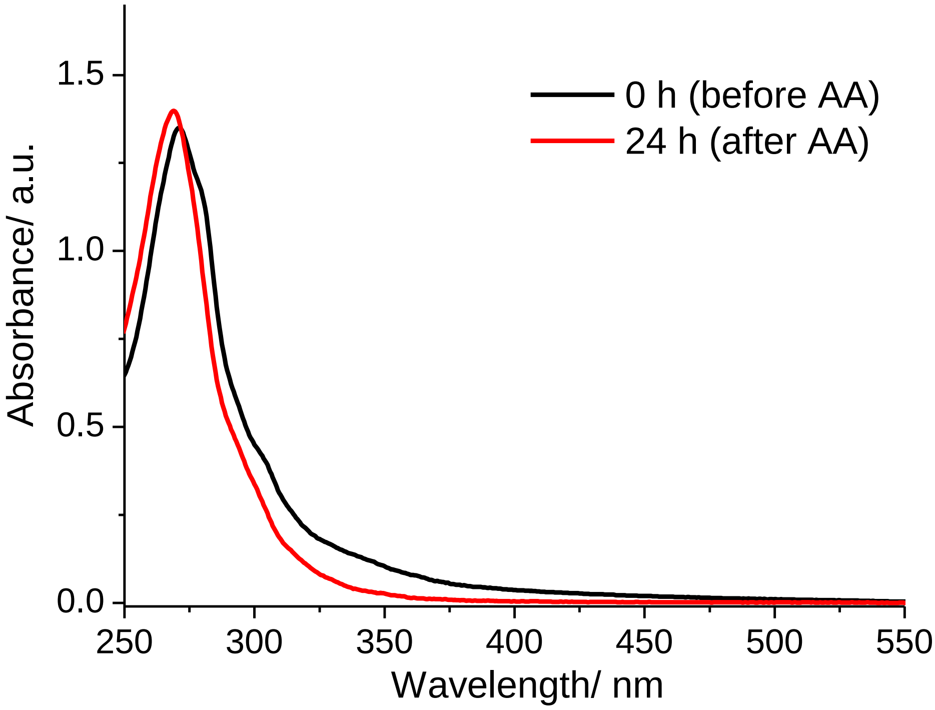

Figure S39. UV-Vis spectrum of **5** (25 μM) in the presence of ascorbic acid (250 μM) in PBS:DMSO (200:1) before and after incubation for 24 h at 37 ^{o}C.

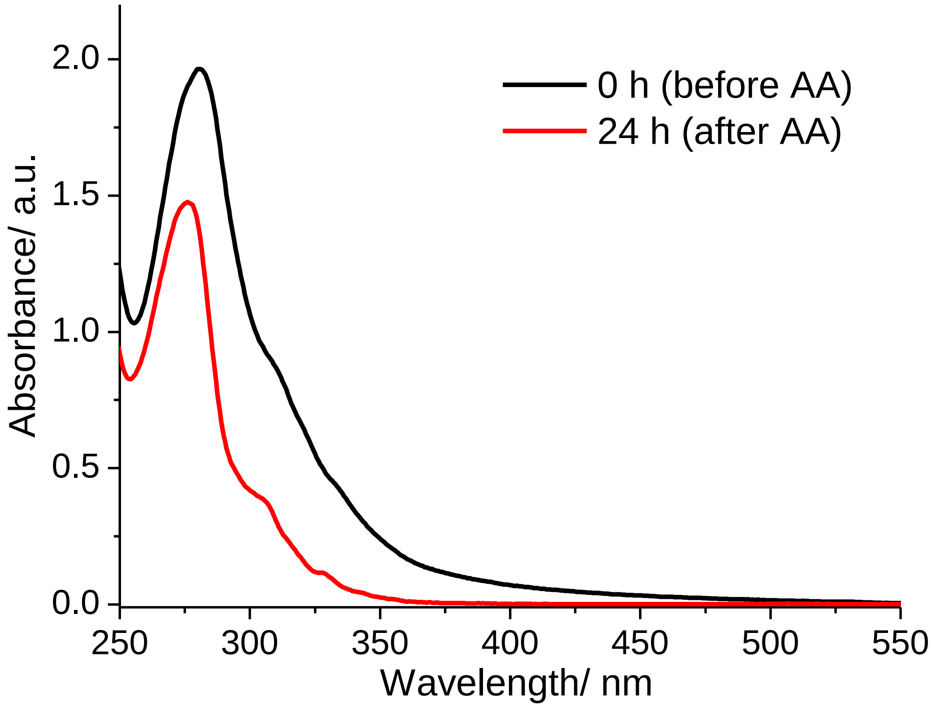

Figure S40. UV-Vis spectrum of **6** (25 µM) in the presence of ascorbic acid (250 µM) in PBS:DMSO (200:1) before and after incubation for 24 h at 37 ºC.

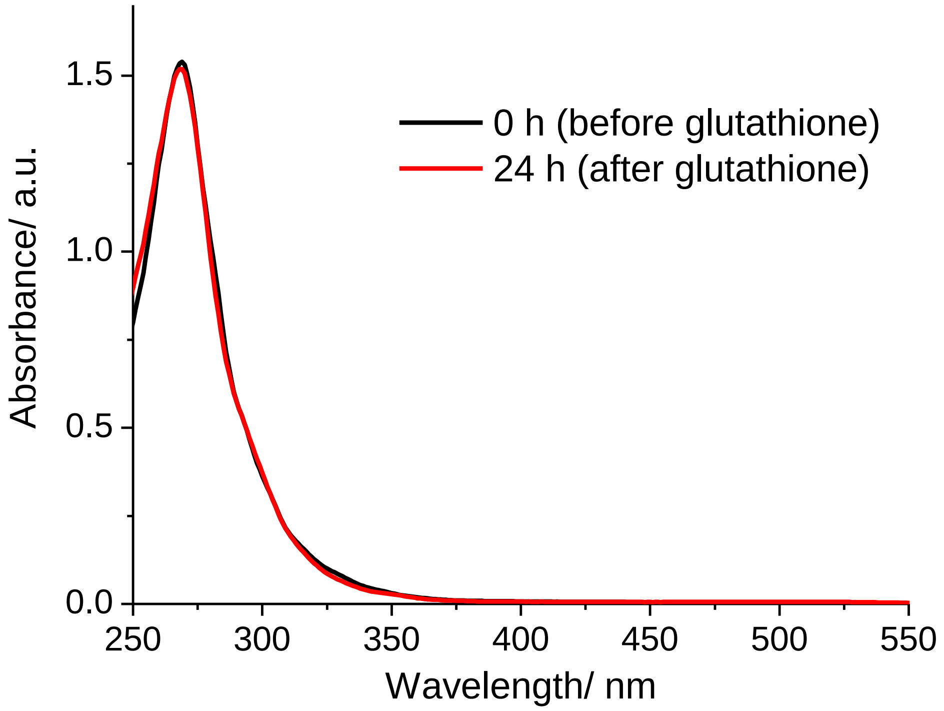

Figure S41. UV-Vis spectrum of **5** (25 µM) in the presence of glutathione (250 µM) in PBS:DMSO (200:1) before and after incubation for 24 h at 37 ºC.

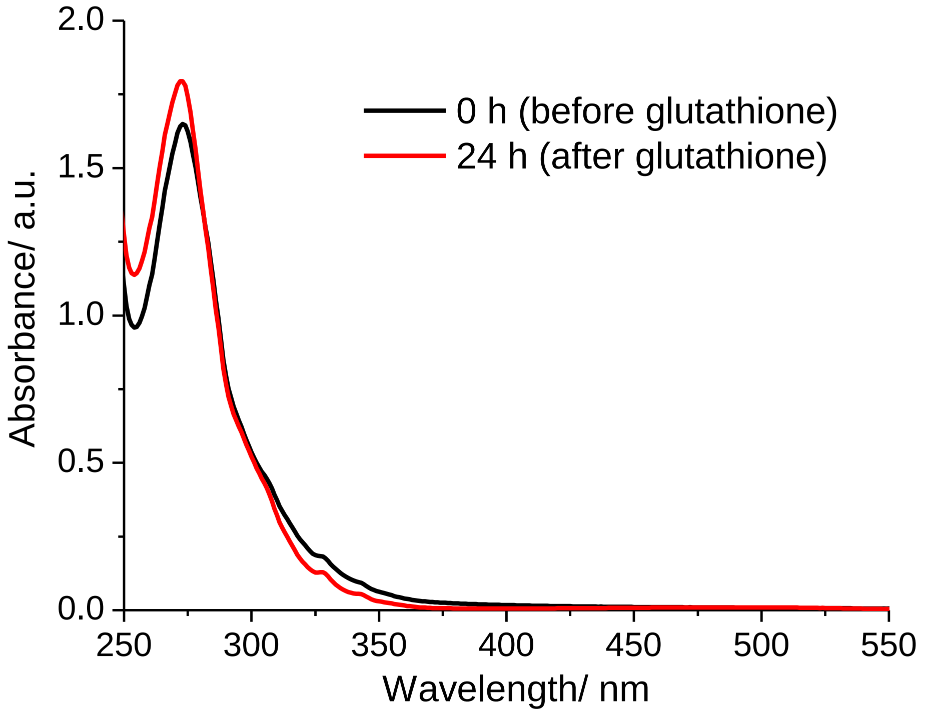

Figure S42. UV-Vis spectrum of **6** (25 µM) in the presence of glutathione (250 µM) in PBS:DMSO (200:1) before and after incubation for 24 h at 37 ºC.

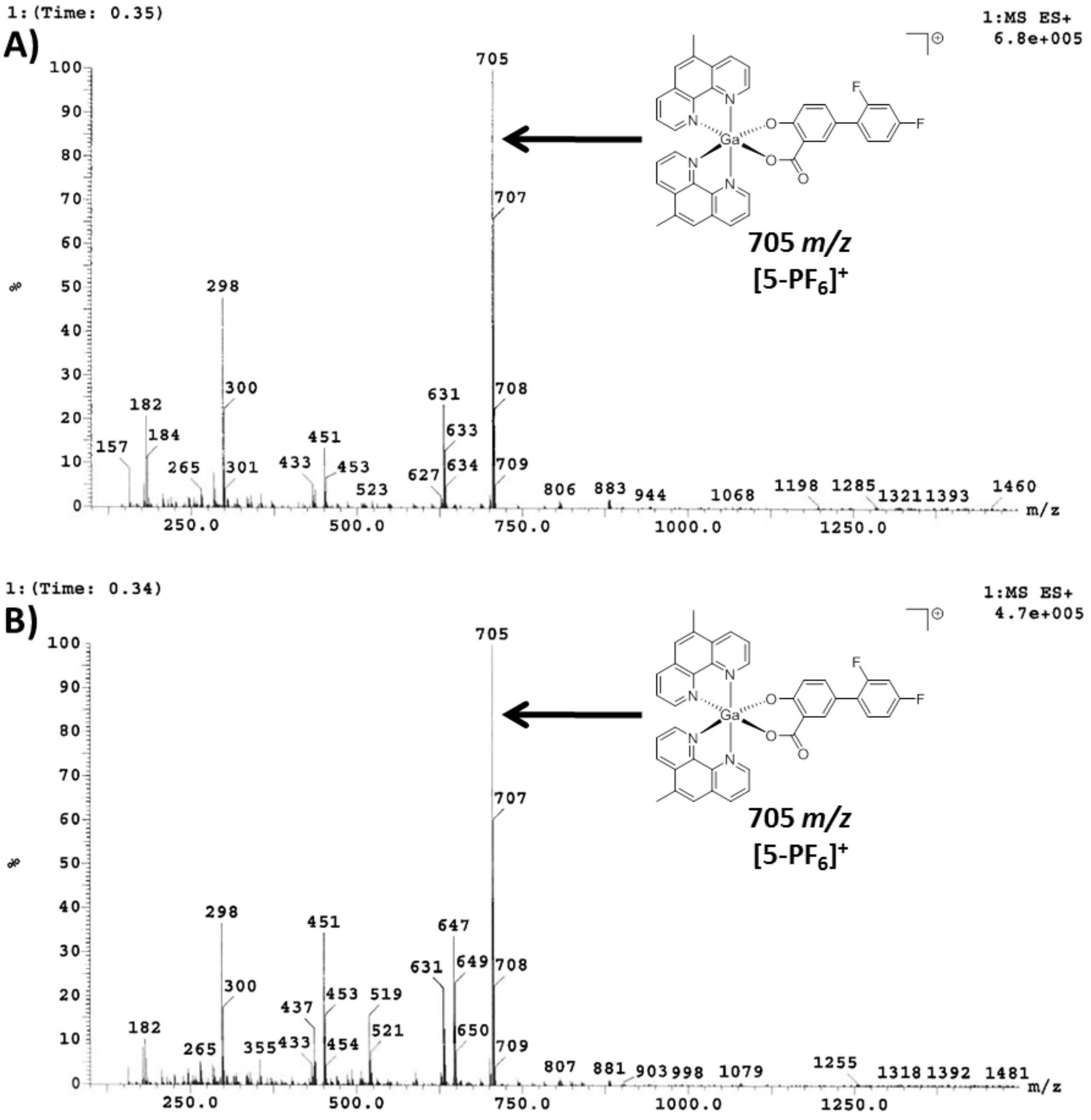

Figure S43. ESI mass spectra (positive mode) of **5** (500 μM) in H_2O:DMSO (10:1) in the presence of ascorbic acid (5 mM) (A) before and (B) after incubation for 24 h at 37 ^oC.

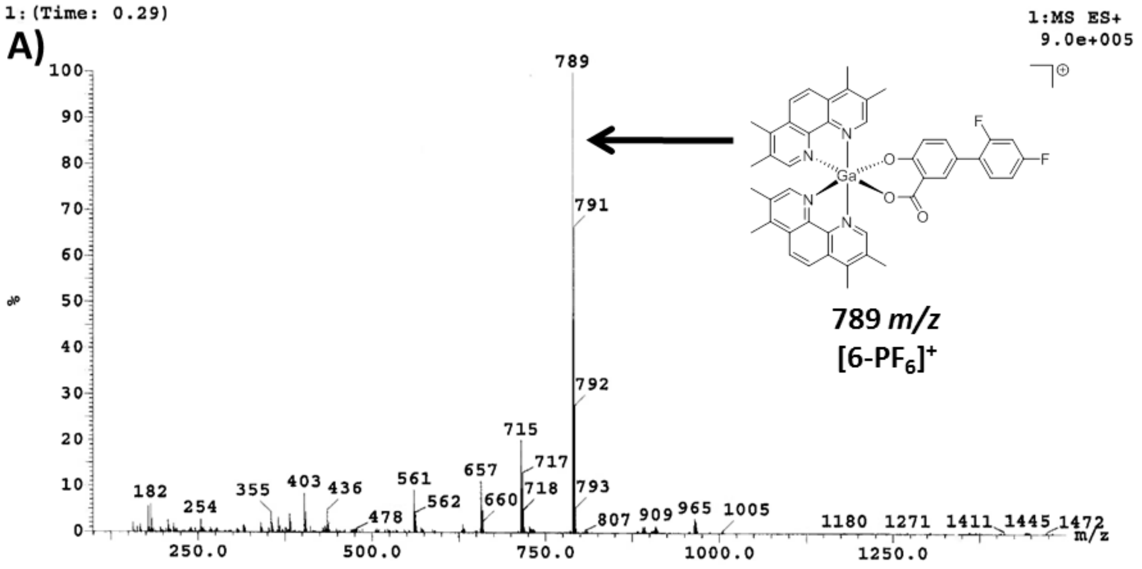

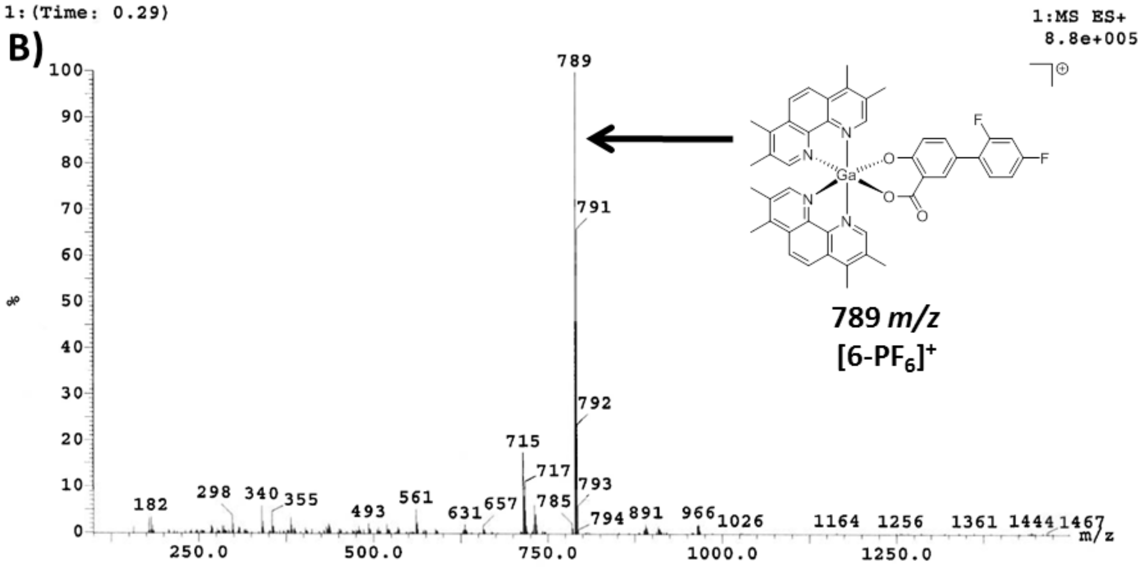

Figure S44. ESI mass spectra (positive mode) of **6** (500 μM) in H_2O:DMSO (10:1) in the presence of ascorbic acid (5 mM) (A) before and (B) after incubation for 24 h at 37 ^oC.

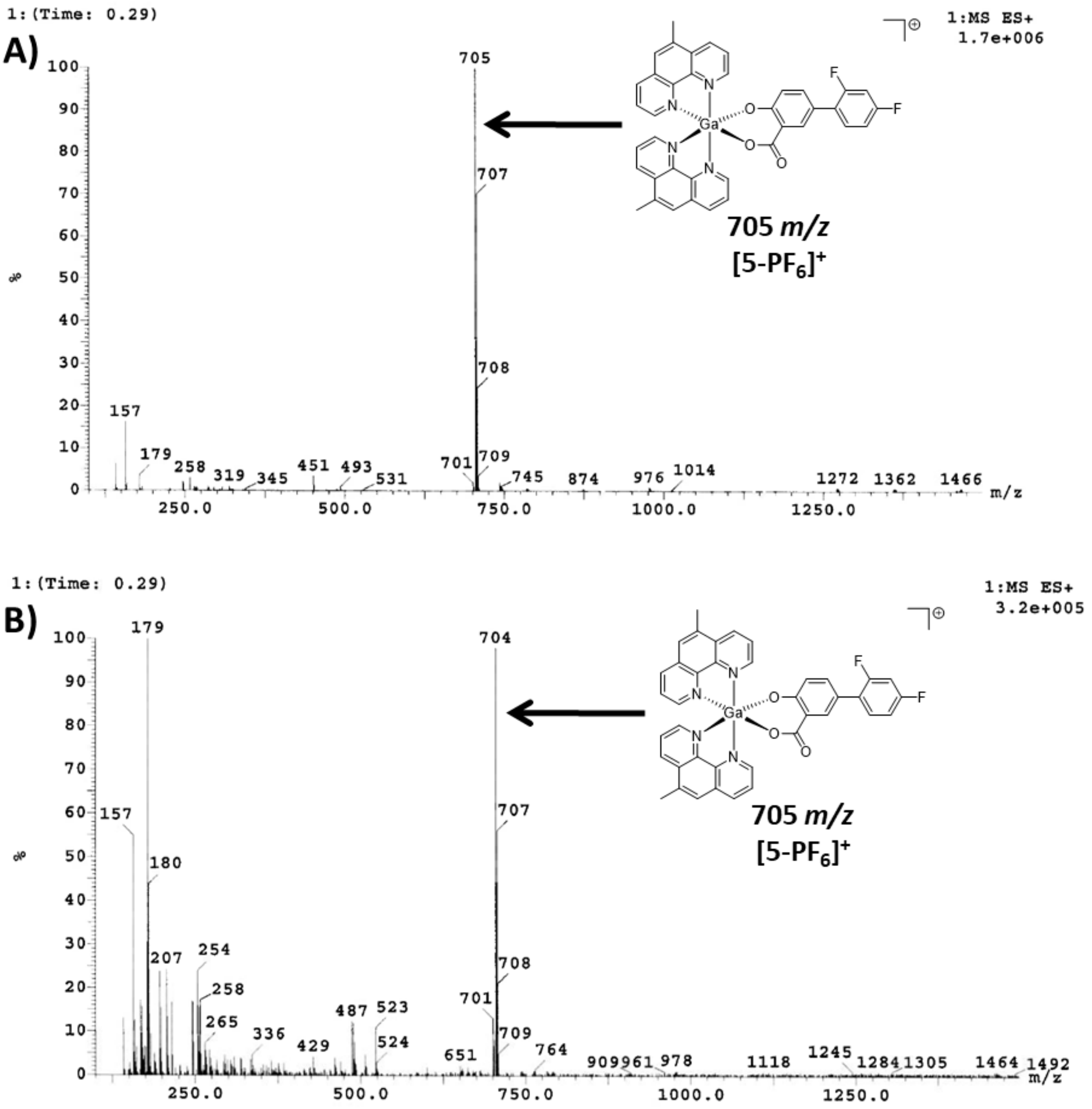

Figure S45. ESI mass spectra (positive mode) of **5** (500 μM) in H_2O:DMSO (10:1) in the presence of glutathione (5 mM) (A) before and (B) after incubation for 24 h at 37 ^{o}C.

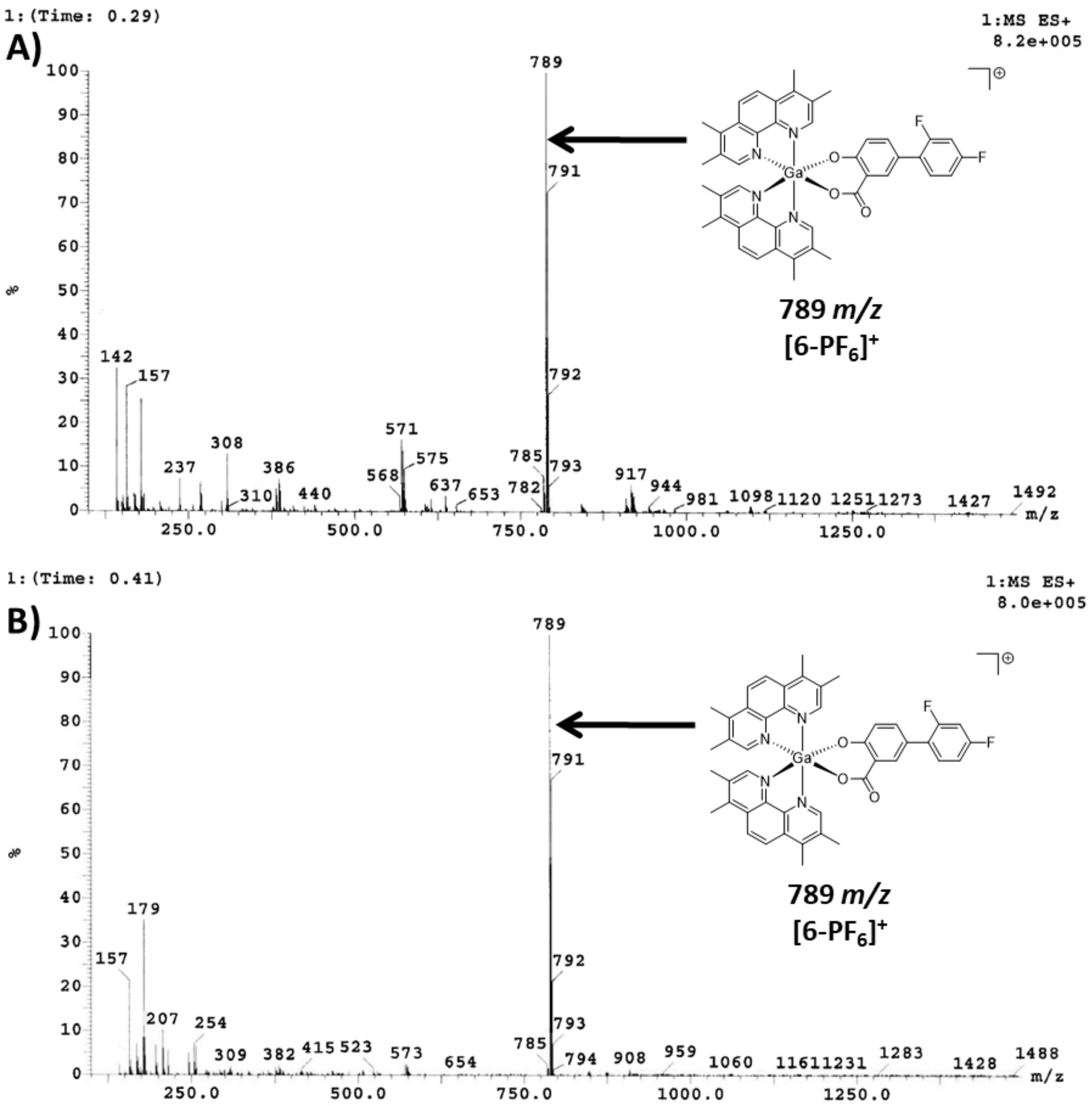

Figure S46. ESI mass spectra (positive mode) of **6** (500 μM) in H_2O:DMSO (10:1) in the presence of glutathione (5 mM) (A) before and (B) after incubation for 24 h at 37 ^oC.

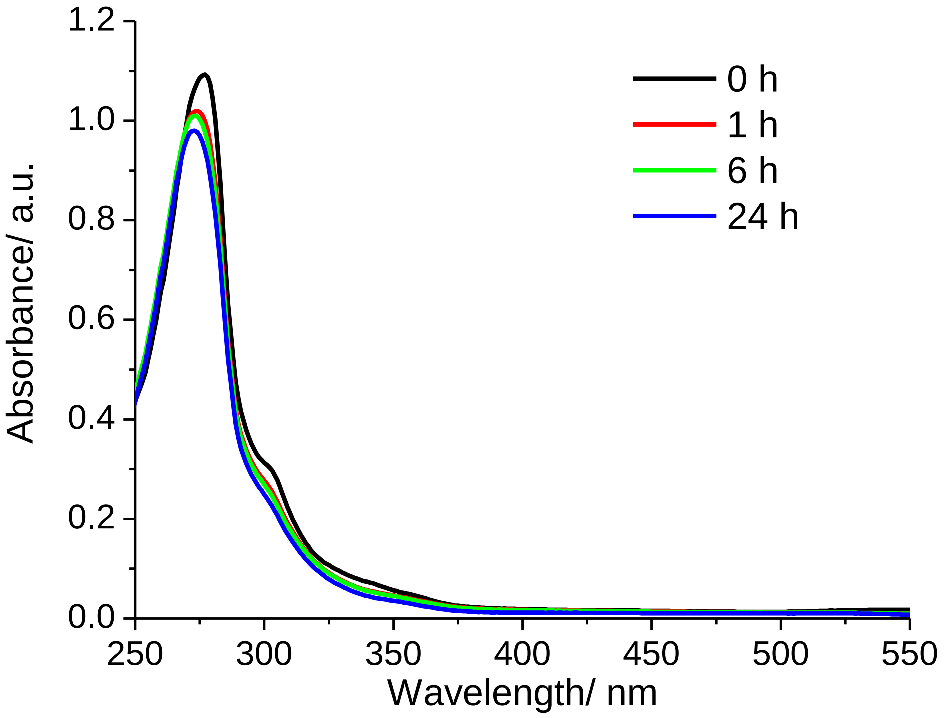

Figure S47. UV-Vis spectrum of **5** (25 μM) in sodium acetate buffer solution (pH 5.2):DMSO (200:1) over the course of 24 h at 37 ^{o}C.

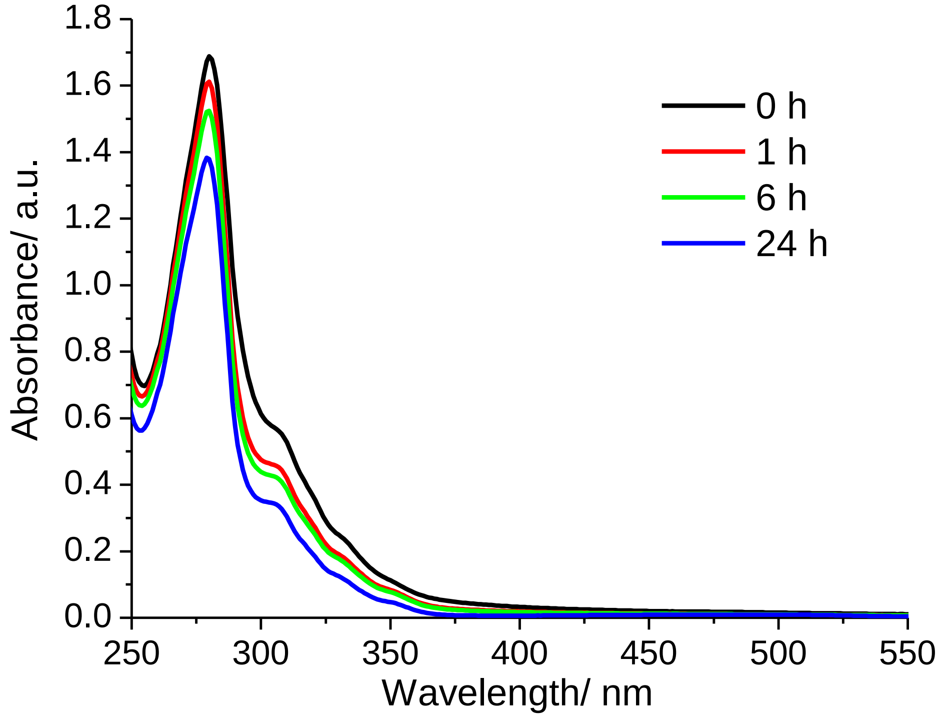

Figure S48. UV-Vis spectrum of **6** (25 μM) in sodium acetate buffer solution (pH 5.2):DMSO (200:1) over the course of 24 h at 37 ^{o}C.

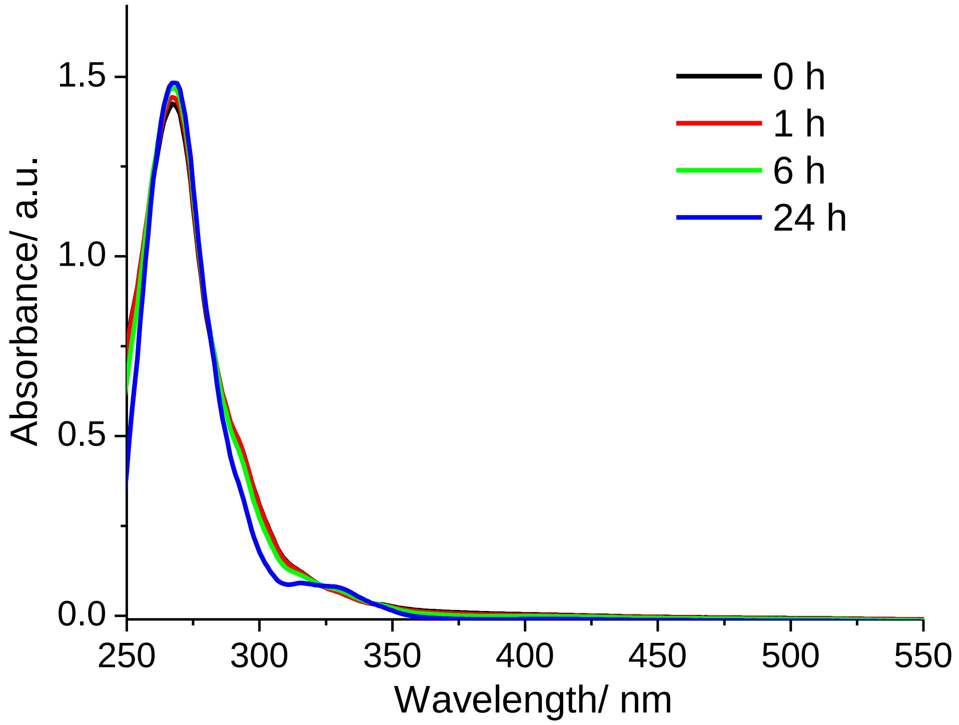

Figure S49. UV-Vis spectrum of **5** (25 μM) in DMEM:DMSO (200:1) over the course of 24 h at 37 ^{o}C.

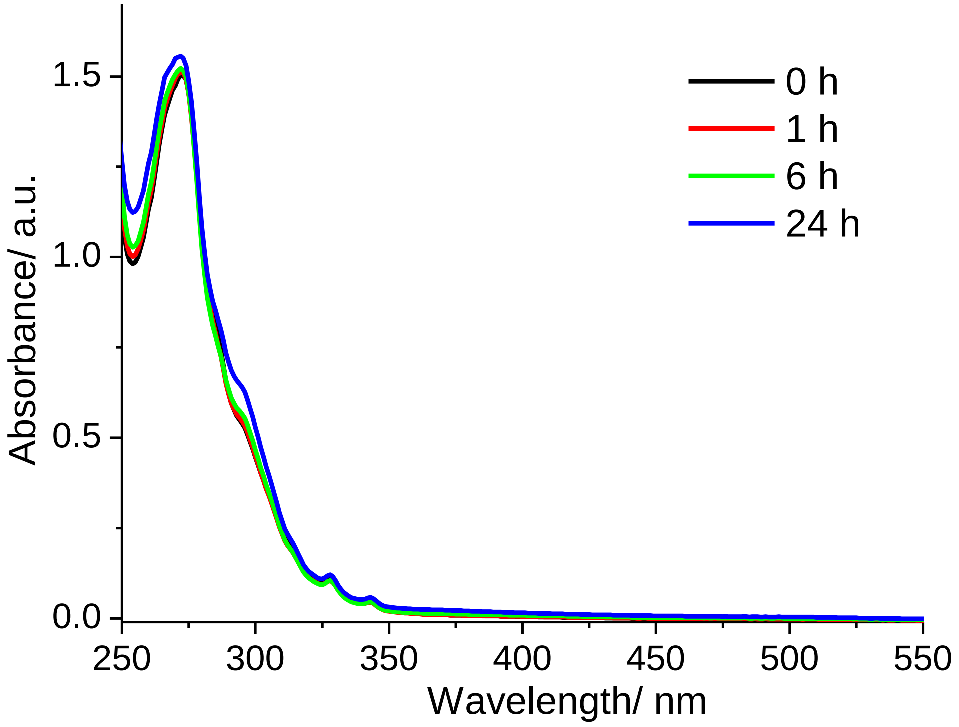

Figure S50. UV-Vis spectrum of **6** (25 μM) in DMEM:DMSO (200:1) over the course of 24 h at 37 ^{o}C.

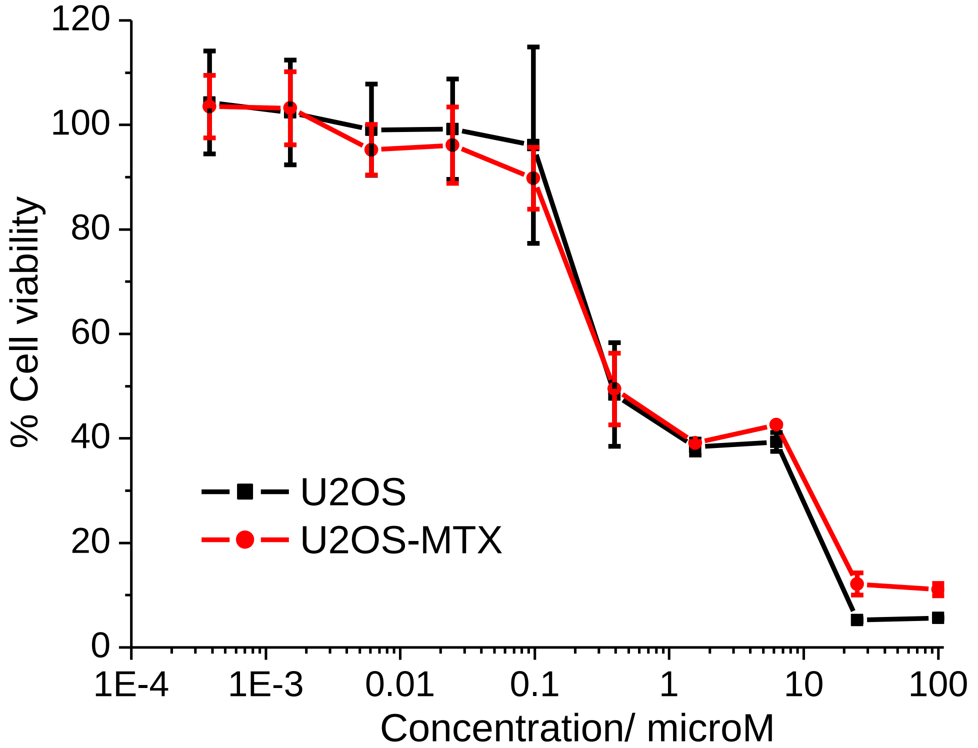

Figure S51. Representative dose-response curves for the treatment of U2OS or U2OS-MTX cells with **5** after 72 h incubation.

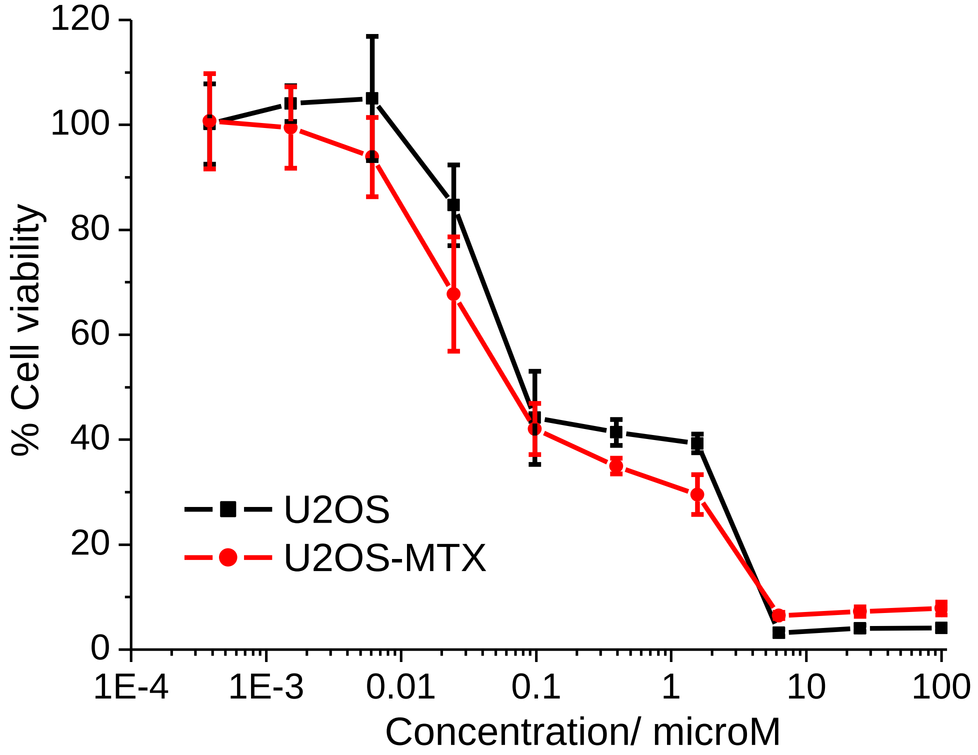

Figure S52. Representative dose-response curves for the treatment of U2OS or U2OS-MTX cells with **6** after 72 h incubation.

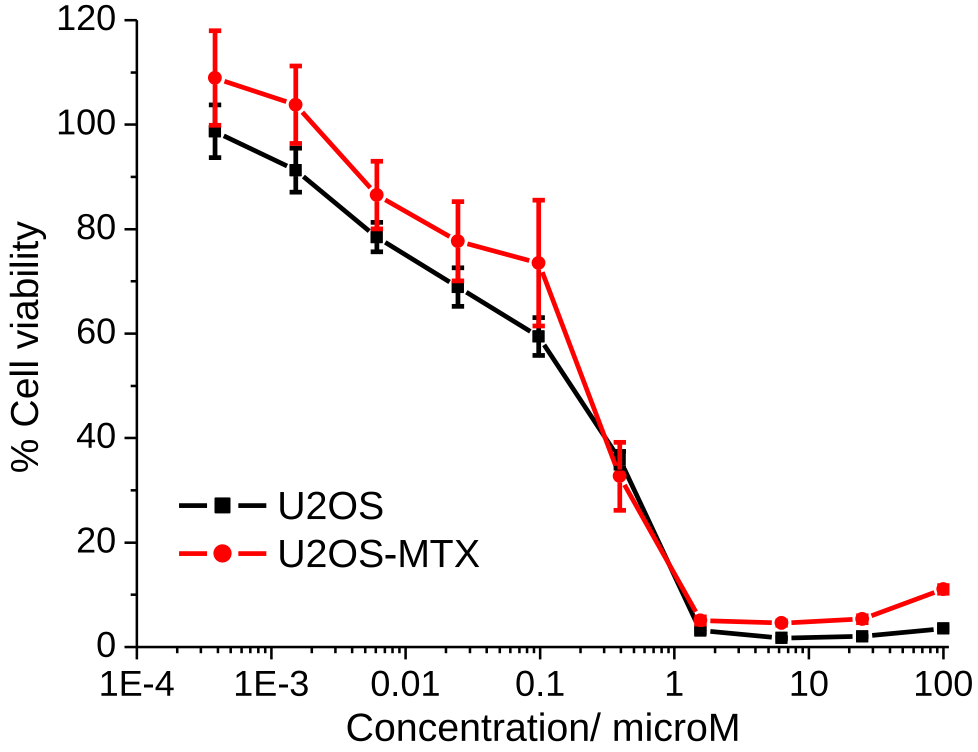

Figure S53. Representative dose-response curves for the treatment of U2OS or U2OS-MTX cells with doxorubicin after 72 h incubation.

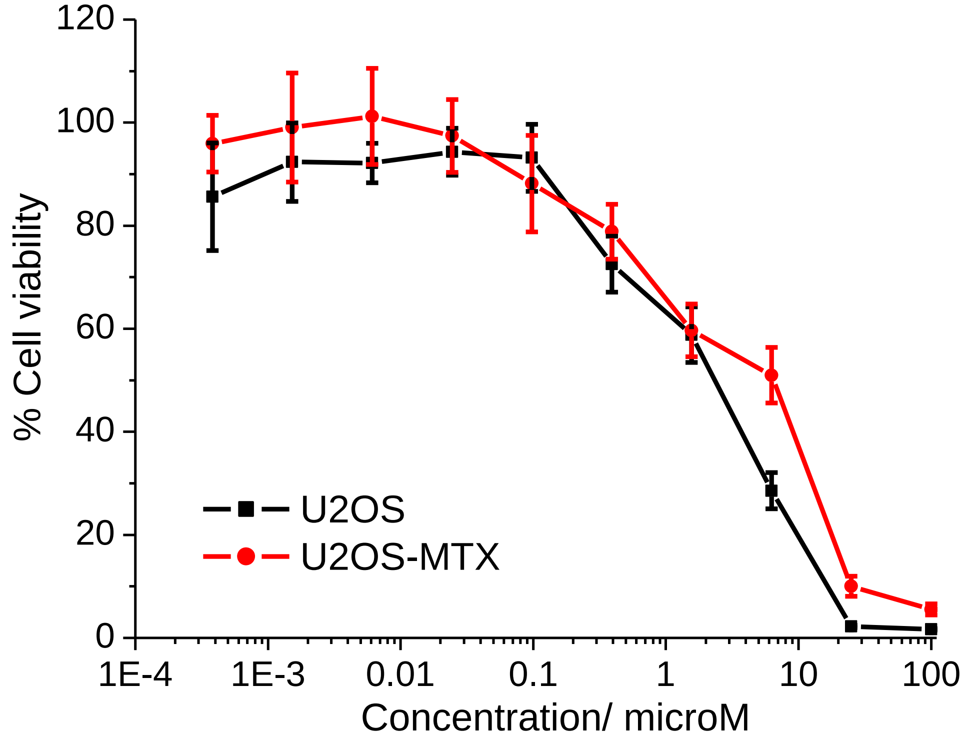

Figure S54. Representative dose-response curves for the treatment of U2OS or U2OS-MTX cells with etoposide after 72 h incubation.

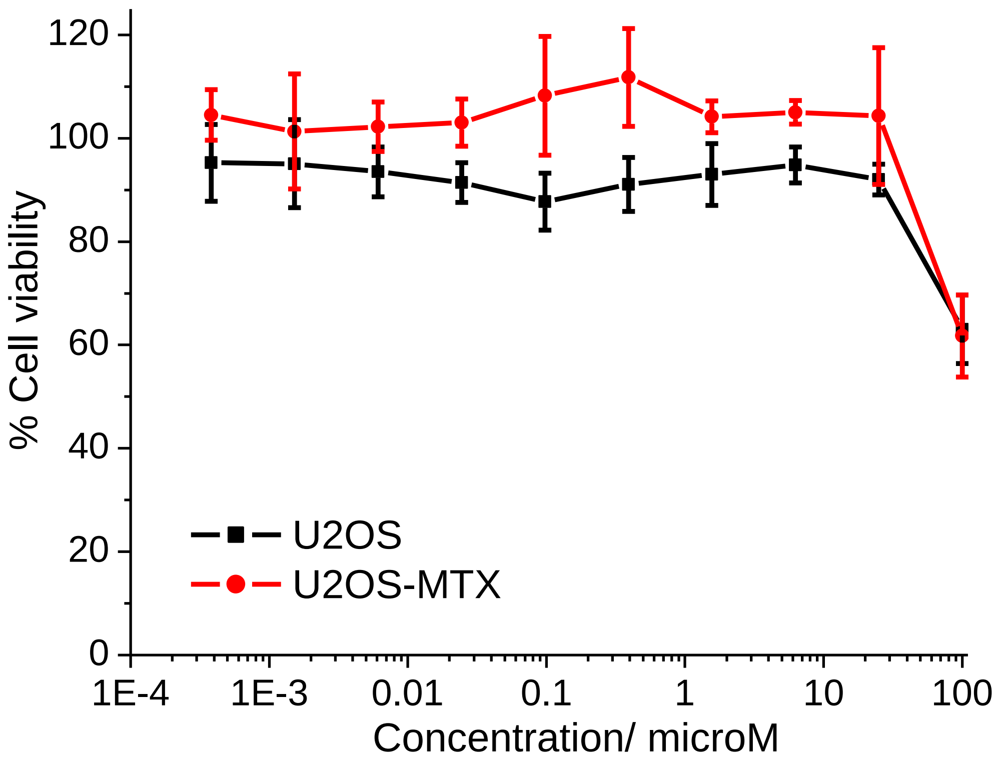

Figure S55. Representative dose-response curves for the treatment of U2OS or U2OS-MTX cells with ifosfamide after 72 h incubation.

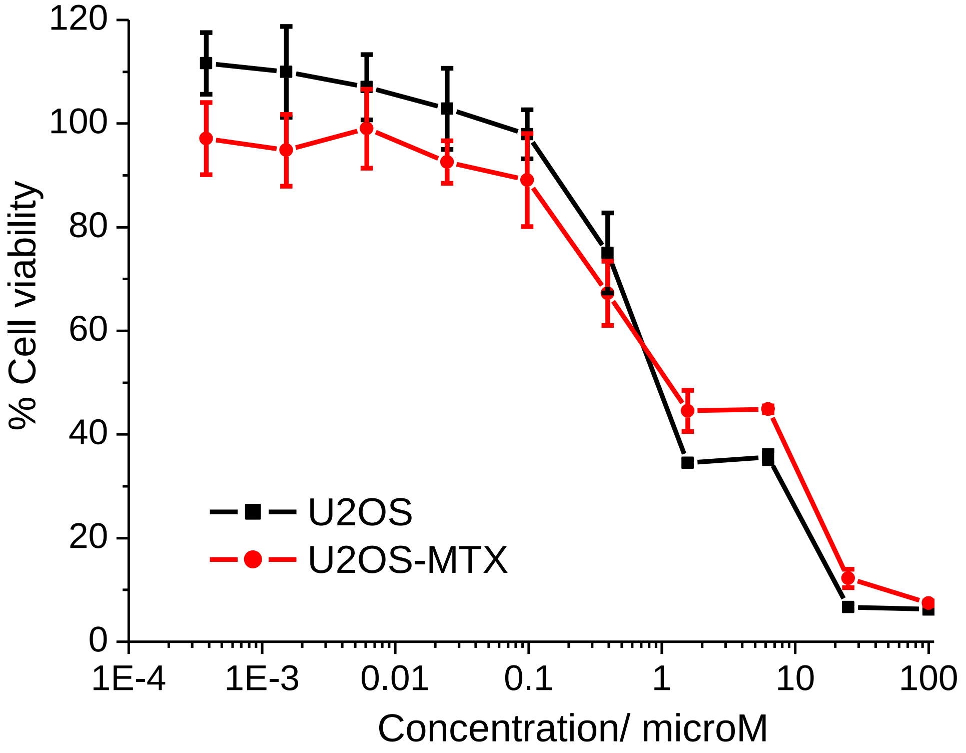

Figure S56. Representative dose-response curves for the treatment of U2OS or U2OS-MTX cells with **2** after 72 h incubation.

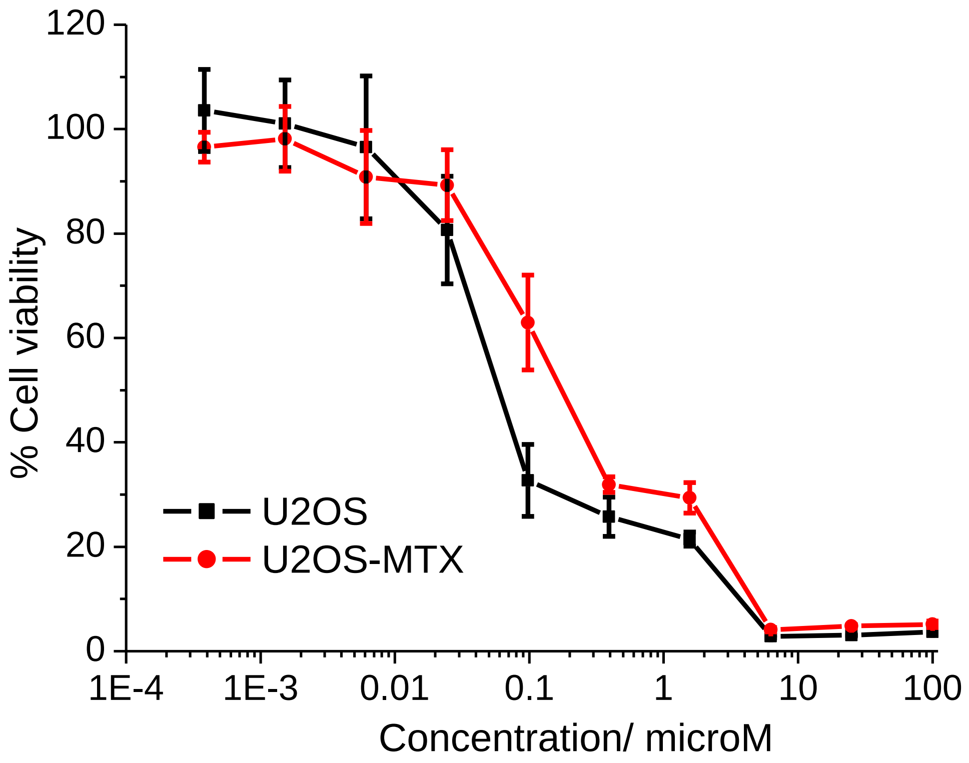

Figure S57. Representative dose-response curves for the treatment of U2OS or U2OS-MTX cells with **3** after 72 h incubation.

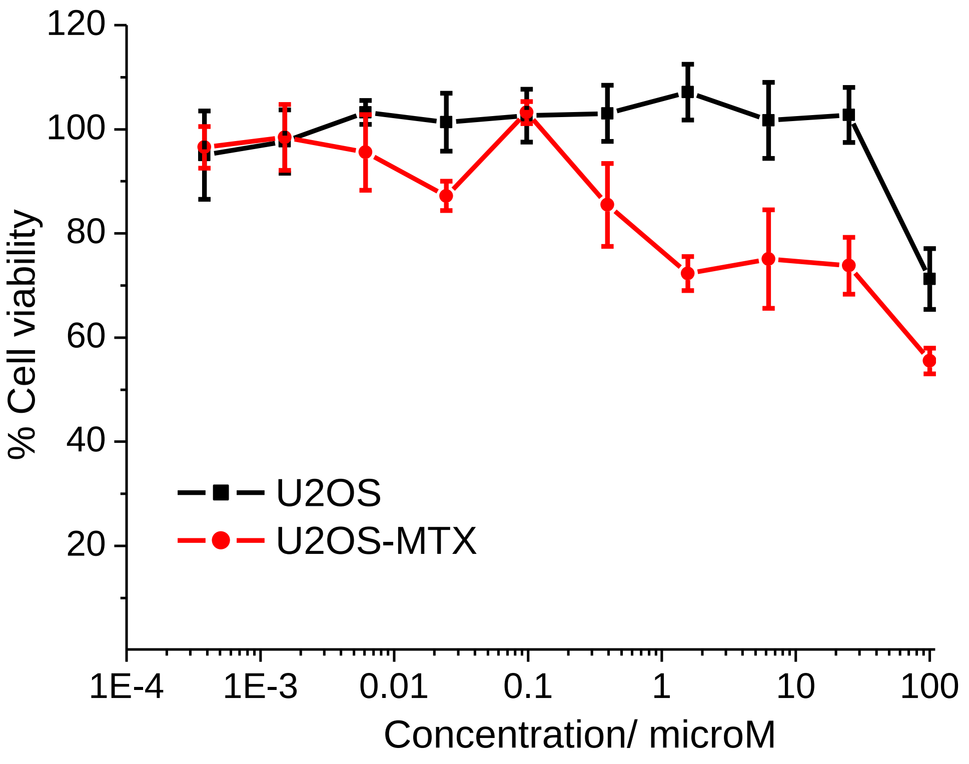

Figure S58. Representative dose-response curves for the treatment of U2OS or U2OS-MTX cells with diflunisal after 72 h incubation.

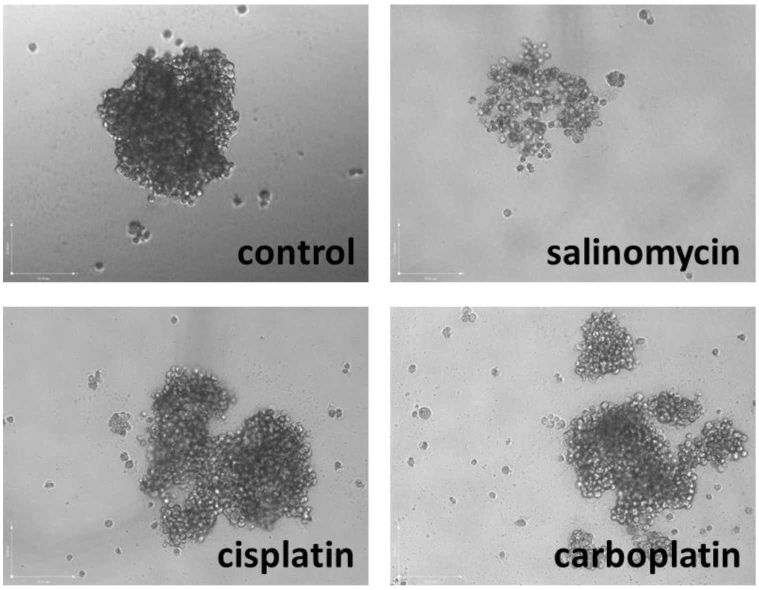

Figure S59. Representative bright-field images (× 10) of U2OS-MTX sarcospheres in the absence and presence of salinomycin, cisplatin, carboplatin at its IC_{20} value (10 days incubation).

Table S6. IC_{50} values of the gallium complexes, **2-3** and **5-6**, diflunisal, cisplatin, carboplatin, and salinomycin against U2OS-MTX sarcospheres.

Compound	OSC-sarcosphere IC_{50} [μM] [a]
**2**	3.93 ± 0.04
**3**	4.28 ± 0.03
**5**	0.97 ± 0.01
**6**	0.97 ± 0.01
diflunisal	59.75 ± 0.55
cisplatin [b]	16.49 ± 0.20
carboplatin [b]	22.77 ± 0.09
salinomycin [b]	4.70 ± 0.08

[a] Determined after 10 days incubation (mean of three independent experiments ± SD). [b] Reported in reference [2].

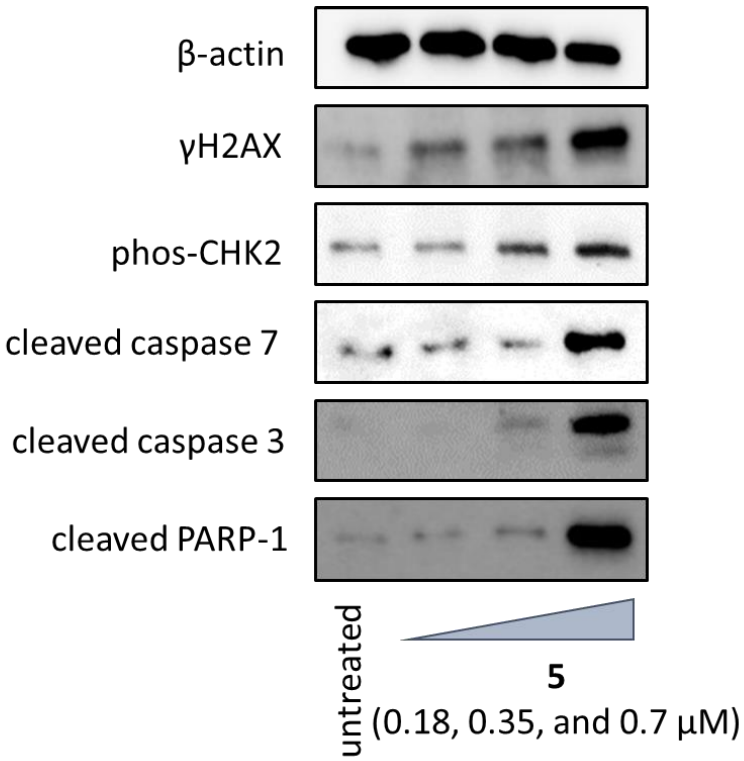

Figure S60. Immunoblotting analysis of proteins related to the DNA damage and apoptosis pathways. Protein expression in U2OS cells following treatment with **5** (0.18, 0.35, and 0.7 µM for 72 h).

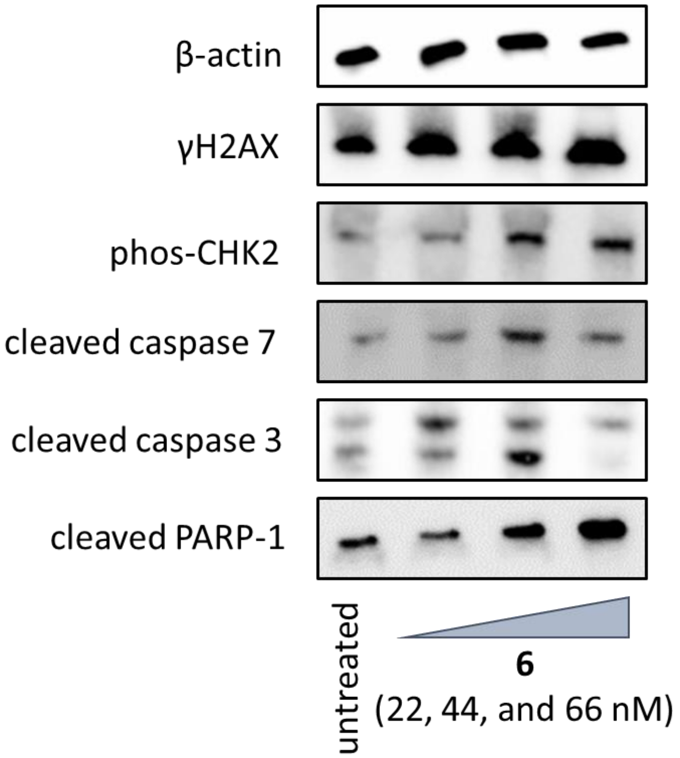

Figure S61. Immunoblotting analysis of proteins related to the DNA damage and apoptosis pathways. Protein expression in U2OS cells following treatment with **6** (22, 44, and 66 nM for 72 h).

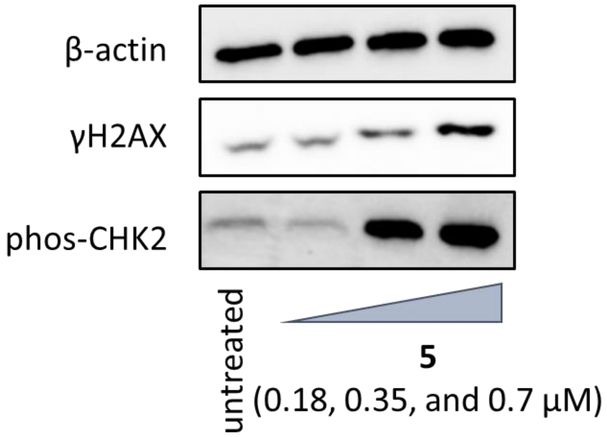

Figure S62. Immunoblotting analysis of proteins related to the DNA damage pathway. Protein expression in U2OS cells following treatment with **5** (0.18, 0.35, and 0.7 µM for 24 h).

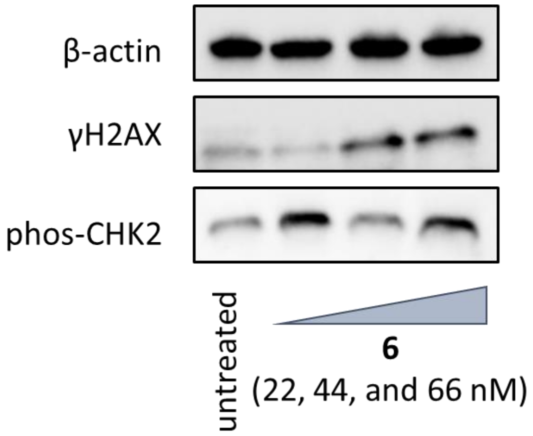

Figure S63. Immunoblotting analysis of proteins related to the DNA damage pathway. Protein expression in U2OS cells following treatment with **6** (22, 44, and 66 nM for 24 h).

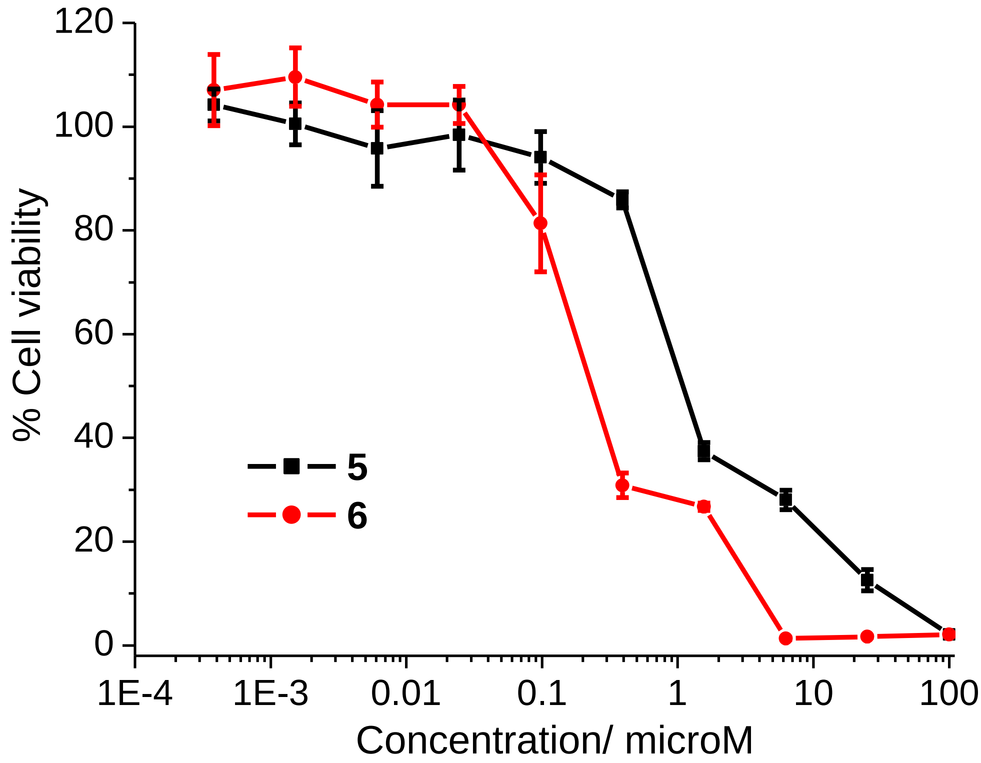

Figure S64. Representative dose-response curves for the treatment of U2OS cells with **5** or **6** after 72 h incubation in the presence of z-VAD-FMK (5 μM).

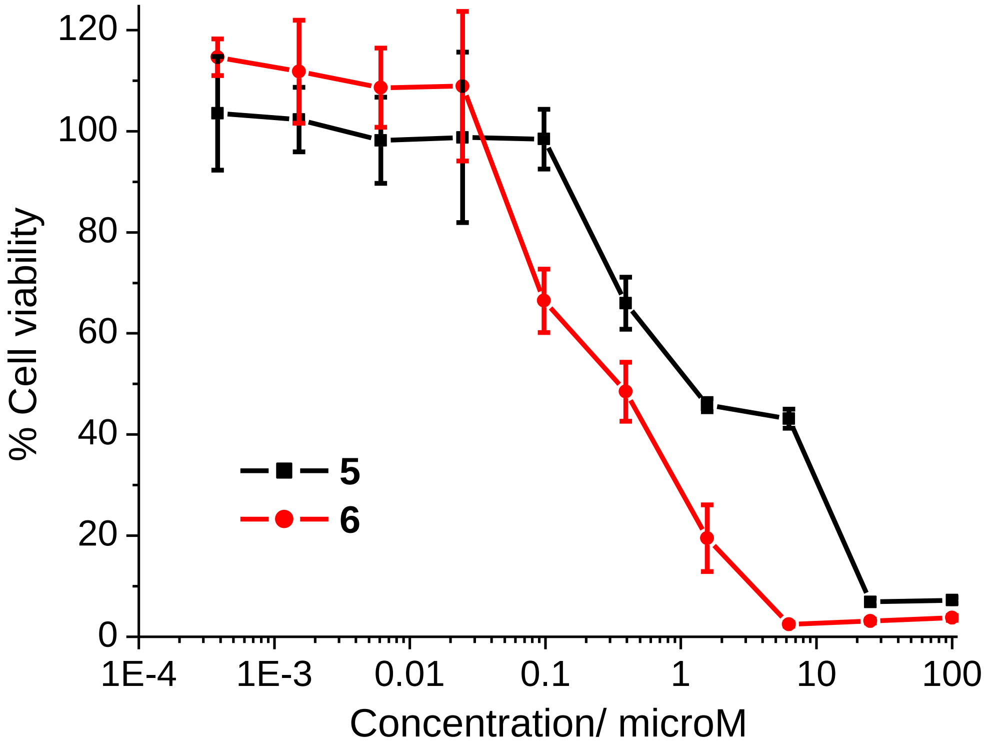

Figure S65. Representative dose-response curves for the treatment of U2OS-MTX cells with **5** or **6** after 72 h incubation in the presence of z-VAD-FMK (5 μM).

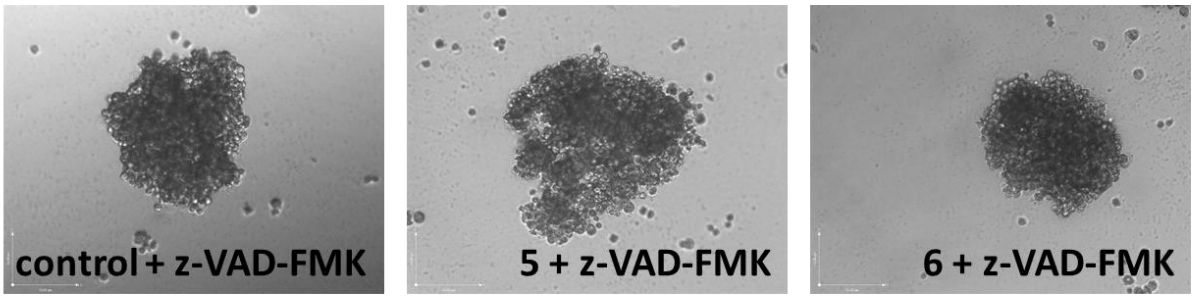

Figure S66. Representative bright-field images (x 10) of U2OS-MTX sarcospheres treated with z-VAD-FMK (5 µM) in the absence and presence of **5** or **6** at their respective IC_{20} values for 10 days.

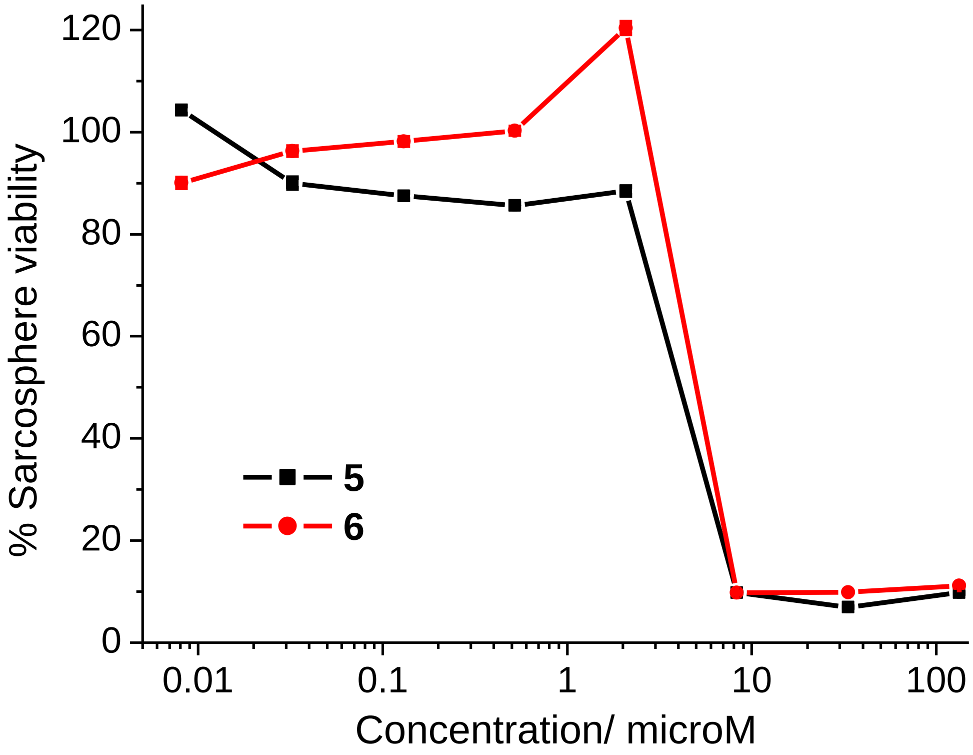

Figure S67. Representative dose-response curves for the treatment of U2OS-MTX sarcospheres with **5** or **6** in the presence of z-VAD-FMK (5 µM) after 10 days incubation.

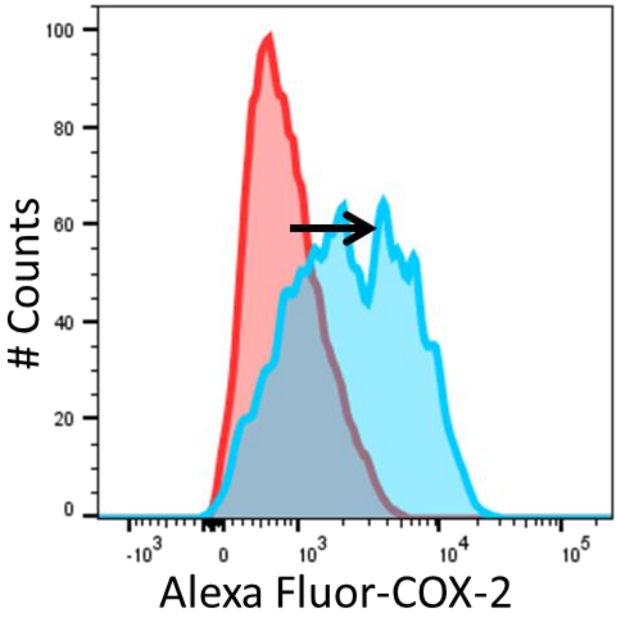

Figure S68. Representative histograms displaying the green fluorescence emitted by anti-COX-2 Alexa Fluor 488 nm antibody-stained U2OS-MTX cells untreated (red) and treated (blue) with LPS (2.5 µM) for 24 h, followed by 48 h in fresh media.

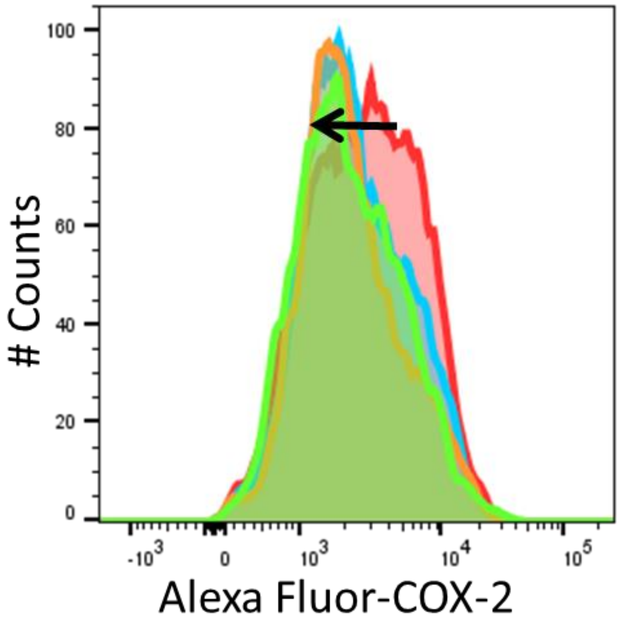

Figure S69. Representative histograms displaying the green fluorescence emitted by anti-COX-2 Alexa Fluor 488 nm antibody-stained U2OS-MTX cells treated with LPS (2.5 µM) for 24 h, followed by 48 h in fresh media (red) or media containing diflunisal (10µM, blue; 20 µM, orange; 40 µM, green).

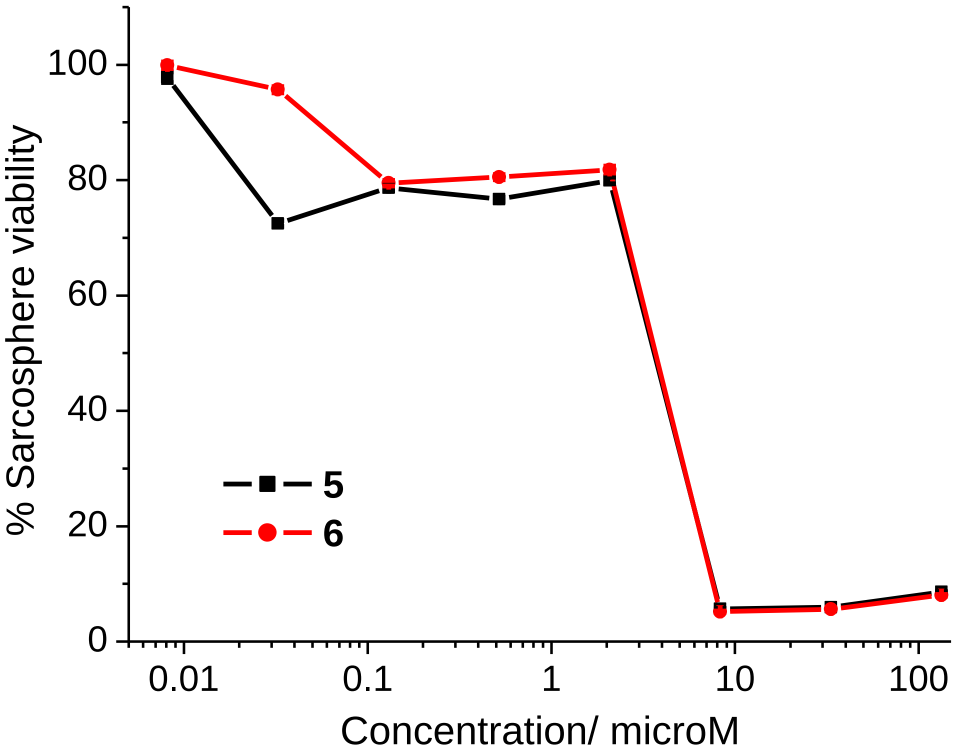

Figure S70. Representative dose-response curves for the treatment of U2OS-MTX sarcospheres with **5** or **6** in the presence of PGE2 (20 µM) after 10 days incubation.
